# PRMT5-mediated FUBP1 methylation accelerates prostate cancer progression

**DOI:** 10.1172/JCI175023

**Published:** 2024-08-15

**Authors:** Weiwei Yan, Xun Liu, Xuefeng Qiu, Xuebin Zhang, Jiahui Chen, Kai Xiao, Ping Wu, Chao Peng, Xiaolin Hu, Zengming Wang, Jun Qin, Liming Sun, Luonan Chen, Denglong Wu, Shengsong Huang, Lichen Yin, Zhenfei Li

**Affiliations:** 1 Key Laboratory of Multi-Cell Systems, Shanghai Institute of Biochemistry and Cell Biology, Center for Excellence in Molecular Cell Science, Chinese Academy of Sciences, University of Chinese Academy of Sciences, Shanghai, China.; 2 Institute of Functional Nano and Soft Materials (FUNSOM), Jiangsu Key Laboratory for Carbon-Based Functional Materials and Devices, Collaborative Innovation Center of Suzhou Nano Science and Technology, Soochow University, Suzhou, China.; 3 Department of Urology, Drum Tower Hospital, Medical School of Nanjing University, Institute of Urology, Nanjing University, Nanjing, China.; 4 National Facility for Protein Science Shanghai, Shanghai Advanced Research Institute, Chinese Academy of Sciences, Shanghai, China.; 5 State Key Laboratory of Systems Medicine for Cancer, Center for Single-Cell Omics, School of Public Health, Shanghai Jiao Tong University School of Medicine, Shanghai, China.; 6 CAS Key Laboratory of Tissue Microenvironment and Tumor, Shanghai Institute of Nutrition and Health, University of Chinese Academy of Sciences, Chinese Academy of Sciences, Shanghai, China.; 7 Department of Urology, Tongji Hospital, School of Medicine, Tongji University, Shanghai, China.; 8 Key Laboratory of Systems Health Science of Zhejiang Province, School of Life Science, Hangzhou Institute for Advanced Study, University of Chinese Academy of Sciences, Hangzhou, China.

**Keywords:** Oncology, Therapeutics, Peptides, Prostate cancer

## Abstract

Strategies beyond hormone-related therapy need to be developed to improve prostate cancer mortality. Here, we show that FUBP1 and its methylation were essential for prostate cancer progression, and a competitive peptide interfering with FUBP1 methylation suppressed the development of prostate cancer. FUBP1 accelerated prostate cancer development in various preclinical models. PRMT5-mediated FUBP1 methylation, regulated by BRD4, was crucial for its oncogenic effect and correlated with earlier biochemical recurrence in our patient cohort. Suppressed prostate cancer progression was observed in various genetic mouse models expressing the FUBP1 mutant deficient in PRMT5-mediated methylation. A competitive peptide, which was delivered through nanocomplexes, disrupted the interaction of FUBP1 with PRMT5, blocked FUBP1 methylation, and inhibited prostate cancer development in various preclinical models. Overall, our findings suggest that targeting FUBP1 methylation provides a potential therapeutic strategy for prostate cancer management.

## Abstract 2

Peptide blocking PRMT5 mediated FUBP1 methylation inhibits prostate cancer

## Graphical abstract

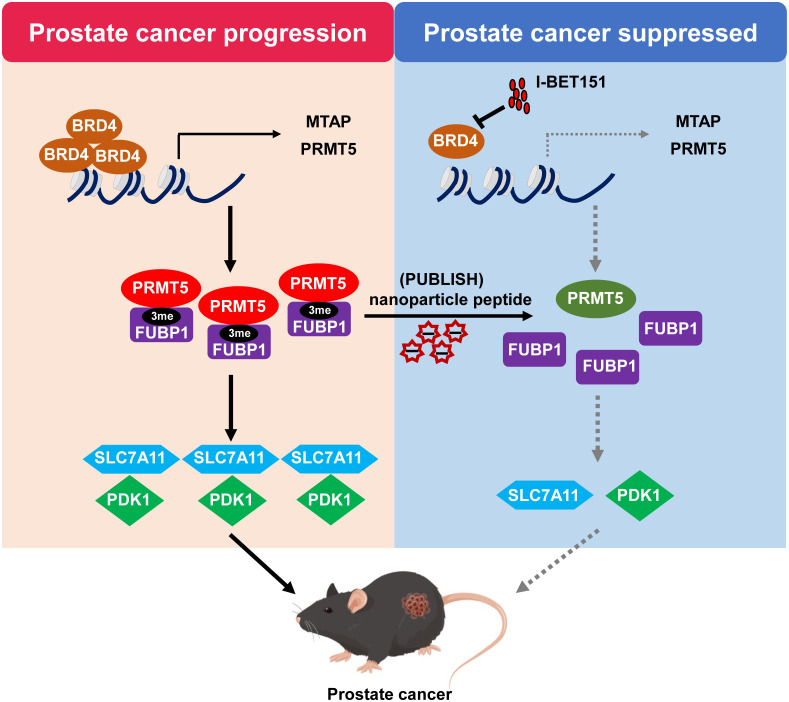

## Introduction

Prostate cancer is a major global health care challenge (1, 2). Androgens from the testis and the adrenal gland bind to androgen receptor (AR) to activate AR signaling and facilitate prostate cancer progression (3, 4). Thus, the androgen biosynthesis pathway and AR signaling are the main therapeutic targets for prostate cancer (5–9). Hormonal therapy has been the principal treatment for decades. Although it is initially effective, patients inevitably develop treatment resistance due to increasing tumor heterogeneity (10–12). Consequently, novel strategies beyond hormone therapy are required to improve the clinical benefits for prostate cancer patients (8, 13).

Transcriptional factors are recognized as potential therapeutic targets for prostate cancer, owing to its crucial role in a myriad of physiological and pathological processes (14–16). Gaining insight into key transcriptional factors implicated in prostate cancer and developing corresponding treatments would provide innovative approaches for disease management beyond hormone-related treatment (17–19). Far-upstream element (FUSE)–binding protein 1 (FUBP1) is a versatile DNA- and RNA-binding protein involved in gene transcription, RNA processing, and protein translation (20, 21). FUBP1 dysregulation has been reported in multiple cancers (22–24). FUBP1 deletion has been frequently found in oligodendroglioma and identified as a “long tail” driver, in tandem with *PTEN* deletion, for breast cancer (25, 26). On the other hand, elevated expression of FUBP1 was reported to promote progression of lung cancer, possibly by regulating c-Myc expression and alterative splicing (25, 27, 28). A subtle function of FUBP1 seems to be involved in different pathological environments, and its role in prostate cancer remains largely unexplored (29).

FUBP1 contains a consensus sequence for potential arginine methylation. Arginine methylation, in the form of monomethylarginine (MMA), asymmetric dimethylarginine (aDMA), and symmetric dimethylarginine (sDMA), regulates protein function (30–32). Protein arginine methyltransferases 1–9 (PRMT1–9) serve as the primary methylases, using *S*-adenosylmethionine (SAM) as the methyl donor (33). *S*-Methyl-5′-thioadenosine phosphorylase (MTAP) is vital for SAM generation, and MTAP deficiency results in the accumulation of methylthioadenosine (MTA), which inhibits PRMT activity (34, 35). Posttranslational modification may affect the function of FUBP1 in different pathological environments, but it has not been thoroughly investigated, especially in prostate cancer.

Here, we investigated the function and modification of FUBP1 in prostate cancer. We also developed a competitive peptide delivered by nanocomplexes to block FUBP1 methylation in vivo as a potential therapeutic approach for cancer treatment.

## Results

### Oncogenic effects and modifications of FUBP1 in prostate cancer.

**Figure 1:**
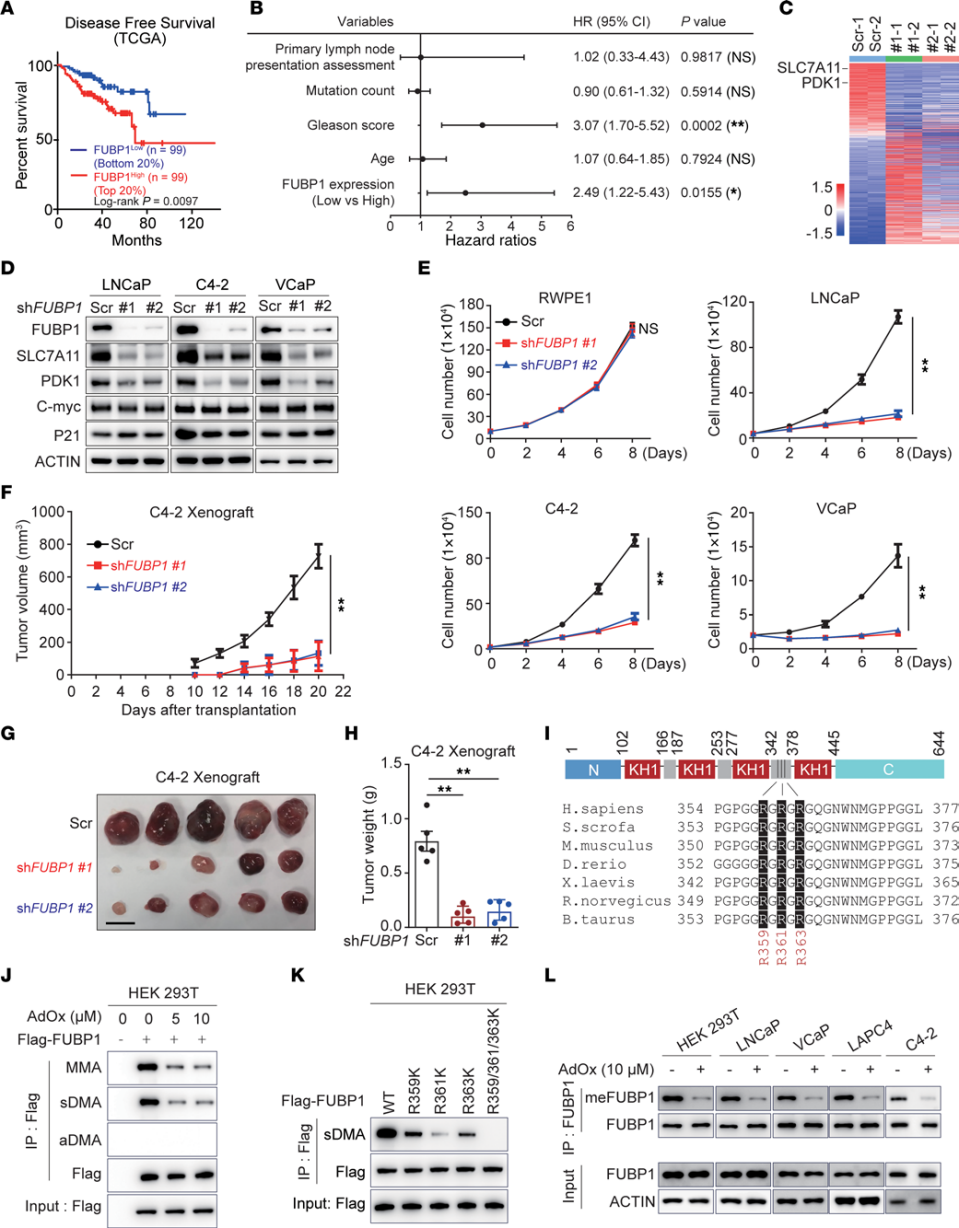
The oncogenic effect and modifications of FUBP1 in prostate cancer. (**A**) Correlation of FUBP1 levels with biochemical recurrence in The Cancer Genome Atlas (TCGA). Log-rank test. (**B**) Multivariate analysis of predictive factors for biochemical recurrence in TCGA data set. HR, hazard ratio; 95% CI, 95% CI. Cox proportional hazards regression. (**C**) Heatmap of FUBP1-regulated genes in LNCaP cells. Scr, scrambled control shRNA. (**D**) Protein levels of PDK1 and SLC7A11 after FUBP1 knockdown in prostate cancer cells. (**E**) Effect of FUBP1 on cell proliferation in various cell lines. (**F**) Effect of FUBP1 on xenograft growth. C4-2 cells with or without FUBP1 knockdown were used for a xenograft study in intact male NOD/SCID mice. The volume was calculated using the formula 0.5 × (length × width^2^). *n* = 5 for each group. (**G** and **H**) Effect of FUBP1 on xenograft weight. Scale bar: 10 mm. (**I**) Schema of potential PRMT5 methylation sites on FUBP1. (**J**) FLAG-FUBP1 methylation in HEK293T cells. AdOx, adenosine dialdehyde, a pan-inhibitor of arginine methyltransferases; MMA, monomethylarginine antibody; sDMA, symmetric dimethylarginine antibody; aDMA, asymmetric dimethylarginine antibody. (**K**) Methylation status of FUBP1 and related mutants in HEK293T cells. (**L**) Endogenous FUBP1 methylation in different cell lines. meFUBP1, a site-specific antibody for methylated FUBP1 at R359/R361/R363. **P < 0.01; 1-way ANOVA with Dunnett’s (T3) multiple-comparison test.

Clinical relevance of FUBP1 to tumor aggressiveness was observed in different cancer types, and the involvement of FUBP1 in prostate cancer was further determined using various databases (Supplemental Figure 1A; supplemental material available online with this article; https://doi.org/10.1172/JCI175023DS1). Higher FUBP1 expression is associated with earlier biochemical recurrence in prostate cancer patients (Figure 1, A and B) (36). Similar results were also found in the Chinese Prostate Cancer Genome and Epigenome Atlas (Supplemental Figure 1, B and C) (37, 38). Higher expression of FUBP1 was found in prostate cancer compared with the adjacent normal tissue (Supplemental Figure 1, D and E). To further determine the function of FUBP1 in prostate cancer, FUBP1 was knocked down in LNCaP cells for transcriptomic analysis. Multiple essential pathways for cell proliferation were altered, via transcriptional regulation and alterative splicing, after FUBP1 knockdown (Supplemental Figure 1, F–H). Previously reported oncogenic genes for prostate cancer, including SLC7A11 and pyruvate dehydrogenase kinase 1 (PDK1), were also suppressed after FUBP1 knockdown at both mRNA and protein levels in prostate cancer cells (Figure 1, C and D) (39–42). Consistent with previous reports, the knockdown of SLC7A11 and PDK1 suppressed cell growth in LNCaP and C4-2 cells (Supplemental Figure 1, I and J). However, c-Myc and P21, reported as FUBP1 target genes previously, were not regulated dramatically in our system, possibly because of different cell context (Figure 1D). Concurrently, cell proliferation was dramatically inhibited after FUBP1 knockdown in various prostate cancer cell lines but not in RWPE1, a normal prostate epithelial cell line (Figure 1E and Supplemental Figure 1, K and L). Stable cell lines derived from C4-2 cells, with or without FUBP1 depletion, were used for a xenograft study. FUBP1 knockdown strikingly impeded the development of prostate cancer in vivo, as indicated by xenograft weight and volume (Figure 1, F–H). Together, these data demonstrate the oncogenic effect of FUBP1 in prostate cancer.

To reveal the regulatory mechanisms of FUBP1 in prostate cancer, immunoprecipitation–mass spectrometry was performed. PRMT5, together with its regulator MEP50, was discovered to interact with FUBP1 (Supplemental Table 1). The PRMT5/MEP50 complex is well recognized for its arginine methylase activity and promotes the progression of multiple cancers, including prostate cancer (43, 44). An evolutionarily conserved PRMT5 modification sequence was also identified in FUBP1 (Figure 1I). Consequently, the methylation status of FUBP1 was investigated. Monomethylation and symmetric methylation, but not asymmetric methylation, were detected on FLAG-FUBP1 in HEK293T cells (Figure 1J). FLAG-FUBP1 methylation was obstructed by adenosine dialdehyde (AdOx), a pan-inhibitor of arginine methyltransferases (Figure 1J). Proteomics analysis of FUBP1 consistently revealed potential methylation at arginine 359/361/363 (3R) (Supplemental Figure 2A). Arginine-to-lysine mutations at these sites substantially curtailed FUBP1 methylation (Supplemental Figure 2, B and C). Notably, methylation was completely blocked in the FUBP1^3K^ mutant carrying R359/R361/R363K mutations (Figure 1K). A site-specific antibody (meFUBP1) was generated with a synthesized methylated peptide at R359/R361/R363 to recognize endogenous FUBP1 methylation. The specificity of the meFUBP1 antibody was validated via dot blot and peptide competition assay (Supplemental Figure 2, D and E). meFUBP1 recognized the wild-type FUBP1 but not FUBP1^3K^, and the signal diminished after AdOx treatment (Supplemental Figure 2, F and G). With this antibody, endogenous FUBP1 with R359/R361/R363 methylation was confirmed in different cell lines (Figure 1L). These data together demonstrate that R359/R361/R363 are the primary methylation sites of FUBP1.

### PRMT5-mediated FUBP1 methylation.

**Figure 2:**
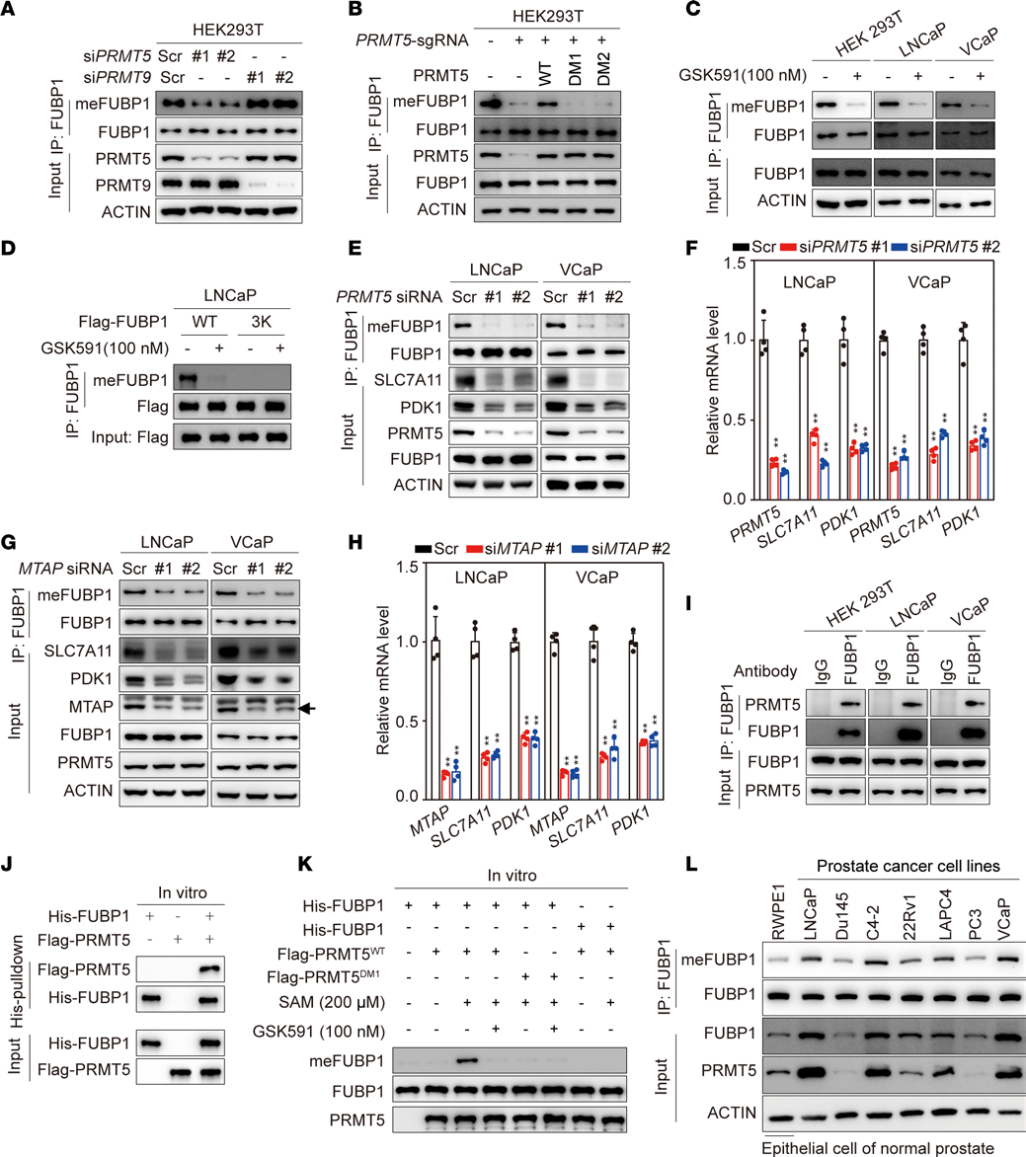
PRMT5-mediated FUBP1 methylation. (**A**) FUBP1 methylation status after PRMT5 or PRMT9 knockdown in HEK293T cells. Scr, scrambled control siRNA. (**B**) Re-expression of PRMT5 rescues FUBP1 methylation in HEK293T cells. DM1, a PRMT5 enzyme-dead mutant with G365A/R368A mutations; DM2, a PRMT5 enzyme-dead mutant with an E444Q mutation. (**C**) Status of FUBP1 methylation in different cell lines after treatment with the PRMT5 inhibitor GSK591, 100 nM. (**D**) PRMT5 inhibition specifically affects FUBP1 methylation at R359/R361/R363 in LNCaP cells. FUBP1 and FUBP1^3K^, a mutant with R359/R361/R363K mutations, were transiently expressed in LNCaP cells. (**E**) FUBP1 methylation in PRMT5-depleted prostate cancer cell lines. (**F**) Expression levels of FUBP1 target genes in LNCaP and VCaP cells with PRMT5 depletion. (**G**) FUBP1 methylation in MTAP-depleted prostate cancer cells. (**H**) Expression levels of FUBP1 target genes in MTAP-depleted LNCaP and VCaP cells. (**I**) Endogenous interaction of FUBP1 with PRMT5 in various cell lines. (**J**) Direct binding of PRMT5 to FUBP1 in vitro. His-FUBP1 was purified from *E. coli*, and FLAG-PRMT5 was enriched from HEK293T cell lysate. (**K**) In vitro methylation of FUBP1 by PRMT5. (**L**) Endogenous FUBP1 methylation and PRMT5 in different cell lines. Results are shown as mean ± SD. **P < 0.01; 1-way ANOVA with Dunnett’s (T3) multiple-comparison test.

Two arginine methyltransferases, PRMT5 and PRMT9, catalyze monomethylation and symmetric dimethylation on substrates (45). In HEK293T cells, the depletion of PRMT5, but not PRMT9, markedly repressed FUBP1 methylation (Figure 2A). CRISPR/Cas9–mediated knockout of PRMT5 substantially decreased in FUBP1 methylation, and the re-expression of wild-type PRMT5, but not the enzyme-dead mutants (DM1, PRMT5 with G365A/R368A mutations; DM2, PRMT5 with E444Q mutation), restored FUBP1 methylation (Figure 2B). GSK591, a PRMT5 inhibitor, also diminished FUBP1 methylation in different cell lines, without affecting FUBP1 protein levels (Figure 2C). The specificity of GSK591 suppressing FUBP1 methylation was further confirmed with the FUBP1^3K^ mutant (Figure 2D). In LNCaP and VCaP cells, PRMT5 knockdown had a limited effect on FUBP1 protein abundance but led to a marked decrease in FUBP1 methylation levels (Figure 2E). Interestingly, genes regulated by FUBP1, including *PDK1* and *SLC7A11*, were also inhibited after PRMT5 knockdown, indicating the importance of FUBP1 methylation for its oncogenic function (Figure 1C and Figure 2, E and F). Depletion of MTAP results in the accumulation of MTA, an endogenous PRMT5 inhibitor (34, 35). In our system, MTAP knockdown decreased FUBP1 methylation without affecting protein levels of PRMT5 and FUBP1 (Figure 2G). Consistently, the expression of PDK1 and SLC7A11 was reduced at both the mRNA and protein levels after MTAP knockdown (Figure 2, G and H). Together, these data demonstrate that PRMT5 regulates FUBP1 methylation, and this methylation is involved in the oncogenic effect of FUBP1.

The biochemistry of PRMT5-mediated FUBP1 methylation was further analyzed. Endogenous PRMT5 was pulled down by FUBP1 in different cell lines (Figure 2I). FLAG-PRMT5, immunopurified from HEK293T cells, binds to bacterially expressed His-FUBP1 in vitro (Figure 2J and Supplemental Figure 3). Wild-type FUBP1, but not the FUBP1^3K^ mutant, could be methylated by PRMT5 in vitro (Figure 2K). GSK591 blocked FUBP1 methylation, and the PRMT5 mutant with limited methylase activity failed to methylate FUBP1 in an in vitro methylation assay, indicating that PRMT5 directly affects FUBP1 methylation (Figure 2K). Endogenous PRMT5, FUBP1, and meFUBP1 were detected in all prostate cancer–related cell lines, highlighting that the PRMT5-mediated FUBP1 methylation is a universal event in prostate cancer (Figure 2L). The demethylases for FUBP1 were also explored. However, knockdown of these demethylases marginally affected FUBP1 methylation (Supplemental Figure 4). Together, these data demonstrate that PRMT5 binds to and directly methylates FUBP1.

### The effect of FUBP1 methylation on prostate cancer progression.

**Figure 3:**
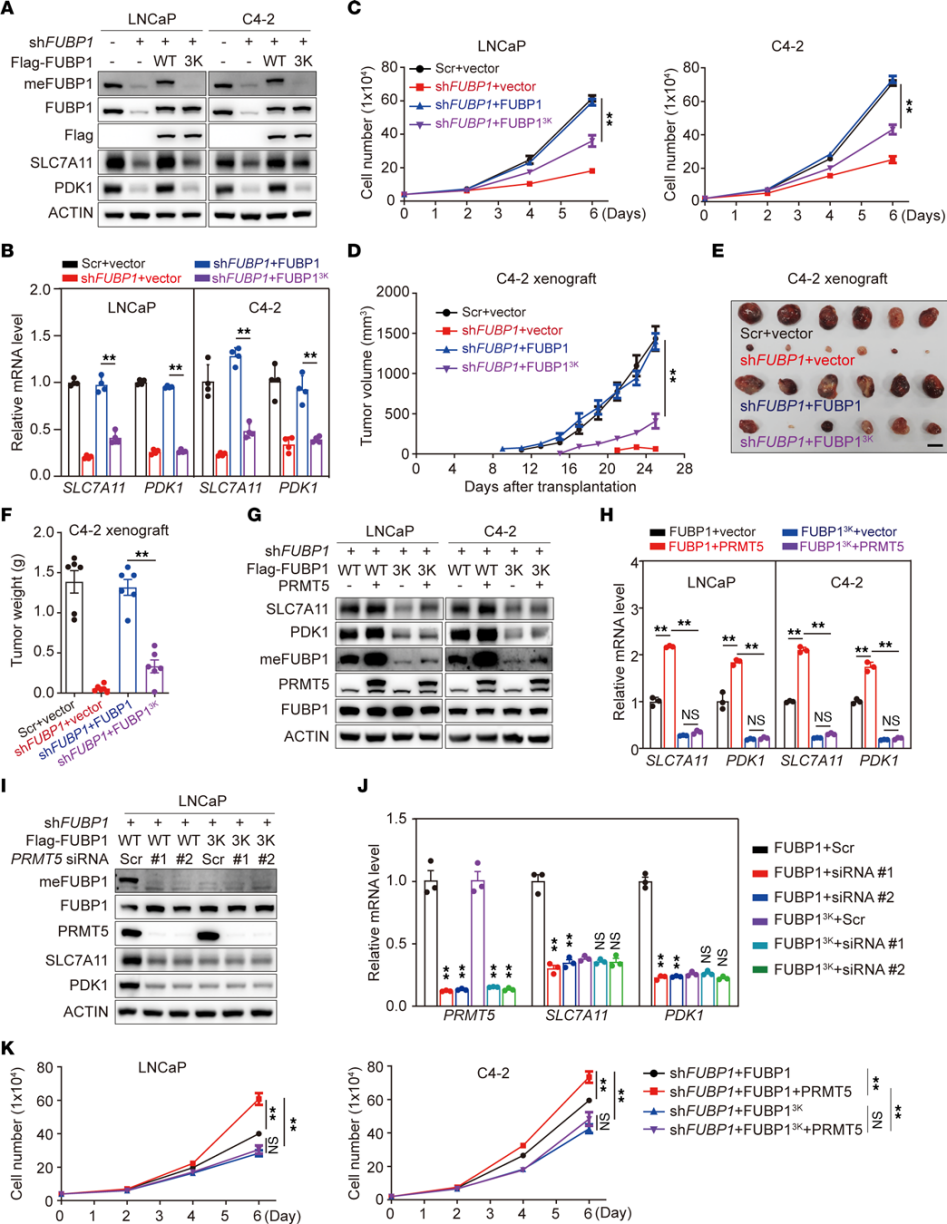
The effect of FUBP1 methylation on prostate cancer progression. (**A**) Protein abundance of FUBP1 and FUBP1^3K^ in stable cell lines. Stable cell lines were established in FUBP1-depleted LNCaP and C4-2 cells with wild-type FUBP1 or FUBP1^3K^ reintroduced at physiologically relevant levels. FUBP1^3K^, FUBP1 mutant with arginine-to-lysine mutations at R359/R361/R363. (**B**) Expression of FUBP1-regulated genes in stable cell lines. (**C**) Cell proliferation among the stable cell lines generated from LNCaP and C4-2 cells. (**D**) Effect of FUBP1 on xenograft growth. C4-2 stable cell lines were used for a xenograft study in intact NOD/SCID mice. *n* = 6 for each group. (**E** and **F**) The weight of xenografts derived from different C4-2 stable cell lines. Scale bar: 10 mm. (**G** and **H**) Effect of PRMT5 overexpression on FUBP1-regulated genes in various stable cell lines. One-way ANOVA. (**I** and **J**) Effect of PRMT5 knockdown on FUBP1-regulated genes in LNCaP stable cell lines. (**K**) Effect of PRMT5 on cell proliferation in different stable cell lines. **P < 0.01; 1-way ANOVA with Dunnett’s (T3) or Tukey’s multiple-comparison test.

To investigate the effect of FUBP1 methylation on prostate cancer, we established FUBP1-depleted LNCaP and C4-2 cell lines and reintroduced wild-type FUBP1 or FUBP1^3K^ at physiologically relevant levels (Figure 3A). PDK1 and SLC7A11 expression was reduced after FUBP1 depletion and rescued after the reintroduction of FUBP1, but not FUBP1^3K^ (Figure 3, A and B). Consistently, cell proliferation was suppressed in cells with FUBP1 knockdown. Reintroducing FUBP1, but not FUBP1^3K^, rescued cell growth in LNCaP and C4-2 stable cells (Figure 3C). A xenograft study was conducted using these stable cell lines. FUBP1 depletion curtailed xenograft growth, whereas reintroducing FUBP1 robustly accelerated xenograft growth, as determined by tumor weight and volume. Unlike FUBP1, FUBP1^3K^ failed to rescue xenograft growth (Figure 3, D–F). Together, these data demonstrate that FUBP1 methylation promotes the development of prostate cancer.

To further illustrate the oncogenic effect of FUBP1 methylation, we overexpressed PRMT5 in FUBP1-depleted stable cell lines in which FUBP1 or FUBP1^3K^ was reintroduced. PRMT5 enhanced the expression of PDK1 and SLC7A11 in cells with reintroduced FUBP1 but not with FUBP1^3K^ (Figure 3, G and H). In contrast, PRMT5 knockdown in these cells resulted in suppressed meFUBP1 levels and expression of SLC7A11 and PDK1 only in LNCaP cells with reintroduced FUBP1 (Figure 3, I and J). Consistently, PRMT5 promoted proliferation in cells with FUBP1 reintroduction, but displayed only a marginal effect in those with FUBP1^3K^ reintroduction (Figure 3K). Together, these data demonstrate that PRMT5-mediated FUBP1 methylation is important for FUBP1’s oncogenic effects.

### Deficiency in FUBP1 methylation delays prostate cancer progression in vivo.

**Figure 4:**
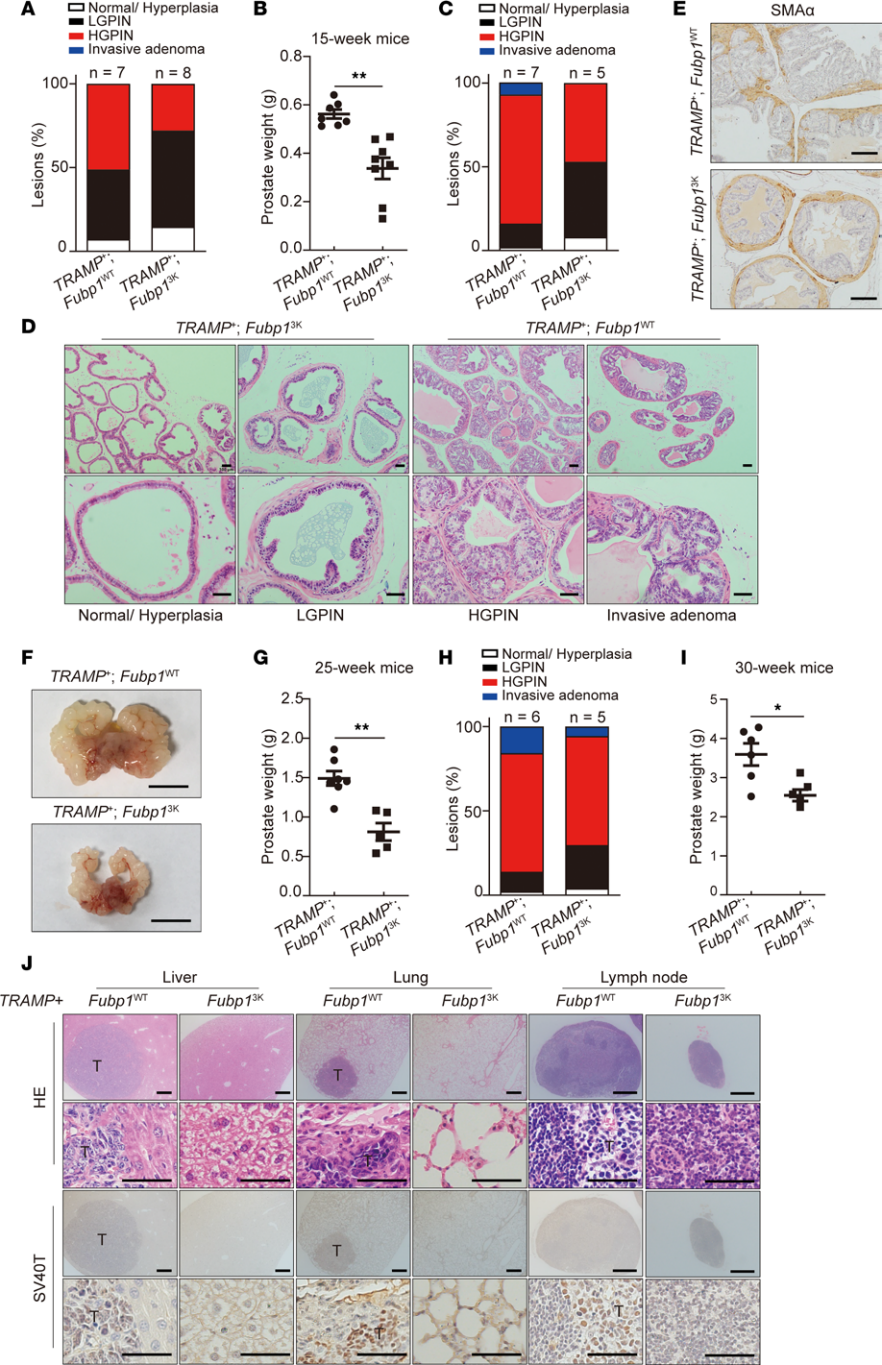
Deficiency in FUBP1 methylation delays prostate cancer progression in vivo. (**A**) Quantification of mouse prostate tumor histological grade in *TRAMP^+/–^* *Fubp1^WT/WT^* (*TRAMP^+^* *Fubp1^WT^*) and *TRAMP^+/–^* *Fubp1^3K/3K^* (*TRAMP^+^* *Fubp1^3K^*) mice at age 15 weeks. “Lesions (%)” represents the ratio of a specific histological grade in all samples. (**B**) Prostate weight in *TRAMP^+^* *Fubp1^WT^* and *TRAMP^+^* *Fubp1^3K^* mice at age 15 weeks. (**C**) Quantification of mouse prostate tumor histological grade in *TRAMP^+^* *Fubp1^WT^* and *TRAMP^+^* *Fubp1^3K^* mice at age 25 weeks. (**D**) Representative H&E staining in *TRAMP^+^* *Fubp1^WT^* and *TRAMP^+^* *Fubp1^3K^* mice at age 25 weeks. Scale bars: 100 μm (top) and 50 μm (bottom). (**E**) IHC for SMAα in ventral prostate sections from mice at age 25 weeks. Scale bars: 100 μm. (**F** and **G**) Gross photographs and quantification of prostate weight in *TRAMP^+^* *Fubp1^WT^* and *TRAMP^+^* *Fubp1^3K^* mice at age 25 weeks. Scale bars: 1 cm. (**H**) Quantification of histological grade of mouse prostate in *TRAMP^+^* *Fubp1^WT^* and *TRAMP^+^* *Fubp1^3K^* mice at age 30 weeks. (**I**) Quantification of prostate weight in *TRAMP^+^* *Fubp1^WT^* and *TRAMP^+^* *Fubp1^3K^* mice at age 30 weeks. (**J**) Representative H&E staining for indicated tissues in *TRAMP^+^* *Fubp1^WT^* and *TRAMP^+^* *Fubp1^3K^* mice at age 30 weeks. Scale bars: 100 μm (rows 1 and 3) and 200 μm (rows 2 and 4). *P < 0.05, **P < 0.01; 2-tailed Student’s *t* test.

To investigate the pathological effects of FUBP1 methylation in vivo, a genetic knockin mouse model carrying a homozygous Fubp1 R354/R356/R358K mutant (*Fubp1^3K^*) was generated (Supplemental Figure 5, A and B), and then crossed with the transgenic adenocarcinoma mouse prostate (TRAMP) model (46, 47). The mice were euthanized at the age of 15, 25, or 30 weeks for histopathological analysis to investigate the effect of Fubp1 methylation in prostate cancer at different stages. Compared with *TRAMP^+/–^* *Fubp1^WT/WT^* (*TRAMP^+^* *Fubp1^WT^*) mice, 15-week-old *TRAMP^+/–^* *Fubp1^3K/3K^* (*TRAMP^+^* *Fubp1^3K^*) mice showed delayed prostate cancer initiation, as indicated by lower penetrance of high-grade prostatic intraepithelial neoplasia (HGPIN) and low-grade prostatic intraepithelial neoplasia (LGPIN) (Figure 4A). Consistently, prostate tissue weight in 15-week-old *TRAMP^+^* *Fubp1^3K^* mice was lower than that in *TRAMP^+^* *Fubp1^WT^* mice (Figure 4B). Invasive adenoma, as indicated by α–smooth muscle actin (SMAα) staining, was observed in approximately 8% of immunohistochemistry (IHC) samples from *TRAMP^+^* *Fubp1^WT^* mice, but not in samples from 25-week-old *TRAMP^+^* *Fubp1^3K^* mice (Figure 4, C–E, and Supplemental Figure 5C). Prostate tissue weight was also lower in 25-week-old *TRAMP^+^* *Fubp1^3K^* mice (Figure 4, F and G). A greater number of invasive adenocarcinomas was found in tissue samples from 30-week-old mice. Still, *TRAMP^+^* *Fubp1^3K^* mice had fewer metastatic lesions and lower prostate tissue weight (Figure 4, H and I). Metastatic adenocarcinoma in the liver, lung, and lymph nodes was present in *TRAMP^+^* *Fubp1^WT^* mice, but barely found in *TRAMP^+^* *Fubp1^3K^* mice (Figure 4J). The abundance of Fubp1 methylation and its downstream effectors was further determined. Fubp1 abundance in *TRAMP^+^* *Fubp1^3K^* was comparable to that in *TRAMP^+^* *Fubp1^WT^* mice. However, meFubp1 was absent only in the prostate tissues and metastasis sites from *TRAMP^+^* *Fubp1^3K^* mice (Supplemental Figure 5D). Consistently, lower expression levels of Slc7a11 and Pdk1 were found in the prostate tissues and liver from *TRAMP^+^* *Fubp1^3K^* mice (Supplemental Figure 5D). Together, these data demonstrate that Fubp1 methylation is essential for disease progression in TRAMP mice.

**Figure 5:**
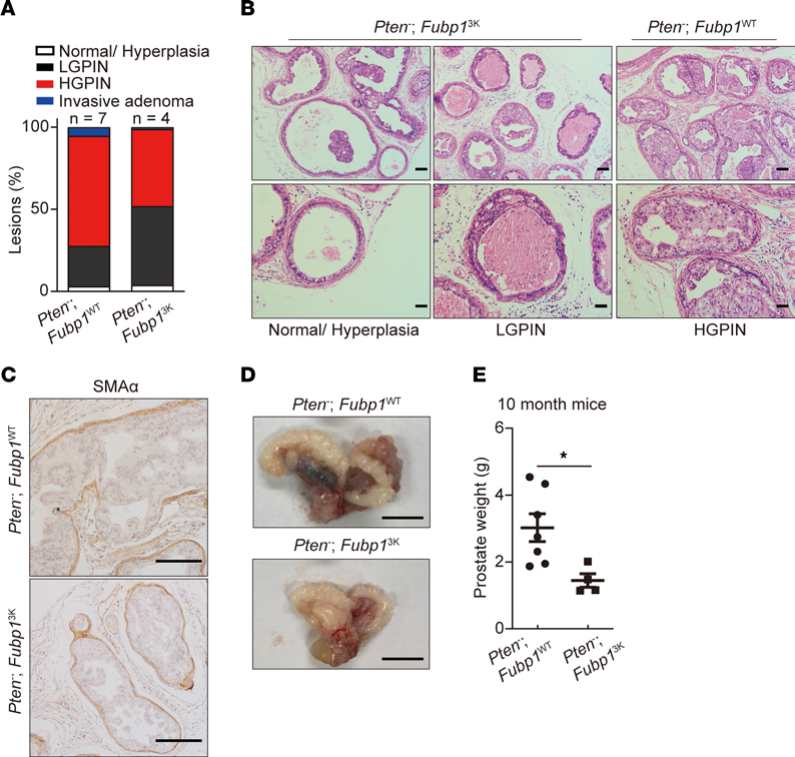
FUBP1 methylation promotes prostate cancer progression in vivo. (**A**) Quantification of mouse prostate tumor histological grade in *Probasin*-*Cre^+/–^ Pten^fl/fl^* *Fubp1* (*Pten^–^* *Fubp1^WT^*) and *Probasin*-*Cre^+/–^ Pten^fl/fl^* *Fubp1^3K/3K^* (*Pten*^–^ *Fubp1^3K^*) mice at age 10 months. (**B**) Representative H&E staining in *Pten^–^* *Fubp1^WT^* and *Pten^–^* *Fubp1^3K^* mice. Scale bars: 100 μm (top) and 50 μm (bottom). (**C**) IHC for SMAα in *Pten^–^* *Fubp1^WT^* mice. Scale bars: 100 μm. (**D** and **E**) Gross photographs and quantification of prostate weight in *Pten^–^* *Fubp1^WT^* and *Pten^–^* *Fubp1^3K^* mice at age 10 months. Scale bars: 1 cm. *P < 0.05; 2-tailed Student’s *t* test.

The function of FUBP1 methylation in prostate cancer was also investigated in a mouse model with prostate-specific *Probasin*-mediated *Pten* deletion (48, 49). Ten-month old *Probasin*-*Cre^+/–^ Pten^fl/fl^* *Fubp1^3K/3K^* (*Pten*^–^ *Fubp1^3K^*) mice had less HGPIN and fewer invasive adenocarcinomas (Figure 5, A–C, and Supplemental Figure 5C). Lower-weight prostate tissues were found in *Pten*^–^ *Fubp1^3K^* mice (Figure 5, D and E). Together, these results support the oncogenic effect of FUBP1 methylation in prostate cancer.

### Involvement of BRD4 in PRMT5-mediated FUBP1 methylation.

**Figure 6:**
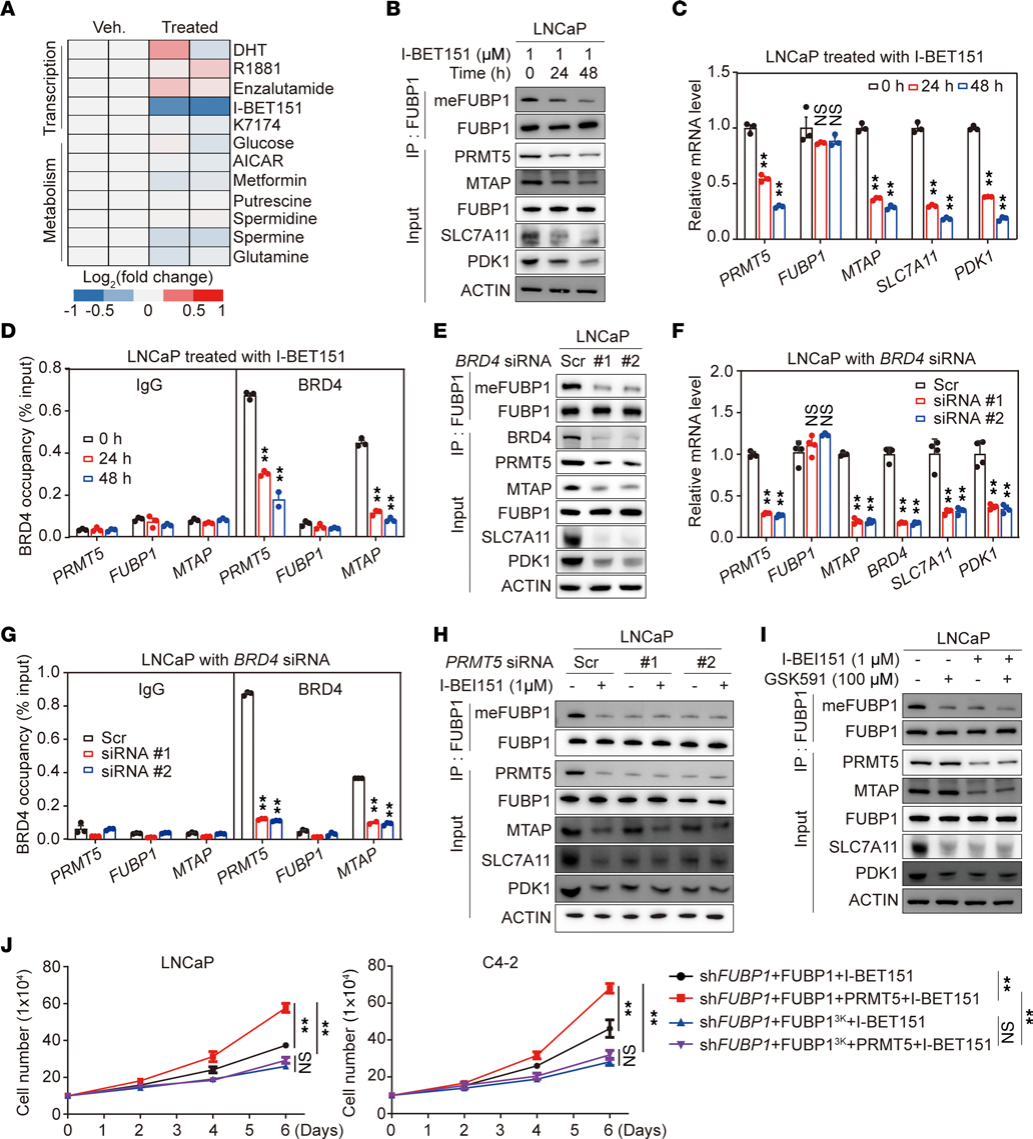
Involvement of BRD4 in PRMT5-mediated FUBP1 methylation. (**A**) Effect of different small molecules on FUBP1 methylation in LNCaP cells. Cells were treated with a variety of small molecules related to transcriptional or metabolic regulation. (**B**) FUBP1 methylation after treatment with the BRD4 inhibitor I-BET151 in LNCaP cells. (**C**) Effect of 1 μM I-BET151 on gene expression in LNCaP cells. (**D**) Enrichment of BRD4 on different gene promoters after I-BET151 treatment. (**E** and **F**) Effect of BRD4 on FUBP1 methylation and its function in LNCaP cells. (**G**) BRD4 enrichment on different gene promoters in LNCaP cells. (**H** and **I**) Involvement of PRMT5 in BRD4-regulated FUBP1 methylation. LNCaP cells were treated with I-BET151 with or without PRMT5 siRNA or a PRMT5 inhibitor. (**J**) The effect of PRMT5 on cell proliferation after I-BET151 treatment. *P < 0.05, **P < 0.01; 1-way ANOVA with Dunnett’s (T3) or Tukey’s multiple-comparison test.

Considering the importance of FUBP1 methylation in prostate cancer, upstream signaling regulating PRMT5-mediated FUBP1 methylation was assessed. LNCaP cells were treated with a panel of molecules associated with transcriptional or metabolic regulation, and endogenous FUBP1 methylation was detected. I-BET151, a bromodomain-containing protein 4 (BRD4) inhibitor, suppressed FUBP1 methylation substantially (Figure 6A). Consistent with the suppressed FUBP1 methylation, protein levels of PDK1 and SLC7A11 were decreased after I-BET151 treatment in LNCaP and VCaP cells (Figure 6B and Supplemental Figure 6A). The protein levels of PRMT5 and MTAP, but not FUBP1, were markedly reduced after I-BET151 treatment (Figure 6B and Supplemental Figure 6A). Similarly, I-BET151 suppressed mRNA levels of PRMT5 and MTAP, but not that of FUBP1, indicating that PRMT5 and MTAP are direct target genes of BRD4 (Figure 6C and Supplemental Figure 6B). Results of chromatin immunoprecipitation (ChIP) confirmed the direct binding of BRD4 to the promoters of PRMT5 and MTAP, which was abrogated by I-BET151 (Figure 6D and Supplemental Figure 6C). Marginal BRD4 binding was detected on the FUBP1 promoter (Figure 6D and Supplemental Figure 6C). Consistently, BRD4 depletion led to a reduction of PRMT5 and MTAP mRNA and protein (Figure 6, E and F, and Supplemental Figure 6, D and E). FUBP1 methylation was suppressed, although FUBP1 mRNA and protein abundance was not affected by BRD4 depletion. The expression of PDK1 and SLC7A11 also decreased after BRD4 depletion, supporting the involvement of BRD4 in PRMT5-mediated FUBP1 methylation (Figure 6, E and F, and Supplemental Figure 6, D and E). BRD4 depletion also prevented the enrichment of endogenous BRD4 on the promoters of PRMT5 and MTAP (Figure 6G and Supplemental Figure 6F). However, PRMT5 depletion or functional inhibition with GSK591 abolished the effect of BRD4 inhibitor on FUBP1 methylation (Figure 6, H and I). PRMT5 overexpression countered the effect of the BRD4 inhibitor and accelerated proliferation in cells with stable reintroduction of FUBP1, but not FUBP1^3K^ (Figure 6J). Together, these data demonstrate that BRD4 transcriptionally regulates PRMT5 and MTAP to affect FUBP1 methylation.

### Clinical relevance of FUBP1 methylation in prostate cancer.

**Figure 7:**
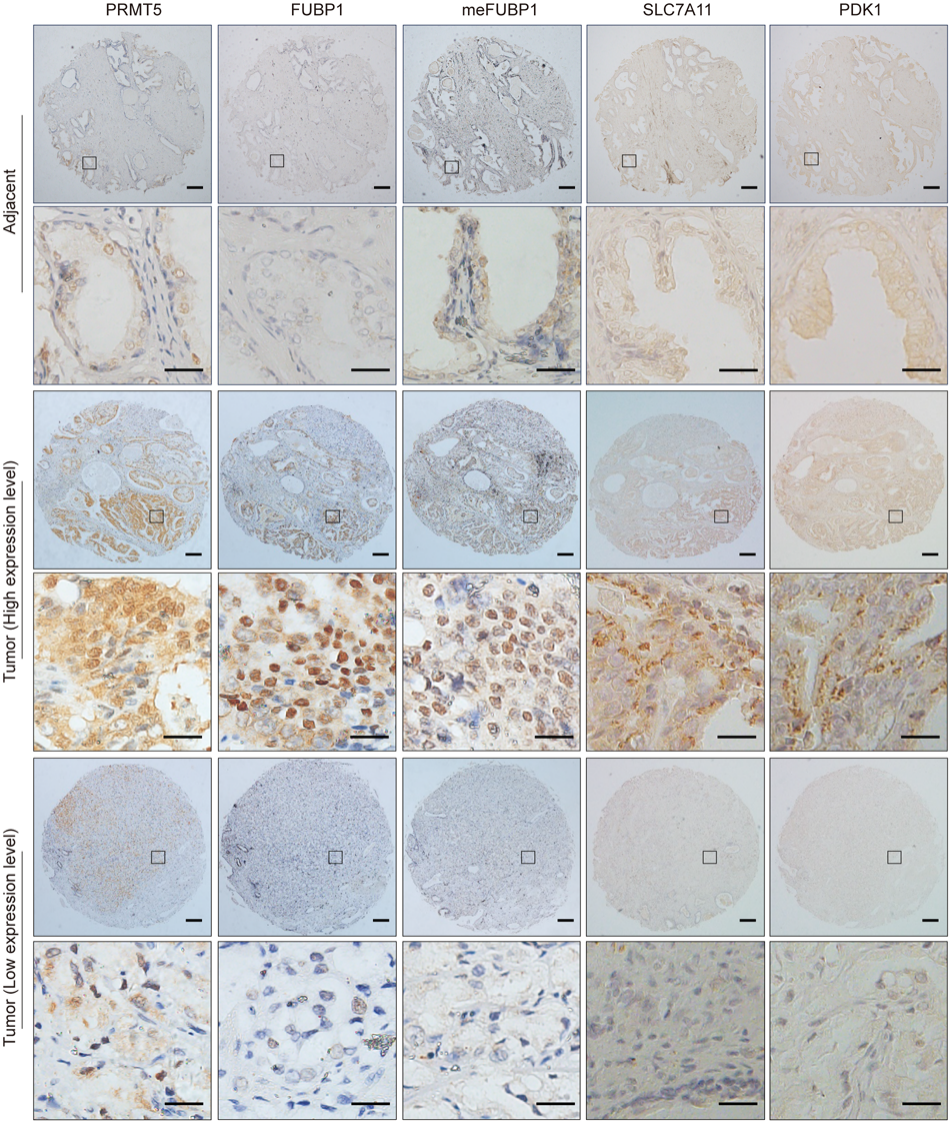
Representative IHC results for a patient prostate tissue microarray. Scale bars: 200 μm (top) and 100 μm (bottom), respectively.

**Figure 8:**
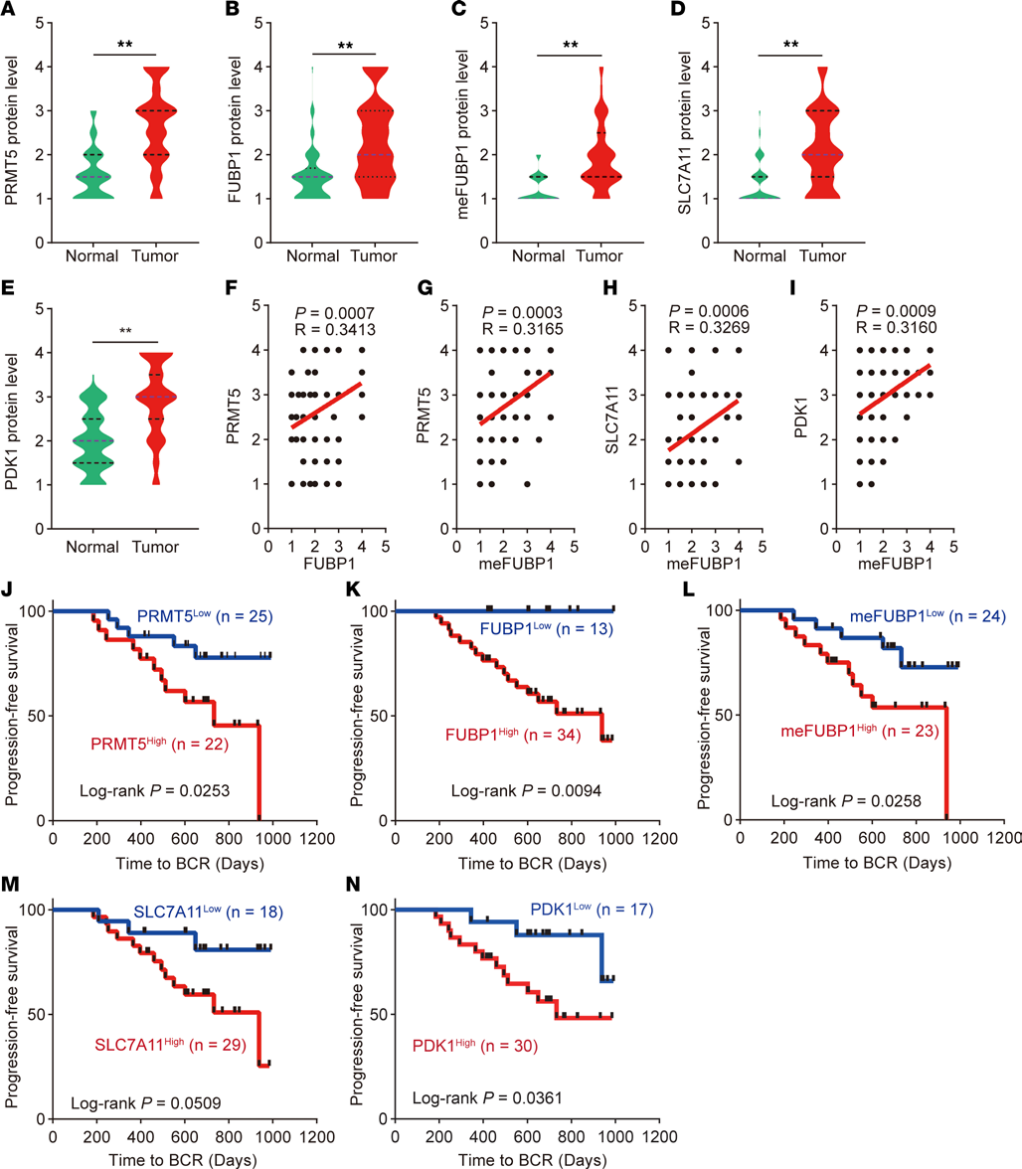
Clinical relevance of FUBP1 methylation in prostate cancer. (**A**–**E**) Protein levels of PRMT5, FUBP1, methylated FUBP1, PDK1, and SLC7A11 in the tissue microarray (TMA) (*n* = 107). Median and quartiles are shown as blue and black dashed lines, respectively. **P < 0.01; 2-tailed Student’s *t* test. (**F** and **G**) Correlation of PRMT5 with FUBP1 and methylated FUBP1 at the protein level as analyzed in the TMA (*n* = 107). (**H** and **I**) Correlations of methylated FUBP1 with PDK1 and SLC7A11 at the protein level analyzed in the TMA (*n* = 107). (**J**–**N**) Effects of the PRMT5-FUBP1-PDK1/SLC7A11 axis on patient biochemical recurrence (BCR). Log-rank test.

To investigate the clinical relevance of the BRD4-PRMT5-FUBP1-PDK1/SLC7A11 axis in prostate cancer, a tissue microarray composed of 107 paired prostate cancer and adjacent non-cancerous tissue samples from patients receiving prostatectomy was created for IHC assay of PRMT5, FUBP1, methylated FUBP1, PDK1, and SLC7A11 (Figure 7) (7). The specificity of meFUBP1 to detect methylated FUBP1 for IHC was validated, and the signals detected by meFUBP1 were specifically blocked by methylated peptides, but not unmethylated peptides (Supplemental Figure 7, A and B). Protein abundance (4 levels) based on IHC density was scored (Supplemental Figure 7C). Increased levels of PRMT5 and FUBP1 were detected in tumor tissues compared with adjacent tissues (Figure 8, A and B). Consistently, methylated FUBP1, PDK1, and SLC7A11 were also more robustly expressed in tumor tissues (Figure 8, C–E). Further analysis indicated that PRMT5 levels positively correlated with the abundance of FUBP1 and methylated FUBP1 (Figure 8, F and G). Moreover, FUBP1 methylation positively correlated with the expression of the FUBP1 target genes PDK1 and SLC7A11, supporting the importance of arginine methylation for FUBP1’s oncogenic effects (Figure 8, H and I). Together, these data demonstrate that the PRMT5-FUBP1- PDK1/SLC7A11 axis is enhanced in tumor cells.

To further investigate the effect of FUBP1 on disease progression, correlation of the PRMT5-FUBP1-PDK1/SLC7A11 axis with treatment response was analyzed. Patients with high levels of PRMT5, FUBP1, and FUBP1 methylation had earlier biochemical recurrence (Figure 8, J–L). The FUBP1 downstream genes PDK1 and SLC7A11 were also identified as risk factors for treatment resistance (Figure 8, M and N). Together, these results demonstrate that FUBP1 and its methylation facilitate disease progression in patients with prostate cancer.

### Targeting FUBP1 methylation with a nanocomplex-delivered peptide.

**Figure 9:**
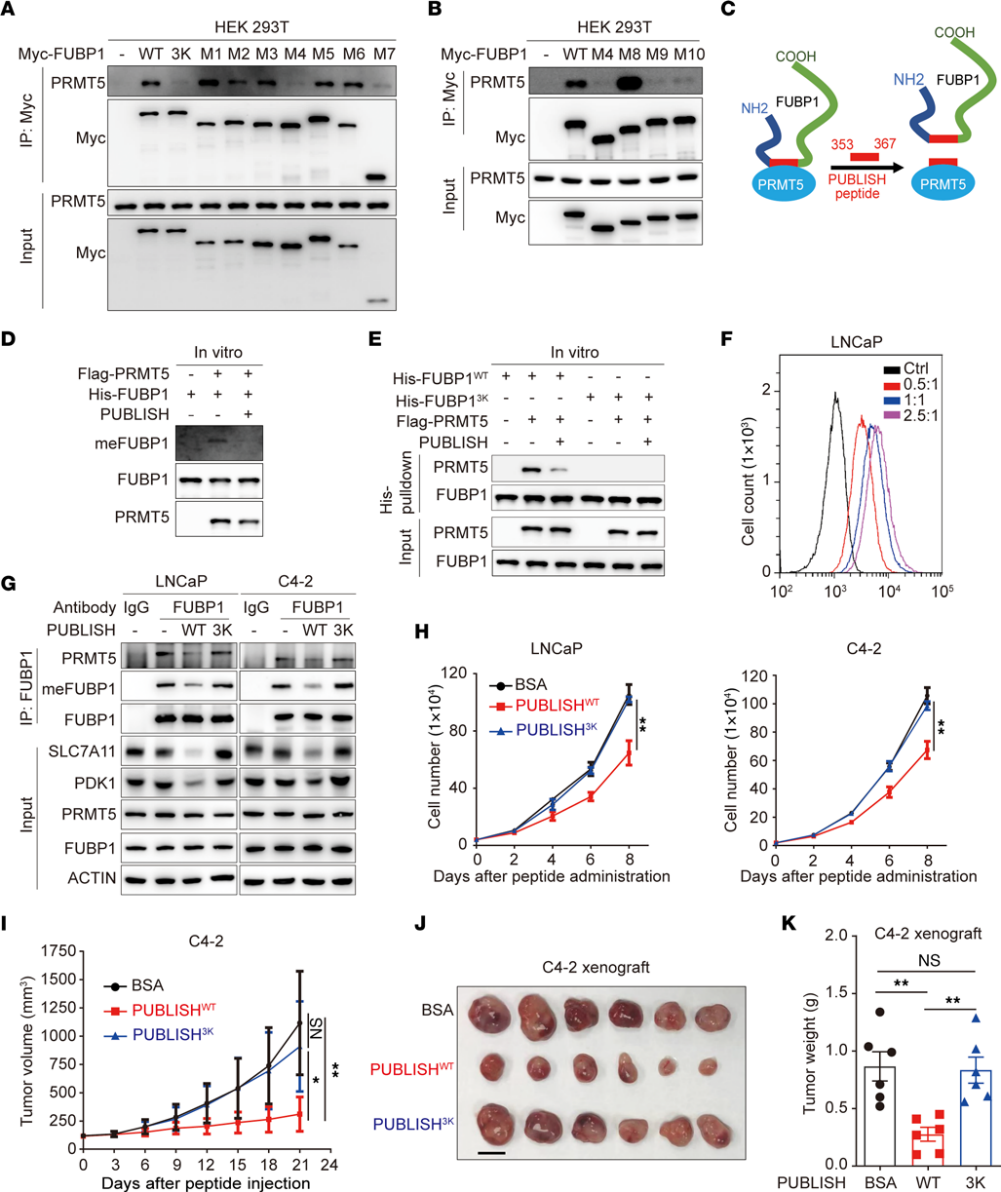
Targeting FUBP1 methylation with a nanocomplex-delivered peptide. (**A** and **B**) Affinity of PRMT5 for FUBP1 and related truncations (M1–M10). (**C**) Schema for the PUBLISH competitive peptide function. PUBLISH peptide, PRMT5-mediated FUBP1 methylation abolishing peptide. (**D**) Effect of the PUBLISH peptide on the binding of FUBP1 to PRMT5 in vitro. His-FUBP1 was purified from *E. coli*, and FLAG-PRMT5 was enriched from HEK293T cell lysate. (**E**) Effect of the PUBLISH peptide on FUBP1 methylation in vitro. (**F**) Efficiency of nanocomplexes for peptide intracellular delivery. A fluorescein isothiocyanate–conjugated PUBLISH peptide (353–367 aa) was synthesized and mixed with BPAE at different BPAE/peptide weight ratios to test the delivery efficiency. (**G**) Intracellular effect of the PUBLISH peptide on methylation and gene expression. FUBP1 methylation status and the expression of FUBP1-regulated genes were determined with or without peptide delivery in prostate cancer cell lines. PUBLISH^WT^, PUBLISH peptide (353–367 aa); PUBLISH^3K^, PUBLISH peptide with R359/R361/R363K mutations. (**H**) Effect of the PUBLISH peptide on cell proliferation in prostate cancer cell lines. (**I**) Effect of the PUBLISH peptide on xenograft growth. C4-2 cells were used for xenograft assay in intact NOD/SCID mice. Nanocomplex-delivered peptide (WT or 3K) (5 μg/mL) was intraperitoneally injected every 2 days for 3 weeks after the xenograft reached approximately 150 mm^3^. The volume was calculated using the formula 0.5 × (length × width^2^). For each group of mice, *n* = 6. (**J** and **K**) Effect of the PUBLISH peptide on xenograft weight. Scale bar: 10 mm. Results are shown as mean ± SD. *P < 0.05, **P < 0.01; 1-way ANOVA with Tukey’s multiple-comparison test.

Given the importance of FUBP1 methylation to oncogenic progression, strategies to interfere with FUBP1 methylation might provide potential therapeutic approaches for cancer management. To generate a competitive peptide capable of blocking the interaction of FUBP1 with PRMT5, the minimal interaction domain in FUBP1 was determined. Myc-tagged FUBP1 truncations (M1–M10) with a C-terminal nuclear localization sequence were generated to bind to PRMT5 in HEK293T cells (Supplemental Figure 8, A and B). The region spanning amino acids 353–367 in FUBP1, containing the PRMT5-modified R359/R361/R363 amino acids, was found to be the minimal domain mediating the interaction of FUBP1 with PRMT5 (Figure 9, A and B). This minimal binding region was synthesized as a competitive peptide, named PRMT5-mediated FUBP1 methylation abolishing (PUBLISH) peptide, to interfere with the formation of the PRMT5/FUBP1 complex and prevent FUBP1 methylation (Figure 9C). The PUBLISH peptide successfully disrupted the interaction of PRMT5 and FUBP1 in vitro (Figure 9D). PUBLISH also inhibited PRMT5-mediated FUBP1 methylation in vitro (Figure 9E). Together, these data indicate that the competitive peptide can prevent FUBP1 methylation.

To test the intracellular effect of the PUBLISH peptide, branched poly(β-amino ester) (BPAE), a branched cationic polymer we previously developed for cytosolic protein/peptide delivery, was used to deliver the peptide into prostate cancer cells (Supplemental Figure 9A) (50). BPAE was assembled with the peptide via N-B coordination, hydrophobic, and electrostatic interactions, forming nanocomplexes (NCs) of about 120.5 nm and a positive ζ potential of 15.2 mV at a BPAE/peptide weight ratio of 2:1 (Supplemental Figure 9B). The NC efficiently delivered fluorescein isothiocyanate–conjugated PUBLISH peptide (FITC-PUBLISH) into LNCaP cells (Figure 9F). A mutated peptide (PUBLISH^3K^), having the same sequence as the PUBLISH peptide but with methylation site mutations (R359/R361/R363K), was also synthesized. The PUBLISH peptide, but not the PUBLISH^3K^, successfully suppressed the interaction of FUBP1 with PRMT5 in LNCaP and C4-2 cells (Figure 9G). Consistently, FUBP1 methylation was inhibited and protein levels of PDK1 and SLC7A11 were reduced after PUBLISH peptide delivery (Figure 9G). The PUBLISH peptide, but not PUBLISH^3K^, also suppressed cell proliferation in LNCaP and C4-2 cells (Figure 9H). FUBP1-IN-1, a previously reported FUBP1 inhibitor, was used to treat LNCaP cells (51, 52). The PUBLISH peptide suppressed cell proliferation at the same level as FUBP1-IN-1, indicating the essential role of FUBP1 methylation for its oncogenic effect (Supplemental Figure 9C). The in vivo antitumor function of this competitive peptide was further tested in a xenograft model using C4-2 cells. Considering that positively charged NCs are unstable in vivo, BPAE/peptide NCs were surface-decorated with hyaluronic acid (HA), a polyanionic material, yielding negatively charged HA/BPAE/peptide NCs for in vivo application. At an HA/BPAE weight ratio of 10, HA/BPAE/peptide NCs had the smallest particle size of about 137.6 nm and a negative ζ potential of –16.1 mV (Supplemental Figure 9, D and E). Consistently, HA/BPAE/peptide NCs at this optimal ratio showed the highest internalization in LNCaP cells (Supplemental Figure 9F). The PUBLISH peptide substantially prevented xenograft growth, whereas the PUBLISH^3K^ peptide had a limited effect (Figure 9, I–K). Together, these data demonstrate that this competitive peptide effectively prevents FUBP1 methylation and inhibits tumor growth.

## Discussion

Cancer remains one of the leading causes of death across the world. Mortality rates have declined for lung, colorectal, and breast cancers. However, this trend has not occurred in prostate cancer. Advanced prostate cancer inevitably becomes heterogeneous and resistant to hormone-related therapies. Therefore, innovative targets beyond the androgen-AR axis are required to achieve substantial progress against prostate cancer. Here, by investigating the function and modification of FUBP1 in prostate cancer, we found that the BRD4-PRMT5-FUBP1 axis is essential for prostate cancer progression. We also determined that nanocomplex (NC) delivery of a competitive peptide to block interaction of FUBP1 with PRMT5 is a potential therapeutic strategy to treat prostate cancer by preventing PRMT5-mediated FUBP1 methylation.

FUBP1 is essential for tissue homeostasis owing to its multiple functions, and FUBP1 deficiency is embryonically lethal (53). Dysregulation of FUBP1 has been found in different cancer types. FUBP1 deletion is associated with oligodendroglioma and breast cancer, while FUBP1 also promotes the mobility of lung cancer (22, 26, 54). Such findings demonstrate that FUBP1 function is context specific and is regulated subtly. Here our results demonstrate that FUBP1 promotes the development of prostate cancer via regulating oncogenic genes, including PDK1 and SLC7A11 (39–42). Alterations in RNA splicing were also observed in FUBP1-knockout cell lines, indicating that multiple mechanisms are involved in the oncogenic function of FUBP1 in prostate cancer. However, c-Myc and P21, reported previously as FUBP1 target genes, were not consistently and robustly regulated after FUBP1 knockdown. The genomic aberration of prostate cancer cells, with a varied chromatin 3D structure, may lead to dysregulation of c-Myc and P21 by FUBP1. FUBP1 methylation is essential for its oncogenic effect, and the correlation of FUBP1 methylation with prostate cancer progression was validated in different mouse models and clinical specimens. Thus, FUBP1 methylation could be a biomarker for prostate cancer progression. Also, the context-specific function and regulation of FUBP1 should be further investigated in other cancer types.

PRMT5 has been reported to promote prostate cancer development via multiple mechanisms (43, 55). Here we found that PRMT5 methylates FUBP1 at R359/R361/R363, which was important for the oncogenic effect of FUBP1 in prostate cancer. PRMT5 inhibitors have been developed and tested in clinical trials for cancer treatment (ClinicalTrials.gov NCT03573310 and NCT05094336, among others) (33, 56–58). The expression of PRMT5, FUBP1, and meFUBP1 seems to be correlated in prostate cancer cell lines (Figure 2L), supporting a potential biomarker role of meFUBP1 for PRMT5 activity. meFUBP1 might be used in PRMT5 inhibitor–related trials for patient selection. Given the variety of PRMT5 substrates, inhibitors directly targeting PRMT5 might have broad and nonspecific effects in cancer cells and even normal cells. Approaches that interfere with the function of PRMT5 on a specific substrate may offer efficacy with limited toxicity. A competitive peptide (PUBLISH), originated from the minimal interaction domain in FUBP1 mediating PRMT5-FUBP1 interaction, effectively blocked PRMT5-mediated FUBP1 methylation and successfully suppressed the development of prostate cancer. This supports the concept that precision intervention on PRMT5 function may be used to treat prostate cancer. Also, the competitive peptide encompasses the conserved PRMT5 modification motif in FUBP1, indicating a potentially universal strategy to design specific PRMT5 inhibitors: peptides containing a conserved PRMT5 modification motif plus proximal amino acid residues from the substrate might function as PRMT5 inhibitors with enhanced specificity. The PRMT5-mediated FUBP1 methylation is independent of AR signaling; thus, the PUBLISH peptide may be used together with hormone therapy to achieve better clinical efficacy.

To improve the targeting efficiency of our delivery system, BPAE/peptide NCs are surface-decorated with hyaluronic acid (HA), a polyanionic material with excellent biocompatibility. HA coating shields the positive surface charges of NCs, thus prolonging their blood circulatory time and facilitating passive tumor targeting through the enhanced permeation and retention effect. Moreover, HA also serves as a tumor-targeting ligand that recognizes CD44 overexpression on various tumor cell membranes such as prostate cancer, breast cancer, and non–small cell lung cancer, and thus HA-coated NCs can also actively target to tumors after systemic administration.

As PRMT5-mediated FUBP1 methylation is not limited to prostate cancer, the clinical application of this competitive peptide may extend to other cancers, and HA/BPAE/peptide NCs could be used to treat various cancers with reduced systemic side effects and improved therapeutic efficacy. Other tumor targeting strategies could be adopted for PUBLISH peptide delivery as well, such as the ATTACK strategy we developed previously (59). Notably, the nano-properties of the delivery system need to be tailored for different tumor types and administration routes. For instance, nano-vehicles with robust tumor penetration capabilities are demanded for pancreatic cancer, which has dense extracellular matrix; mucus-penetrating nano-vehicles would be desired for the bladder infusion delivery. We will explore such other strategies in future studies.

BRD4 is an upstream regulator of PRMT5-mediated FUBP1 methylation and promotes FUBP1 methylation by directly facilitating PRMT5 transcription. BRD4 also indirectly regulates PRMT5 activity by transcriptionally regulating MTAP. MTAP converts MTA, an endogenous PRMT5 inhibitor, to methionine and then to SAM, the methyl donor for PRMT5 in the methionine salvage pathway. BRD4 is well known as an AR coactivator in prostate cancer (16). BRD4 also facilitates the expression of MYC and AR-v7 (60). Targeting BRD4 via BET inhibitors or proteolysis-targeting chimera (PROTAC) degraders suppresses prostate cancer development, and clinical evaluation of BRD4 inhibitors is under way in patients with castration-resistant prostate cancer (NCT02711956, NCT03150056) (61–63). Our results provide an additional mechanism to support BRD4 as a potential therapeutic target for prostate cancer. PRMT5-mediated FUBP1 methylation might also be a marker for potential resistance to I-BET151, which could be used to better tailor treatment to patients in the clinic.

In summary, we report that the BRD4-PRMT5/MTAP axis regulates FUBP1 methylation and is essential for prostate cancer progression. A competitive peptide blocking FUBP1 methylation effectively suppresses prostate cancer development. The oncogenic effect of PRMT5-mediated FUBP1 methylation is unlikely to be limited to prostate cancer. Our design of the competitive peptide provides a feasible strategy to develop additional specific functional inhibitors for PRMT5 or even other methylases, and the methylation status of substrates may provide insights for patient selection.

## Methods

### Sex as a biological variable.

Our study exclusively examined male mice and patients because we investigated prostate cancer in humans.

### Cell lines and materials.

HEK293T, LNCaP, and C4-2 cells were purchased from the American Type Culture Collection and cultured in DMEM (HEK293T) and RPMI 1640 (LNCaP and C4-2) with 10% FBS (ExCellBio). VCaP cells were provided by Jun Qin (Shanghai Institute of Nutrition and Health, Shanghai, China) and cultured in DMEM with 10% FBS (ExCellBio, China) and 1 mM sodium pyruvate (Gibco, Thermo Fisher Scientific). Cell lines were authenticated by Hybribio and determined to be mycoplasma-free. Lipofectamine 3000 (Invitrogen, Thermo Fisher Scientific, L3000-015) was used for transient transfection according to the manufacturer’s instructions.

To generate FUBP1- or PRMT5-depleted stable cells, cells were infected by viruses containing corresponding shRNAs produced by a 2-plasmid packaging system (psPAX2 and pMD2.g) and selected with 1 μg/mL puromycin for 4 days. To generate FUBP1-depleted/rescued stable cells, cells were infected by viruses produced by another 2-plasmid packaging system (VSVG and Gag) and selected by 200 μg/mL hygromycin B (Gibco, 10687010) for 7 days. Stable cell lines were verified by Western blot.

The following antibodies were used: FUBP1 (Abcam, ab192867), PRMT5 (Cell Signaling Technology, 79998), PRMT9 (Proteintech, 67365-1-Ig), PDK1 (Proteintech, 18262-1-AP), SLC7A11 (Proteintech, 26864-1-AP), FLAG (ABClonal, AE063), monomethyl arginine (Cell Signaling Technology, 8015), symmetric dimethyl arginine (Cell Signaling Technology, 13222), asymmetric dimethyl arginine (Cell Signaling Technology, 13522), BRD4 (Cell Signaling Technology, E2A7X), Myc (c#006-549), MTAP (Abcam, ab126770), SV40T (Abcam, 16879), α-SMA (Sigma-Aldrich, 2547), and actin (ABClonal, AC006).

Adenosine dialdehyde (AdOx) (Sigma-Aldrich, A7154), the PRMT5 inhibitor GSK591 (Selleck, S8111), I-BET151 (APExBIO, B1500), enzalutamide (MedChemExpress, HY70002), GATA2 inhibitor (MedChemExpress, HY-12743A), AICAR (Selleck, S1802), metformin (Selleck, S1950), glucose (Sigma-Aldrich, G7021), glutamine (Gibco, Thermo Fisher Scientific, 25030-081), putrescine (Sigma-Aldrich, P7505), spermidine (Sigma-Aldrich, S2501), spermine (Sigma-Aldrich, S1141), and FUBP1-IN-1 (MedChemExpress, HY-100758) were commercially obtained.

Point mutations of FUBP1 and PRMT5 were generated using Platinum Taq DNA Polymerase High Fidelity (Invitrogen, Thermo Fisher Scientific, 11304-011). Sequences for shRNAs and sgRNA are listed in Supplemental Table 2. All constructs were confirmed by DNA sequencing.

### meFUBP1 antibody.

meFUBP1 antibody was generated by Shanghai Ruixing Biotechnology Co. Ltd. To generate a site-specific antibody (meFUBP1) to detect the arginine-methylated FUBP1, synthesized peptide GPGPGGR(symMe2)GR(symMe2)GR(symMe2)GQGN (GL Biochem) was coupled to KLH as an antigen to immunize mice. Antiserum was collected after 5 doses of immunization.

### Immunoprecipitation–Western blotting.

Cells were lysed in 0.3%–0.5% NP-40 lysis buffer (50 mM Tris-HCl, pH 8.0, 150 mM NaCl, 0.3%–0.5% NP-40, 1 mM DTT) with protease inhibitor cocktail (MedChemExpress, HY-K0011) for 60 minutes. After centrifugation at 13,500*g* for 15 minutes, supernatants were incubated with FLAG beads for 4 hours at 4°C or with the primary antibody for 8 hours at 4°C, followed by a further 4 hours at 4°C with protein A beads (Santa Cruz Biotechnology, sc-2003). Beads were washed 4 times with NP-40 lysis buffer. Proteins were resuspended in the SDS loading buffer and subjected to SDS-PAGE.

### Immunohistochemistry and human tumor tissue microarray.

A tissue microarray was generated as previously described, with 107 matched prostate cancer and adjacent tissues from prostate cancer patients receiving prostatectomy (7).

Patient specimens were collected at Nanjing Drum Tower Hospital with patient consent under a hospital review board–approved protocol and in accordance with the Declaration of Helsinki. Written informed consent was obtained from each patient or related guardian. The diagnosis of human prostate cancer or normal tissue was confirmed based on histological analysis by independent pathologists.

Sections underwent deparaffinization, endogenous peroxidase elimination, and antigen retrieval followed by goat serum closure for 30 minutes. This was followed by overnight incubation at 4°C with primary antibodies at the following dilutions: FUBP1, 1:1,000; PRMT5, 1:2,000; meFUBP1, 1:1,000; SLC7A11, 1:100; and PDK1, 1:100. Secondary antibody incubation and DAB and H&E staining were then performed. The staining scores for tissues were classified into 4 groups: score 1, low staining; score 2, faint staining; score 3, moderate staining; score 4, strong staining. Scores 1 and 2 are defined as low expression and scores 3 and 4 as high expression.

### Immunoprecipitation–mass spectrometry.

The gel pieces were washed and destained, then reduced and alkylated with 10 mM dl-dithiothreitol (Sigma-Aldrich) and 55 mM iodoacetamide (Sigma-Aldrich) in 100 mM ammonium bicarbonate. In-gel digestion was performed in the presence of 50 mM ammonium bicarbonate using sequencing-grade soluble trypsin (Promega). The resulting peptides were extracted and re-dissolved in 100 mM ammonium bicarbonate before being digested in 0.2 μg chymotrypsin (Promega) overnight. The reaction was stopped by 5% formic acid and then desalted on a monospin C18 column (SHIMADZU-GL). The eluates were dried by speed vacuum and stored at –20°C.

Samples were solubilized in 0.1% formic acid, and loaded onto an in-house 30-cm-long pulled-tip analytical column (ReproSil-Pur C18 AQ, 1.9 μm particle size, Dr. Maisch GmbH; 75 μm inner × 360 μm outer diameter) connected to an Easy-nLC 1200 UHPLC (Thermo Fisher Scientific) for mass spectrometry (MS) analysis (Q Exactive Orbitrap mass spectrometer, Thermo Fisher Scientific). The elution gradient and mobile-phase constitution used for peptide separation were as follows: 0–1 minutes, 3%–6% B; 1–96 minutes, 6%–30% B; 96–114 minutes, 30%–60% B; 114–115 minutes, 60%–100% B; 115–120 minutes, 100%–100% B (mobile phase A: 0.1% formic acid in water; mobile phase B: 0.1% formic acid in 80% acetonitrile) at a flow rate of 300 nL/min. Peptides eluted from the LC column were directly electrosprayed into the mass spectrometer with the application of a distal 1.8 kV spray voltage. Survey full-scan MS spectra (from *m*/*z* 300 to 1,800) were acquired in the Orbitrap analyzer with resolution *r* =70,000 at *m*/*z* 400. The top 20 MS/MS events were sequentially generated and selected from the full MS spectrum at a 30% normalized collision energy. The dynamic exclusion time was 10 seconds.

The acquired MS/MS data were analyzed against a *Homo sapiens* UniProtKB database using Peaks Studio (version 8.5). During the database search, carbamidomethylation on cysteine was set as fixed modification, and methylation, dimethylation, and trimethylation on lysine or arginine and oxidation on methionine were set as variable modifications. To accurately estimate peptide probabilities and false discovery rates, a decoy database containing the reversed sequences of all the proteins appended to the target database was used. The peptide false discovery rate cutoff was set as 1%.

### Protein purification.

Plasmids encoding FLAG-PRMT5^WT^ or enzyme activity–dead mutations (FLAG-PRMT5^G365A/R368A^ and FLAG-PRMT5^E444Q^) were transfected into HEK293T cells for 36–48 hours. Cells were harvested for immunoprecipitation with FLAG beads. FLAG peptide (2 mg/mL) was used for eluting FLAG-PRMT5 from the FLAG beads for 30 minutes at 4°C. After centrifugation at 1,500*g* for 10 minutes, the supernatant was collected for Coomassie staining and further assay.

Plasmids encoding His-FUBP1^WT^ or FUBP1 with R359K/R361K/R363K mutations (FUBP1^3K^) were transformed into BL21 *E. coli* cells and cultured at 37°C in LB medium. Isopropyl-b-d-1-thiogalactopyranoside was added to a final concentration of 0.2 mM when OD_600_ was between 0.5 and 0.7 and cultured for 18 hours at 16°C. Cells were collected for protein purification according to the manufacturer’s instructions (Beyotime, P2226). Cell pellets were resuspended in lysis buffer, then lysed with a high-pressure cell disrupter, and centrifuged at 55,000*g* for 1 hour at 4°C. The supernatant containing FUBP1 protein was loaded onto 1 mL His-tag Purification Resin (Beyotime, P2226) pre-equilibrated with washing buffer, then eluted with elution buffer mixture containing 50 mM imidazole. The flow-through liquid was then loaded onto a Sepharose column for further purification. Fractions containing recombinant FUBP1 protein were collected and concentrated in concentrators with a molecular mass cutoff of 10 kDa (Sigma-Aldrich, 901008).

### In vitro methylation assay.

FLAG-tagged PRMT5 was mixed with the recombinant substrate (His-FUBP1) in methylation reaction buffer (50 mM Tris-HCl, pH 8.0, 5 mM DTT, 4 mM EDTA, 20 mM KCl) and incubated for 1 hour at 37°C with 200 μM *S*-adenosyl-l-methionine (Sigma-Aldrich, A4377) in a 30 μL final volume. Reactions were terminated by addition of SDS loading buffer and then boiled for 10 minutes for Western blotting analysis.

### His pull-down.

Purified His-FUBP1 and/or FLAG-PRMT5 fusion proteins were mixed overnight at 4°C. The mixture was incubated with His beads (Beyotime, P2226) for a further 2 hours at 4°C before the beads were harvested by centrifugation and washed 4 times with 0.3% NP-40 buffer. Samples were boiled in SDS loading buffer for Western blotting analysis.

### Chromatin immunoprecipitation and quantitative PCR.

Chromatin immunoprecipitation (ChIP)–quantitative PCR (qPCR) assay was performed according to a previously described protocol (64). Briefly, 1 × 10^7^ cells were cross-linked with 1% paraformaldehyde solution (Sigma-Aldrich, F8775) and then lysed and sonicated using the Covaris (S220) at duty 5 ultrasonicator (peak incident power [PIP], W) 200, burst 200, time 40 minutes). The supernatants were collected for immunoprecipitation with antibodies against rabbit IgG (negative control) or BRD4 (1:100) at 4°C overnight, followed by incubation with protein A beads for another 4 hours at 4°C. The beads were washed, and the cross-linking was reversed. Then, samples were digested with 20 μg proteinase K for 1 hour at 50°C. DNA fragments were purified using a kit according to the manufacturer’s instructions (Qiagen, 28104) and detected by qPCR. All primers are listed in Supplemental Table 2.

Total RNA was extracted using TRIzol Reagent (Invitrogen, 15596026CN), and cDNA was synthesized following the manufacturer’s instructions (Promega, M530A). Real-time PCR was performed using 2× SYBR qPCR Master Mix (EZBioscience, A0001-R2). Relative gene expression was calculated by the comparative Ct method, with *Actin* as an endogenous control. Primers are listed in Supplemental Table 2.

### Animal models.

Mice were maintained in a specific pathogen–free facility, and all studies were performed in compliance with the *Guide for the Care and Use of Laboratory Animals* (National Academies Press, 2011). All studies were approved by the IACUC of the Center for Excellence in Molecular Cell Science, Chinese Academy of Sciences. A C57BL/6 mouse model with mutations (R354K/R356K/R358K) at the mouse *Fubp1* locus was created by CRISPR/Cas9–mediated genome engineering (GemPharmatech). The sgRNA oligonucleotide sequence (5′-GGTGGTCGAGGACGAGGTAG-3′) and oligonucleotide donor sequence (5′-TTTGttctctcctttcttaggctggcaatcctggtggaccgggacctg gtggtaagggaaagggtaagggtcaaggaaactggaatatGGGcccccgggtggactccaggagtttaatt-3′) were designed. Cas9 mRNA and sgRNA generated by an in vitro transcription system were coinjected into fertilized eggs with a donor vector. The target region of the mouse *Fubp1* locus was confirmed by DNA sequencing. TRAMP transgenic mice were purchased from The Jackson Laboratory. *Probasin*-*Cre^+/–^* *Pten^fl/fl^* mice were provided by Gao Dong (Center for Excellence in Molecular Cell Science) (48).

### Synthesis of branched poly(β-amino ester).

Branched poly(β-amino ester (BPAE) with backbone-embedded and terminal-conjugated phenylboronic acids was synthesized as previously described (50). Briefly, BPAE was first synthesized via a facile Michael addition polycondensation reaction from diacrylate-containing monomer (A2), trimethylolpropane triacrylate (B3), and 4-amino-1-butanol (C2) at a molar ratio of 2:1:3.2, which was further end-capped with spermine (S) at an A2/S molar ratio of 1:1.1 followed by reaction with 4-(bromomethyl)phenylboronic acid (P) at an A2/P molar ratio of 6:1 to obtain the final polymer (Supplemental Figure 9A).

### Preparation of nanocomplexes.

BPAE was dissolved in sodium acetate (25 mM, pH 5.0) at a concentration of 5 mg/mL, and peptide was dissolved in sodium acetate (25 mM, pH 5.0) at a concentration of 1 mg/mL. The 2 solutions were mixed at equal volume and incubated at room temperature for 10 minutes to allow formation of BPAE/peptide nanocomplexes (NCs). NCs were diluted 5-fold with PBS (150 mM) before addition of hyaluronic acid (HA) at an HA/BPAE/peptide weight ratio of 50:5:1. The mixture was incubated for 10 minutes at room temperature to form HA/BPAE/peptide NCs. The NC particle size was determined by dynamic light scattering on a Malvern Zetasizer (Nano ZS90). The ζ potential of the NCs was also determined using the Malvern Zetasizer.

### Cellular internalization of BPAE/FITC-peptide NCs.

LNCaP cells were seeded on coverslips in 24-well plates (4 × 10^4^ cells per well) and incubated for 24 hours at 37°C. The culture medium was replaced by fresh medium, and the cells were treated with BPAE/FITC-peptide NCs (5 μg FITC-peptide/mL) for 8 hours.

### Cellular internalization of HA/BPAE/FITC-peptide NCs.

LNCaP cells were seeded on 12-well plates (1 × 10^5^ cells per well) and incubated for 24 hours at 37°C. The culture medium was replaced by fresh medium, and cells were treated with various HA/BPAE/FITC-peptide NCs (10 μg FITC-peptide/mL) for 8 hours. The cells were washed 3 times with PBS and subjected to flow cytometry analysis (Becton Dickinson). The cellular uptake level of HA/BPAE/FITC-peptide NCs was represented by the mean fluorescence intensity per cell.

### Xenograft mouse studies.

C4-2 *FUBP1*-depletion or *FUBP1*-depletion/rescue stable cells were suspended in PBS with Matrigel (Corning, 354234) at 1:2 (vol/vol). A 100 μL mixture with 5 × 10^6^ cells was subcutaneously injected in 6- to 8-week-old male NOD/SCID mice (Shanghai Lingchang Biotech). Tumor length and width were measured using calipers every 2 days. For NC-delivered peptide treatment, 2 × 10^6^ C4-2 cells were also subcutaneously injected into 6- to 8-week-old male NOD/SCID mice. Mice were randomly assigned to 1 of 3 groups when the xenograft volume reached approximately 150 mm^3^. Then, the mice were intraperitoneally injected with NC-delivered PUBLISH^WT^ or PUBLISH^3K^ peptide (5 μg/mL) every 2 days for 3 weeks, and tumor length and width were measured using calipers every 3 days. The volume was calculated using the formula 0.5 × (length × width^2^).

### RNA-Seq.

Total RNA from cells was extracted using TRIzol Reagent, and cDNA libraries were constructed using the VAHTS mRNA-seq V3 Library Prep Kit (Illumina, NR611) following the manufacturer’s instructions. Briefly, 100 ng of total RNA was used for mRNA purification and fragmentation. Purified mRNA was subjected to first- and second-strand cDNA synthesis. cDNA was then ligated to sequencing adapters (VAHTS RNA adapters set3–set6 for Illumina, N809/N810/N811/N812) and amplified by 14 cycles of PCR. The final libraries were evaluated using a Qubit Fluorometer (Invitrogen) and QIAxcel Advanced System (QIAGEN). Next, genome sequencing was performed by the Personal Biotechnology Company (Shanghai, China) using the Illumina Novaseq platform. Quality control of raw sequence data was evaluated by FastQC (v0.11.8; https://www.bioinformatics.babraham.ac.uk/projects/fastqc), and quality trimming and adapter clipping were performed using Cutadapt (v1.15; https://pypi.org/project/cutadapt/). Paired-end reads were aligned to the GRCh38.91 human reference genome using hisat2 (v2.0.5; http://daehwankimlab.github.io/hisat2). Gene expression levels were quantified by HTSeq (v0.9.1; https://htseq.readthedocs.io/en/latest). Counts were normalized using DESeq2 (v1.20.0; https://bioconductor.org/packages/release/bioc/html/DESeq2.html). Differential expression analyses were performed using DESeq2 based on the gene read count data. Biological triplicates were used in each treatment.

### Statistics.

Statistical analyses were conducted using GraphPad Prism 9.0 software. Survival incidence was assessed via the log-rank test, while Cox proportional hazards regression was used for multivariate analysis of predictive factors. Pearson’s correlation coefficients were calculated for correlation analyses. For comparisons between 2 groups, a 2-tailed Student’s *t* test was used. For multiple comparisons, we applied a 1-way ANOVA with Dunnett’s post hoc test for data sets exhibiting homogeneity of variance when comparing selected groups against a control. For data sets with heterogeneous variances, we used Brown-Forsythe/Welch ANOVAs with Dunnett’s T3 test. When the objective was to compare all groups, a 1-way ANOVA with Tukey’s test was used for homogeneous variances, and Brown-Forsythe/Welch ANOVAs with Dunnett’s T3 test for heterogeneous variances. All data in the figures are presented as mean ± SD. Statistical significance was set at a *P* value less than 0.05. Single and double asterisks denote *P* < 0.05 and *P* < 0.01, respectively.

### Study approval.

All mouse experiments were approved by the IACUC of the Center for Excellence in Molecular Cell Science, Chinese Academy of Sciences.

### Data availability.

All sequencing data generated during this study were deposited in the National Omics Data Encyclopedia (NODE; https://www.biosino.org/node) under accession number OEP 004234. Data included in this article are provided in the Supporting Data Values file and are also available upon request from the authors.

## Author contributions

WY, ZL, and LY designed the study. WY, SH, XL, XZ, PW, CP, and JC performed the experiments. XQ, DW, and SH generated the tissue microarray. WY, KX, ZW, LC, XH, ZL, LY, JQ, and LS analyzed the data. WY, SH, ZL, and LY contributed funding and wrote the manuscript. All authors discussed the results and commented on the manuscript.

## Supplementary Material

Supplemental data

Unedited blot and gel images

Supporting data values

## Version 1 08/15/2024

In-Press Preview

## Version 2 09/17/2024

Electronic publication

## References

[1] Siegel RL (2023). Cancer statistics, 2023. CA Cancer J Clin.

[2] Xia C (2022). Cancer statistics in China and United States, 2022: profiles, trends, and determinants. Chin Med J (Engl).

[3] Hou Z (2021). Androgens in prostate cancer: a tale that never ends. Cancer Lett.

[4] Auchus RJ, Sharifi N (2020). Sex hormones and prostate cancer. Annu Rev Med.

[5] de Bono JS (2011). Abiraterone and increased survival in metastatic prostate cancer. N Engl J Med.

[6] Scher HI (2012). Increased survival with enzalutamide in prostate cancer after chemotherapy. N Engl J Med.

[7] Tan Q (2022). Celastrol recruits UBE3A to recognize and degrade the DNA binding domain of steroid receptors. Oncogene.

[8] Mei Z (2022). Management of prostate cancer by targeting 3βHSD1 after enzalutamide and abiraterone treatment. Cell Rep Med.

[9] Zhang X (2023). Active DHEA uptake in the prostate gland correlates with aggressive prostate cancer. J Clin Invest.

[10] Watson PA (2015). Emerging mechanisms of resistance to androgen receptor inhibitors in prostate cancer. Nat Rev Cancer.

[11] Hou Z (2022). Inhibiting 3βHSD1 to eliminate the oncogenic effects of progesterone in prostate cancer. Cell Rep Med.

[12] Martinez-Jimenez F (2023). Pan-cancer whole-genome comparison of primary and metastatic solid tumours. Nature.

[13] Zhuang Q (2023). Prospective role of 3βHSD1 in prostate cancer precision medicine. Prostate.

[14] Takahashi K, Yamanaka S (2016). A decade of transcription factor-mediated reprogramming to pluripotency. Nat Rev Mol Cell Biol.

[15] Bushweller JH (2019). Targeting transcription factors in cancer – from undruggable to reality. Nat Rev Cancer.

[16] Asangani IA (2014). Therapeutic targeting of BET bromodomain proteins in castration-resistant prostate cancer. Nature.

[17] Labbe DP, Brown M (2018). Transcriptional regulation in prostate cancer. Cold Spring Harb Perspect Med.

[18] Adams EJ (2019). FOXA1 mutations alter pioneering activity, differentiation and prostate cancer phenotypes. Nature.

[19] Xie K (2022). Transcription factors as novel therapeutic targets and drivers of prostate cancer progression. Front Oncol.

[20] Benjamin LR (2008). Hierarchical mechanisms build the DNA-binding specificity of FUSE binding protein. Proc Natl Acad Sci U S A.

[21] Braddock DT (2022). Structure and dynamics of KH domains from FBP bound to single-stranded DNA. Nature.

[22] Seiler M (2018). Somatic mutational landscape of splicing factor genes and their functional consequences across 33 cancer types. Cell Rep.

[23] Samarin J (2016). PI3K/AKT/mTOR-dependent stabilization of oncogenic far-upstream element binding proteins in hepatocellular carcinoma cells. Hepatology.

[24] Singer S (2009). Coordinated expression of stathmin family members by far upstream sequence element-binding protein-1 increases motility in non-small cell lung cancer. Cancer Res.

[25] Byun J (2022). Cross-ancestry genome-wide meta-analysis of 61,047 cases and 947,237 controls identifies new susceptibility loci contributing to lung cancer. Nat Genet.

[26] Elman JS (2019). Identification of FUBP1 as a long tail cancer driver and widespread regulator of tumor suppressor and oncogene alternative splicing. Cell Rep.

[27] He L (2000). Loss of FBP function arrests cellular proliferation and extinguishes c-myc expression. EMBO J.

[28] Liu J (2001). Defective interplay of activators and repressors with TFIH in xeroderma pigmentosum. Cell.

[29] Weber A (2008). The FUSE binding proteins FBP1 and FBP3 are potential c-myc regulators in renal, but not in prostate and bladder cancer. BMC Cancer.

[30] Bedford MT, Clarke SG (2009). Protein arginine methylation in mammals: who, what, and why. Mol Cell.

[31] Jarrold J, Davies CC (2019). PRMTs and arginine methylation: cancer’s best-kept secret?. Trends Mol Med.

[32] Xu J, Richard S (2021). Cellular pathways influenced by protein arginine methylation: implications for cancer. Mol Cell.

[33] Yang Y, Bedford MT (2013). Protein arginine methyltransferases and cancer. Nat Rev Cancer.

[34] Mavrakis KJ (2016). Disordered methionine metabolism in MTAP/CDKN2A-deleted cancers leads to dependence on PRMT5. Science.

[35] Kryukov GV (2016). MTAP deletion confers enhanced dependency on the PRMT5 arginine methyltransferase in cancer cells. Science.

[36] Tang Z (2017). GEPIA: a web server for cancer and normal gene expression profiling and interactive analyses. Nucleic Acids Res.

[37] Gerhauser C (2018). Molecular evolution of early-onset prostate cancer identifies molecular risk markers and clinical trajectories. Cancer Cell.

[38] Li J (2020). A genomic and epigenomic atlas of prostate cancer in Asian populations. Nature.

[39] Castel P (2016). PDK1-SGK1 signaling sustains AKT-independent mTORC1 activation and confers resistance to PI3Kα inhibition. Cancer Cell.

[40] Tan J (2013). PDK1 signaling toward PLK1-MYC activation confers oncogenic transformation, tumor-initiating cell activation, and resistance to mTOR-targeted therapy. Cancer Discov.

[41] Zhang W (2021). RBMS1 regulates lung cancer ferroptosis through translational control of SLC7A11. J Clin Invest.

[42] Lang X (2019). Radiotherapy and immunotherapy promote tumoral lipid oxidation and ferroptosis via synergistic repression of SLC7A11. Cancer Discov.

[43] Beketova E (2022). PRMT5: a putative oncogene and therapeutic target in prostate cancer. Cancer Gene Ther.

[44] Blanc RS, Richard S (2017). Arginine methylation: the coming of age. Mol Cell.

[45] Yang Y (2015). PRMT9 is a type II methyltransferase that methylates the splicing factor SAP145. Nat Commun.

[46] Greenberg NM (1995). Prostate cancer in a transgenic mouse. Proc Natl Acad Sci U S A.

[47] Gingrich JR (1996). Metastatic prostate cancer in a transgenic mouse. Cancer Res.

[48] Trotman LC (2023). Pten dose dictates cancer progression in the prostate. PLoS Biol.

[49] Wang S (2003). Prostate-specific deletion of the murine Pten tumor suppressor gene leads to metastatic prostate cancer. Cancer Cell.

[50] Liu X (2022). Tailoring hyperbranched poly(β-amino ester) as a robust and universal platform for cytosolic protein delivery. Adv Mater.

[51] Hauck S (2016). Pyrazolo[1,5]apyrimidines as a new class of FUSE binding protein 1 (FUBP1) inhibitors. Bioorg Med Chem.

[52] Dobrovolskaite A (2022). Discovery of anthranilic acid derivatives as difluoromethylornithine adjunct agents that inhibit far upstream element binding protein 1 (FUBP1) function. J Med Chem.

[53] Rabenhorst U (2015). Single-stranded DNA-binding transcriptional regulator FUBP1 is essential for fetal and adult hematopoietic stem cell self-renewal. Cell Rep.

[54] Kim KH (2023). PRMT5 mediates FoxO1 methylation and subcellular localization to regulate lipophagy in myogenic progenitors. Cell Rep.

[55] Beketova E (2020). Protein arginine methyltransferase 5 promotes pICln-dependent androgen receptor transcription in castration-resistant prostate cancer. Cancer Res.

[56] Marjon K (2016). MTAP deletions in cancer create vulnerability to targeting of the MAT2A/PRMT5/RIOK1 axis. Cell Rep.

[57] Liu F (2017). PKM2 methylation by CARM1 activates aerobic glycolysis to promote tumorigenesis. Nat Cell Biol.

[58] Fedoriw A (2019). Anti-tumor activity of the type I PRMT inhibitor, GSK3368715, synergizes with PRMT5 inhibition through MTAP loss. Cancer Cell.

[59] Wang H (2017). Selective in vivo metabolic cell-labeling-mediated cancer targeting. Nat Chem Biol.

[60] Asangani IA (2016). BET bromodomain inhibitors enhance efficacy and disrupt resistance to AR antagonists in the treatment of prostate cancer. Mol Cancer Res.

[61] Welti J (2018). Targeting bromodomain and extra-terminal (BET) family proteins in castration-resistant prostate cancer (CRPC). Clin Cancer Res.

[62] Raina K (2016). PROTAC-induced BET protein degradation as a therapy for castration-resistant prostate cancer. Proc Natl Acad Sci U S A.

[63] Wyce A (2013). Inhibition of BET bromodomain proteins as a therapeutic approach in prostate cancer. Oncotarget.

[64] Li Z (2016). Redirecting abiraterone metabolism to fine-tune prostate cancer anti-androgen therapy. Nature.

