# Supplementary material for: PRMT5-mediated FUBP1 methylation accelerates prostate cancer progression
Source: J Clin Invest. 2024 Aug 15;134(18):e175023. doi: 10.1172/JCI175023 (PMC11405040; doi:10.1172/JCI175023)
Supplement: Supplemental data [file jci-134-175023-s100.pdf]

Figure legends for Supplemental data

Supplemental Figure 1. FUBP1 function in prostate cancer. A, Correlation between FUBP1 expression and disease progression in multiple cancers in the TCGA database. DFS, disease progression-free survival. **B**, Correlation of FUBP1 levels with biochemical recurrence in Chinese Prostate Cancer Genome and Epigenome Atlas (CPGEA). Log-rank test. **C,** Multivariate analysis of predictive factors for DFS of prostate cancer in the CPGEA dataset. HR, hazard ratios; 95% CI, 95% confidence interval. Cox proportional hazards regression. **D** and **E**, Expression of FUBP1 in prostatic tumor tissues and adjacent normal tissues. 2-tailed, unpaired student’s t test. **F**, Enriched pathways based on altered gene expression in FUBP1 knocked-down LNCaP cells. **G**, Genes showing alternative splicing after FUBP1 knockdown in LNCaP cells. 2-tailed, unpaired student’s t test. **H,** Enriched pathways based on alternative spliced genes regulated by FUBP1 in LNCaP cells. **I** and **J**, Effect of SLC7A11 and PDK1 on cell growth in prostate cancer cells. Scr, scrambled control shRNA. One-way ANOVA with Dunnett’s multiple-comparison test. **K**, FUBP1 knockdown efficiency in RWPE1. **L**, Effect of FUBP1 on cell growth in various cell lines. * $P < 0.05$; ** $P < 0.01$.

Supplemental Figure 2. FUBP1 methylation in HEK 293T cells. A, Potential FUBP1 methylation sites detected by mass spectrometry. **B** and **C**, Methylation status of FUBP1 and related mutants in HEK 293T cells. AdOx, adenosine dialdehyde; sDMA, symmetric dimethylarginine antibodies; FUBP1^{3K}, FUBP1 mutant with arginine-to-lysine mutated at R359/361/363. **D**, Dot-blot experiments to detect the specificity of meFUBP1, a site-specific antibody for methylated FUBP1 at R359/R361/R363. A

synthesized peptide (FUBP1 353-367 amino acids) with R359/R361/R363 methylation was used for dot-blot assay. **E**, Peptide competition assay to validate the specificity of meFUBP1. Synthesized methylated or unmethylated FUBP1 peptides were used for the competition. **F** and **G**, Specificity of meFUBP1 to detect intracellular FUBP1 methylation.

Supplemental Figure 3. Enrichment of His-FUBP1 and Flag-PRMT5 for in vitro assay. **A**, Purification of His-FUBP1. **B**, Enrichment of PRMT5 and its mutants from HEK 293T cells.

Supplemental Figure 4. Effect of various demethylases on FUBP1 methylation in LNCaP cells. Mixed siRNAs targeting different demethylases were transiently expressed in LNCaP cells, and endogenous FUBP1 methylation was detected. Scr, scrambled control siRNA.

Supplemental Figure 5. Generation of genetic FUBP1^{3K} knock-in mice. A, Schema for FUBP1^{3K} knock-in. **B**, Validation of FUBP1^{3k} knock-in mice. **C**, Representative H&E staining in *Fubp1*^{WT} and *Fubp1*^{3K} mice with different genetic backgrounds at age 25 weeks. Scale bars, 100 µm (upper) and 50 µm (lower), respectively. **D**, Expression of Slc7a11 and Pdk1 in different tissues of TRAMP^{+} mice. Scale bars, 50 µm.

Supplemental Figure 6. BRD4 regulates PRMT5-mediated FUBP1 methylation in VCaP cells. A, FUBP1 methylation after I-BET151 treatment in VCaP cells. I-BET151, a BRD4 inhibitor. **B**, Effect of I-BET151 on gene expression in VCaP cells. I-BET151, 2 µM. **C**, Enrichment of BRD4 on different gene promoters after I-BET151 treatment. **D** and **E**, Effect of BRD4 on FUBP1 methylation and its function in prostate cancer cell lines. **F**,

BRD4 enrichment on different gene promoters in VCaP cells. ** P < 0.01. n.s., not significant. One-way ANOVA with Dunnett's (T3) multiple-comparison test.

Supplemental Figure 7. Tissue microarray IHC results for meFUBP1 specificity. A and **B,** Specificity of meFUBP1 for IHC. LNCaP cell lysates (A) and LAPC4-derived xenograft (B) were used. Scale bar, 200 µm (upper) and 50 µm (lower), respectively. **C**, representative results of staining scores for different antibodies. Scale bar, 200 µm (left) and 100 µm (right) for each group, respectively.

Supplemental Figure 8. Schema for the FUBP1 truncations. N, N terminal; KH, K-homology motif; C, C terminal; NLS, nuclear localization sequence.

Supplemental Figure 9. Characterization of nanocomplexes. A, Synthetic route for branched poly (β-amino ester) (BPAE). BPAE was synthesized from diacrylate-containing monomer (A2), trimethylolpropane triacrylate (B3), and 4-amino-1-butanol (C2) at a molar ratio of 2:1:3.2. **B**, Size of BPAE/peptide NCs (BPAE/peptide = 2/1, w/w). **C**, Effect of PUBLISH and FUBP1 inhibitor on cell growth. FUBP1-IN-1, an FUBP1 inhibitor. One-way ANOVA with Turkey multiple-comparison test. **D** and **E**, Size and zeta potential of HA/BPAE/peptide NCs at various HA/BPAE weight ratios (n = 3). **F**, Mean fluorescence intensity (MFI) of LNCaP cells as determined by flow cytometry following 8-hour incubation with HA/BPAE/FITC-peptide NCs (10 µg FITC-peptide/mL) at various HA/BPAE weight ratios (n = 3). Results are shown as mean ± SD. ** P < 0.01. n.s., not significant.

Supplemental Table 1. IP-MS results for FUBP1 interacting proteins.

Supplemental Table 2. Sequence information for primers, siRNAs, and shRNAs.

Figure S1

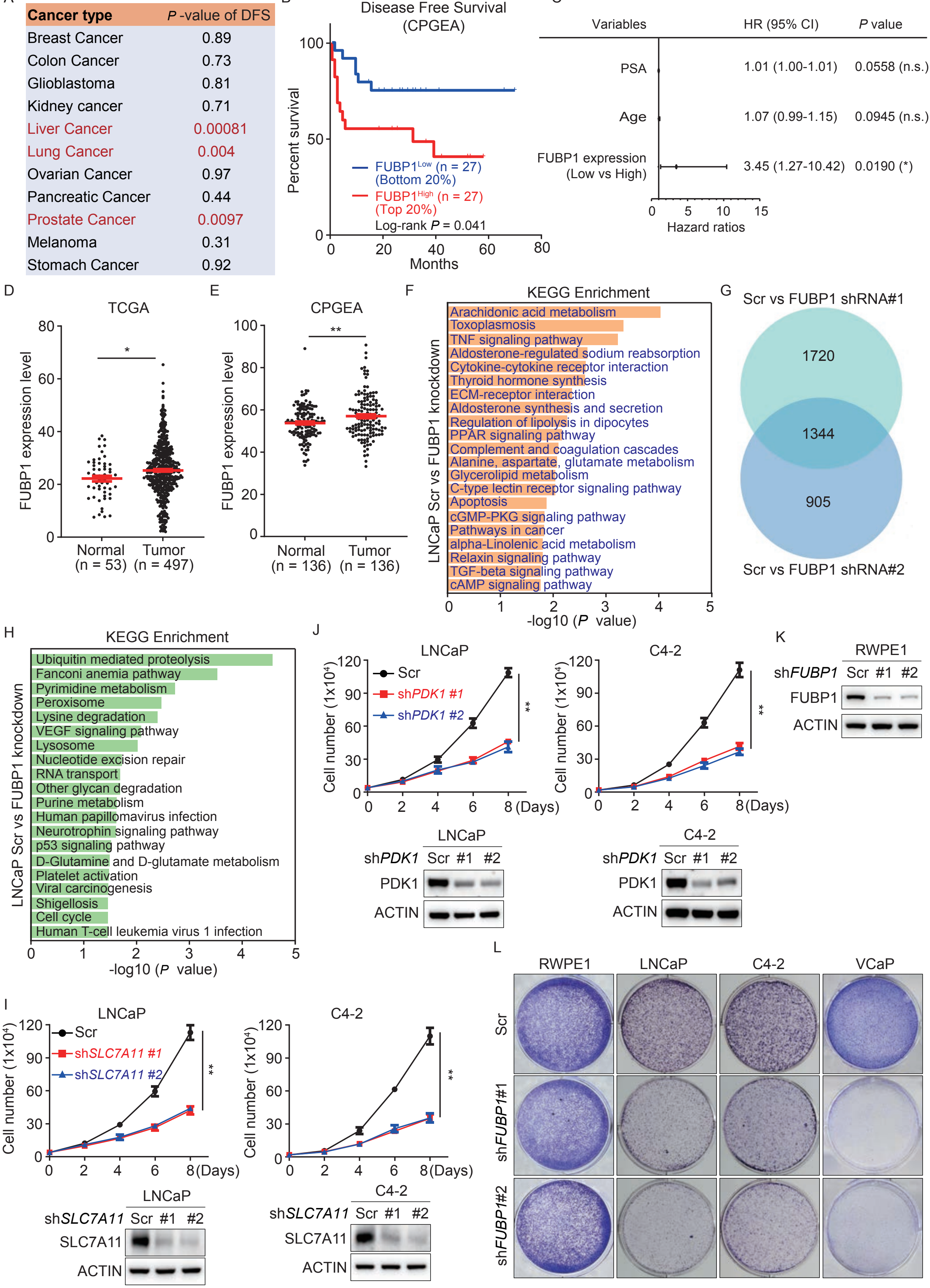
A
Cancer type | P -value of DFS
Breast Cancer 0.89
Colon Cancer 0.73
Glioblastoma 0.81
Kidney cancer 0.71
Liver Cancer 0.00081
Lung Cancer 0.004
Ovarian Cancer 0.97
Pancreatic Cancer 0.44
Prostate Cancer 0.0097
Melanoma 0.31
Stomach Cancer 0.92
B
Disease Free Survival (CPGEA)
Percent survival
Months
FUBP1Low (n = 27) (Bottom 20%)
FUBP1High (n = 27) (Top 20%)
Log-rank P = 0.041
C
Variables
HR (95% CI)
P value
PSA 1.01 (1.00-1.01) 0.0558 (n.s.)
Age 1.07 (0.99-1.15) 0.0945 (n.s.)
FUBP1 expression (Low vs High) 3.45 (1.27-10.42) 0.0190 (*)
Hazard ratios
D
TCGA
FUBP1 expression level
Normal (n = 53)
Tumor (n = 497)
E
CPGEA
FUBP1 expression level
Normal (n = 136)
Tumor (n = 136)
F
KEGG Enrichment
LNCaP Scr vs FUBP1 knockdown
Arachidonic acid metabolism
Toxoplasmosis
TNF signaling pathway
Aldosterone-regulated sodium reabsorption
Cytokine-cytokine receptor interaction
Thyroid hormone synthesis
ECM-receptor interaction
Aldosterone synthesis and secretion
Regulation of lipolysis in dipocytes
PPAR signaling pathway
Complement and coagulation cascades
Alanine, aspartate, glutamate metabolism
Glycerolipid metabolism
C-type lectin receptor signaling pathway
Apoptosis
cGMP-PKG signaling pathway
Pathways in cancer
alpha-Linolenic acid metabolism
Relaxin signaling pathway
TGF-beta signaling pathway
cAMP signaling pathway
-log10 (P value)
G
Scr vs FUBP1 shRNA#1
1720
1344
905
Scr vs FUBP1 shRNA#2
H
KEGG Enrichment
LNCaP Scr vs FUBP1 knockdown
Ubiquitin mediated proteolysis
Fanconi anemia pathway
Pyrimidine metabolism
Peroxisome
Lysine degradation
VEGF signaling pathway
Lysosome
Nucleotide excision repair
RNA transport
Other glycan degradation
Purine metabolism
Human papillomavirus infection
Neurotrophin signaling pathway
p53 signaling pathway
D-Glutamine and D-glutamate metabolism
Platelet activation
Viral carcinogenesis
Shigellosis
Cell cycle
Human T-cell leukemia virus 1 infection
-log10 (P value)
I
LNCaP
C4-2
Cell number (1x10⁴)
Scr
shSLC7A11 #1
shSLC7A11 #2
(Days)
shSLC7A11 Scr #1 #2
SLC7A11
ACTIN
J
LNCaP
C4-2
Cell number (1x10⁴)
Scr
shPDK1 #1
shPDK1 #2
(Days)
shPDK1 Scr #1 #2
PDK1
ACTIN
K
RWPE1
shFUBP1 Scr #1 #2
FUBP1
ACTIN
L
RWPE1
LNCaP
C4-2
VCaP
Scr
shFUBP1#1
shFUBP1#2

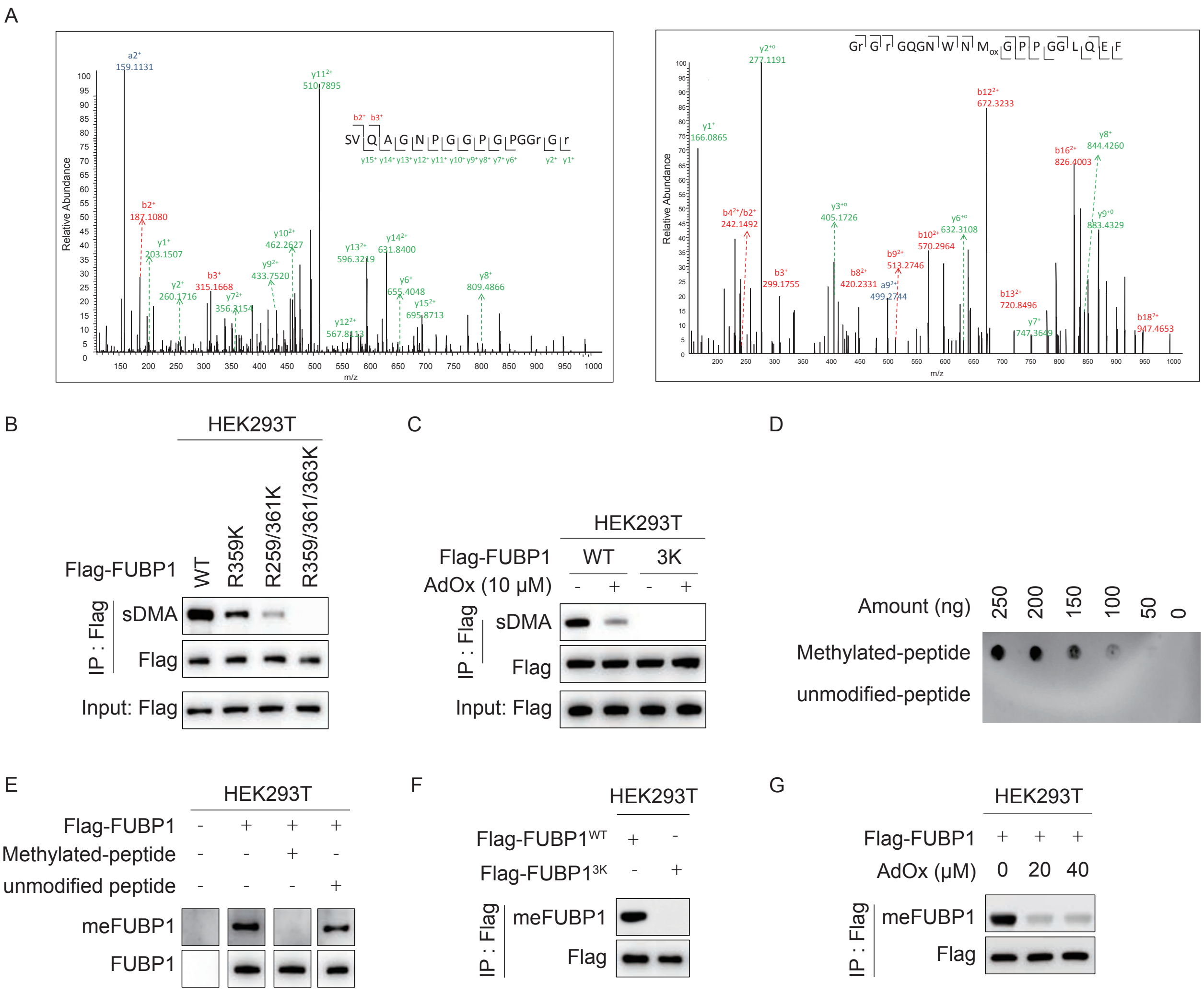
A
SV Q A G N P G G P G PGGr G r
GrGrGQGNWNMoxGPPGGLQEF
Relative Abundance
m/z
B
HEK293T
Flag-FUBP1
WT
R359K
R259/361K
R359/361/363K
IP : Flag
sDMA
Flag
Input: Flag
C
HEK293T
Flag-FUBP1
WT
3K
AdOx (10 μM)
IP : Flag
sDMA
Flag
Input: Flag
D
Amount (ng)
250
200
150
100
50
0
Methylated-peptide
unmodified-peptide
E
HEK293T
Flag-FUBP1
Methylated-peptide
unmodified peptide
meFUBP1
FUBP1
F
HEK293T
Flag-FUBP1WT
Flag-FUBP13K
IP : Flag
meFUBP1
Flag
G
HEK293T
Flag-FUBP1
AdOx (μM)
0
20
40
IP : Flag
meFUBP1
Flag

Figure S3

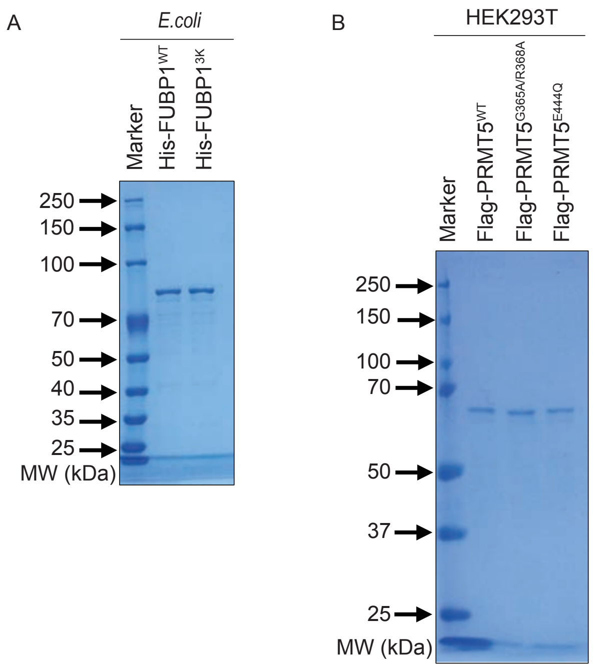
A
E.coli
Marker
His-FUBP1WT
His-FUBP13K
250
150
100
70
50
40
35
25
MW (kDa)
B
HEK293T
Marker
Flag-PRMT5WT
Flag-PRMT5G365A/R368A
Flag-PRMT5E444Q
250
150
100
70
50
37
25
MW (kDa)

Figure S4

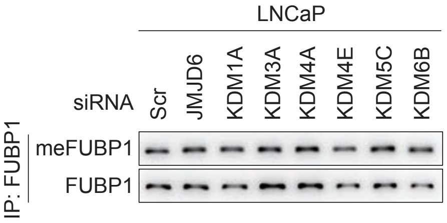

LNCaP
siRNA
Scr
JMJD6
KDM1A
KDM3A
KDM4A
KDM4E
KDM5C
KDM6B
IP: FUBP1
meFUBP1
FUBP1

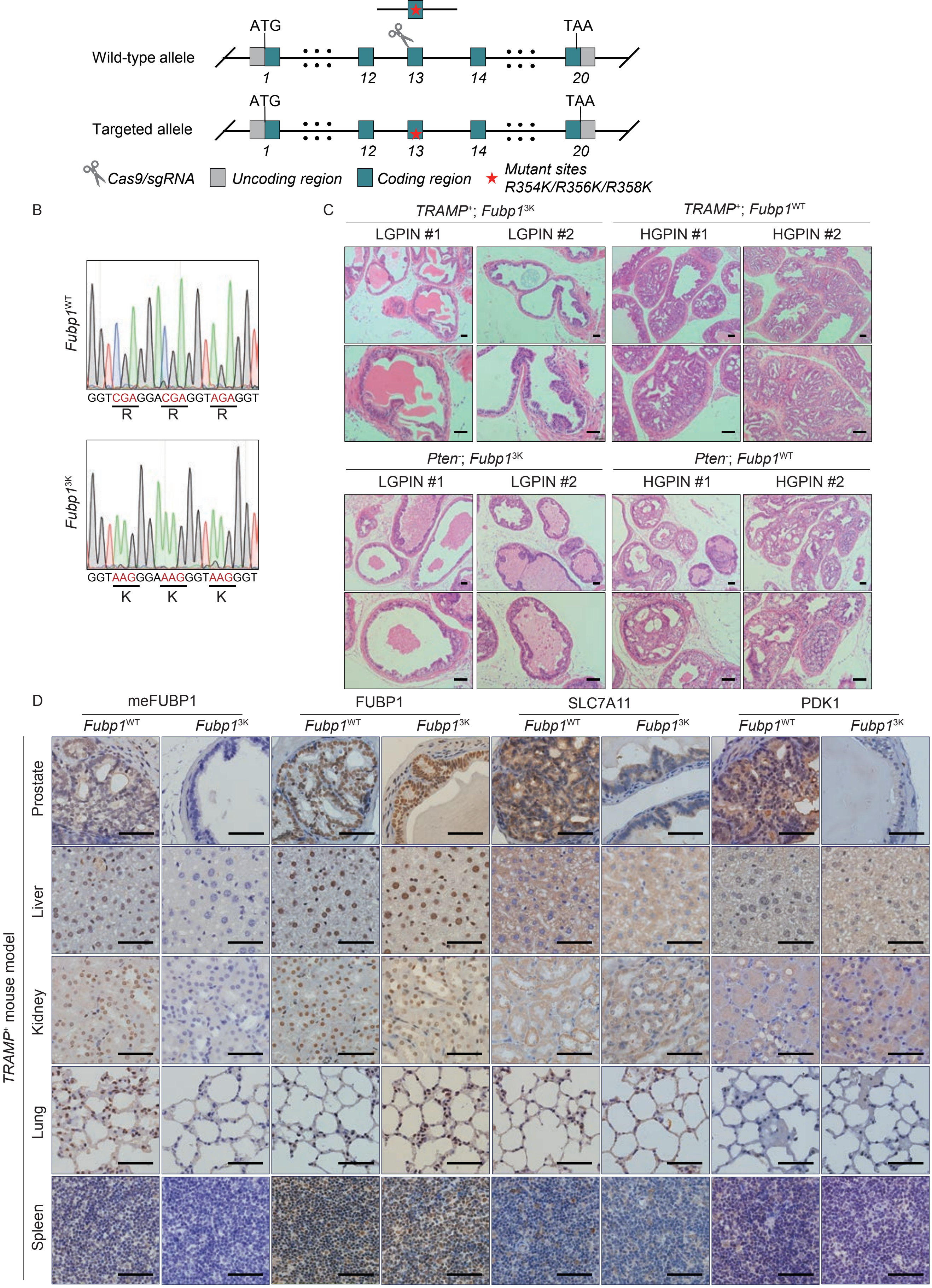

A
Donor and CRISPR/Cas9 System
ATG
TAA
Wild-type allele
1
12
13
14
20
Targeted allele
Cas9/sgRNA
Uncoding region
Coding region
Mutant sites
R354K/R356K/R358K
B
Fubp1WT
GGTCGAGGACGAGGTAGAGGT
R R R
Fubp13K
GGTAAGGGAAAGGGTAAGGGT
K K K
C
TRAMP+; Fubp13K
TRAMP+; FubP1WT
LGPIN #1
LGPIN #2
HGPIN #1
HGPIN #2
Pten-; Fubp13K
Pten-; Fubp1WT
D
meFUBP1
FUBP1
SLC7A11
PDK1
Fubp1WT
Fubp13K
Prostate
Liver
Kidney
Lung
Spleen
TRAMP+ mouse model

Figure S6

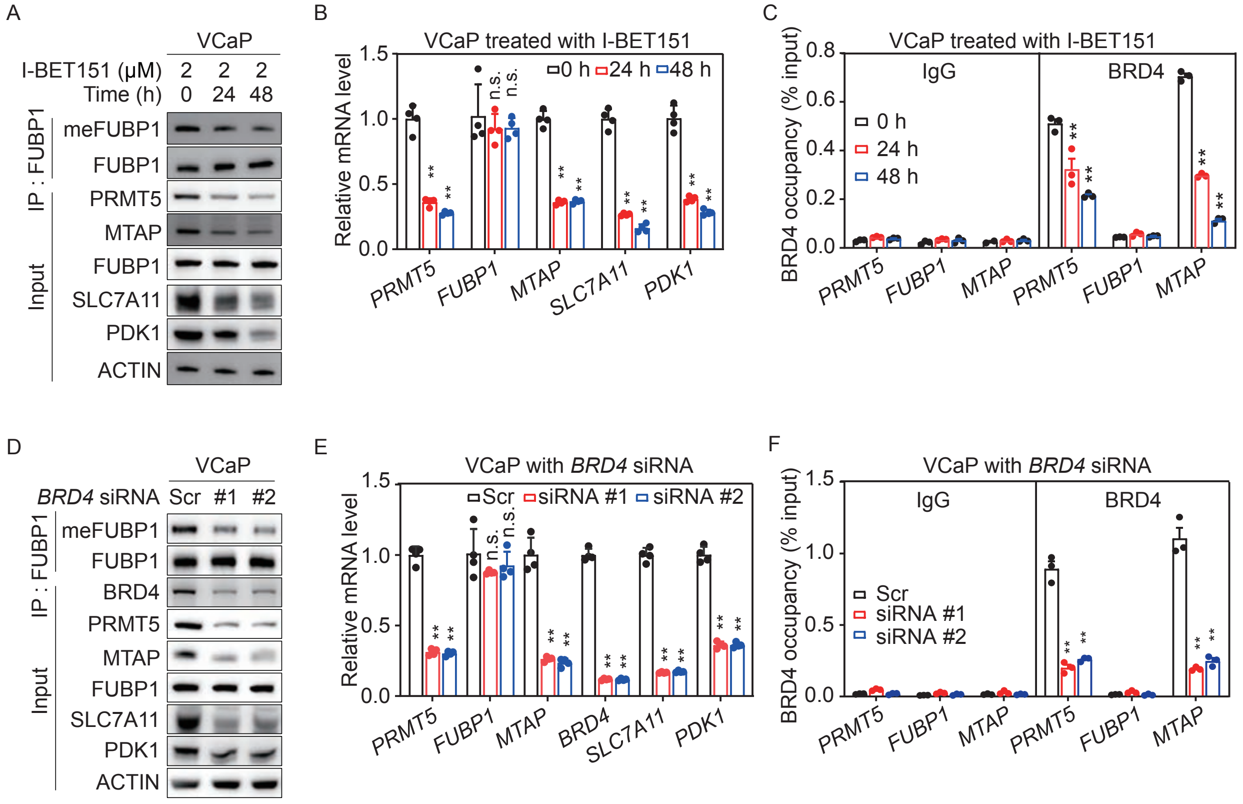
A
VCaP
I-BET151 (μM) 2 2 2
Time (h) 0 24 48
IP : FUBP1
meFUBP1
FUBP1
PRMT5
MTAP
Input
FUBP1
SLC7A11
PDK1
ACTIN
B
VCaP treated with I-BET151
Relative mRNA level
0 h 24 h 48 h
n.s.
PRMT5
FUBP1
MTAP
SLC7A11
PDK1
C
VCaP treated with I-BET151
BRD4 occupancy (% input)
IgG
BRD4
0 h
24 h
48 h
PRMT5
FUBP1
MTAP
D
VCaP
BRD4 siRNA Scr #1 #2
IP : FUBP1
meFUBP1
FUBP1
BRD4
PRMT5
MTAP
Input
FUBP1
SLC7A11
PDK1
ACTIN
E
VCaP with BRD4 siRNA
Relative mRNA level
Scr siRNA #1 siRNA #2
n.s.
PRMT5
FUBP1
MTAP
BRD4
SLC7A11
PDK1
F
VCaP with BRD4 siRNA
BRD4 occupancy (% input)
IgG
BRD4
Scr
siRNA #1
siRNA #2
PRMT5
FUBP1
MTAP

Figure S7

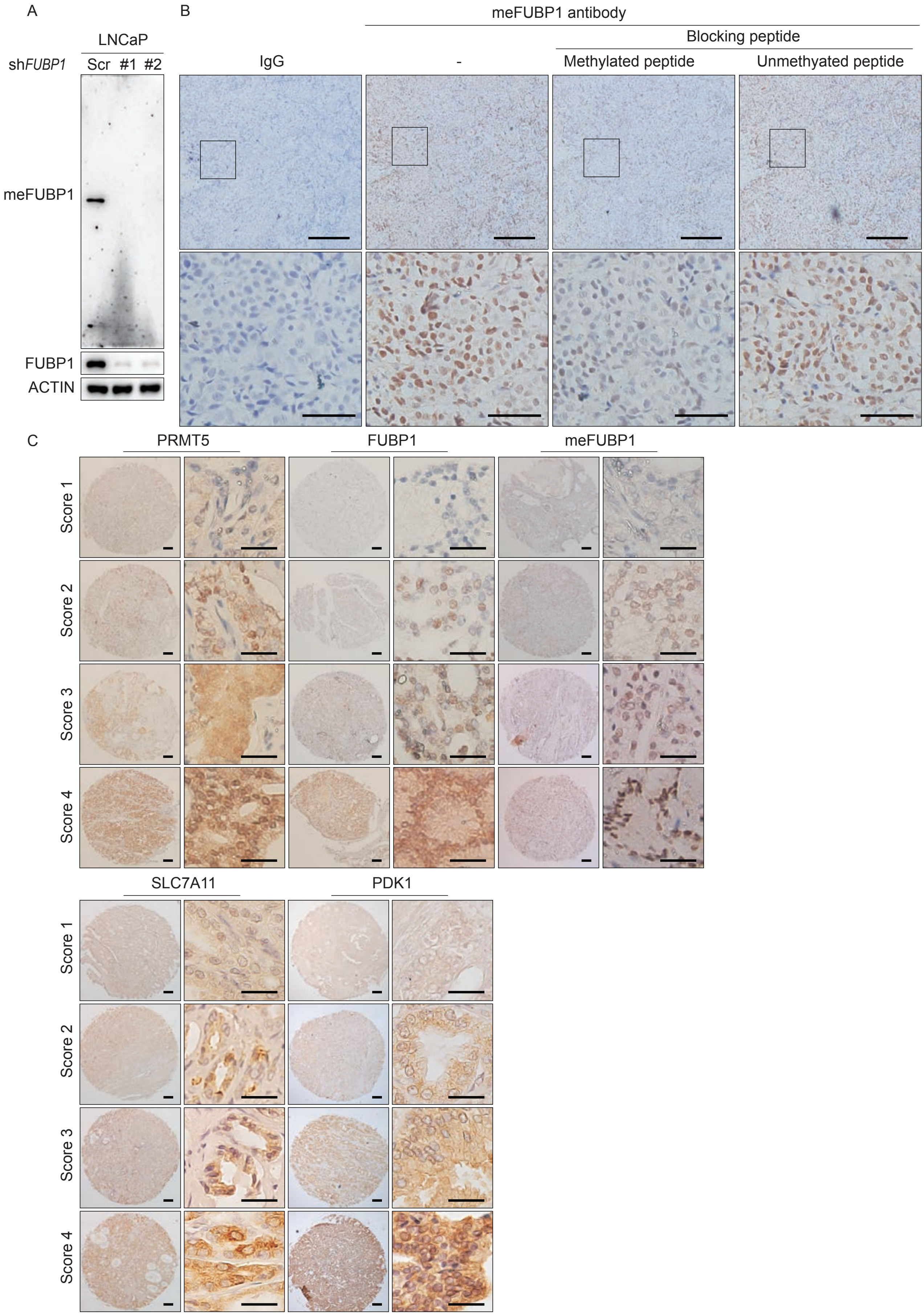

A
B
LNCaP
shFUBP1
Scr
#1
#2
meFUBP1
FUBP1
ACTIN
meFUBP1 antibody
Blocking peptide
IgG
-
Methylated peptide
Unmethyated peptide
C
PRMT5
FUBP1
meFUBP1
Score 1
Score 2
Score 3
Score 4
SLC7A11
PDK1
Score 1
Score 2
Score 3
Score 4

Figure S8

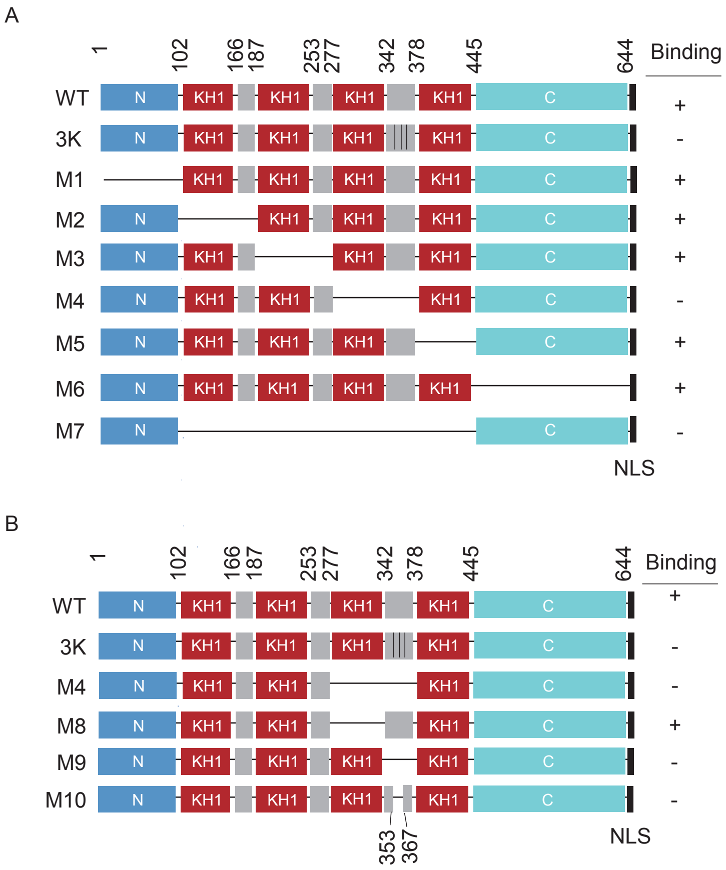
A
1
102
166
187
253
277
342
378
445
644
Binding
WT
3K
M1
M2
M3
M4
M5
M6
M7
N
KH1
C
+
-
NLS
B
1
102
166
187
253
277
342
378
445
644
Binding
WT
3K
M4
M8
M9
M10
N
KH1
C
+
-
353
367
NLS

Figure S9

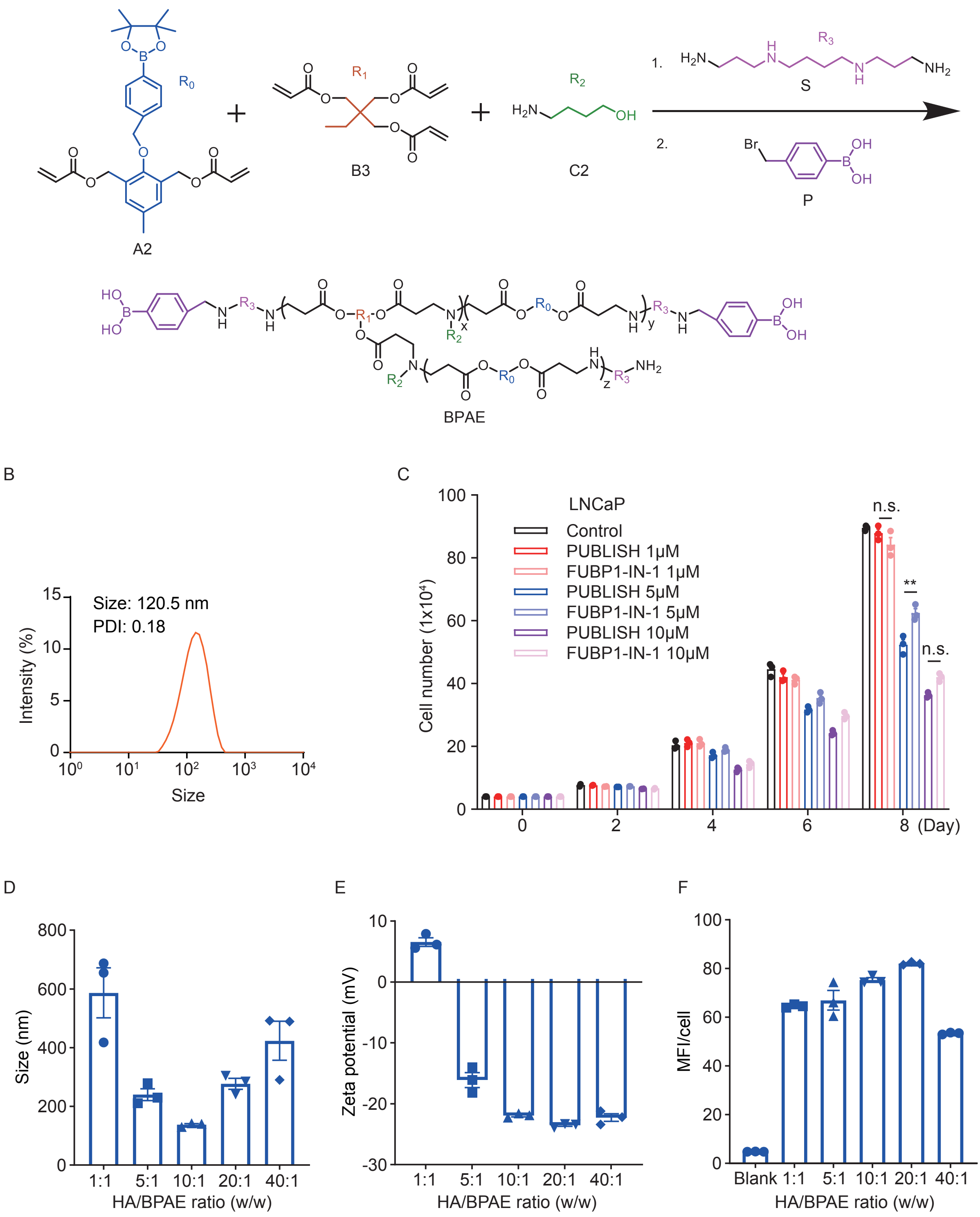

A
R0
R1
R2
R3
A2
B3
C2
S
P
1.
2.
BPAE
B
Size: 120.5 nm
PDI: 0.18
Intensity (%)
Size
C
LNCaP
Control
PUBLISH 1μM
FUBP1-IN-1 1μM
PUBLISH 5μM
FUBP1-IN-1 5μM
PUBLISH 10μM
FUBP1-IN-1 10μM
Cell number (1x10⁴)
(Day)
n.s.
**
n.s.
D
Size (nm)
HA/BPAE ratio (w/w)
E
Zeta potential (mV)
HA/BPAE ratio (w/w)
F
MFI/cell
Blank
HA/BPAE ratio (w/w)

Supplemental Table 1. IP-MS results for FUBP1 interacting proteins.

Accession	-10lgP	#Unique	Description
Q96AE4|FUBP1_HUMAN	426.84	246	Far upstream element-binding protein 1 OS=Homo sapiens OX=9606 GN=FUBP1 PE=1 SV=3
Q6PKG0|LARP1_HUMAN	329.24	68	La-related protein 1 OS=Homo sapiens OX=9606 GN=LARP1 PE=1 SV=2
Q92900|RENT1_HUMAN	327.66	56	Regulator of nonsense transcripts 1 OS=Homo sapiens OX=9606 GN=UPF1 PE=1 SV=2
P11940|PABP1_HUMAN	323.2	49	Polyadenylate-binding protein 1 OS=Homo sapiens OX=9606 GN=PABPC1 PE=1 SV=2
Q08211|DHX9_HUMAN	322.04	59	ATP-dependent RNA helicase A OS=Homo sapiens OX=9606 GN=DHX9 PE=1 SV=4
P52732|KIF11_HUMAN	320.21	67	Kinesin-like protein KIF11 OS=Homo sapiens OX=9606 GN=KIF11 PE=1 SV=2
Q13310|PABP4_HUMAN	314.84	32	Polyadenylate-binding protein 4 OS=Homo sapiens OX=9606 GN=PABPC4 PE=1 SV=1
P21333|FLNA_HUMAN	301.81	37	Filamin-A OS=Homo sapiens OX=9606 GN=FLNA PE=1 SV=4
Q9HCE1|MOV10_HUMAN	296.09	51	Helicase MOV-10 OS=Homo sapiens OX=9606 GN=MOV10 PE=1 SV=2
P09651|ROA1_HUMAN	296.05	14	Heterogeneous nuclear ribonucleoprotein A1 OS=Homo sapiens OX=9606 GN=HNRNPA1 PE=1 SV=5
P22626|ROA2_HUMAN	295.12	33	Heterogeneous nuclear ribonucleoproteins A2/B1 OS=Homo sapiens OX=9606 GN=HNRNPA2B1 PE=1 SV=2
Q12906|ILF3_HUMAN	290.74	39	Interleukin enhancer-binding factor 3 OS=Homo sapiens OX=9606 GN=ILF3 PE=1 SV=3
Q00839|HNRPU_HUMAN	290.59	58	Heterogeneous nuclear ribonucleoprotein U OS=Homo sapiens OX=9606 GN=HNRNPU PE=1 SV=6
O60506|HNRPQ_HUMAN	284.29	24	Heterogeneous nuclear ribonucleoprotein Q OS=Homo sapiens OX=9606 GN=SYNCRIP PE=1 SV=2
O14744|ANM5_HUMAN	283.29	39	Protein arginine N-methyltransferase 5 OS=Homo sapiens OX=9606 GN=PRMT5 PE=1 SV=4
P0DMV8|HS71A_HUMAN	279.35	15	Heat shock 70 kDa protein 1A OS=Homo sapiens OX=9606 GN=HSPA1A PE=1 SV=1
P0DMV9|HS71B_HUMAN	279.35	15	Heat shock 70 kDa protein 1B OS=Homo sapiens OX=9606 GN=HSPA1B PE=1 SV=1
P11142|HSP7C_HUMAN	275.43	29	Heat shock cognate 71 kDa protein OS=Homo sapiens OX=9606 GN=HSPA8 PE=1 SV=1
Q9H0D6|XRN2_HUMAN	275.36	42	5'-3' exoribonuclease 2 OS=Homo sapiens OX=9606 GN=XRN2 PE=1 SV=1
P51991|ROA3_HUMAN	275.12	26	Heterogeneous nuclear ribonucleoprotein A3 OS=Homo sapiens OX=9606 GN=HNRNPA3 PE=1 SV=2
Q9BUJ2|HNRL1_HUMAN	273.63	38	Heterogeneous nuclear ribonucleoprotein U-like protein 1 OS=Homo sapiens OX=9606 GN=HNRNPUL1 PE=1 SV=2
P43243|MATR3_HUMAN	272.85	33	Matrin-3 OS=Homo sapiens OX=9606 GN=MATR3 PE=1 SV=2
Q32P28|P3H1_HUMAN	272.19	34	Prolyl 3-hydroxylase 1 OS=Homo sapiens OX=9606 GN=P3H1 PE=1 SV=2
Q9NZI8|IF2B1_HUMAN	271.8	31	Insulin-like growth factor 2 mRNA-binding protein 1 OS=Homo sapiens OX=9606 GN=IGF2BP1 PE=1 SV=2
Q9H2U1|DHX36_HUMAN	270.93	29	ATP-dependent DNA/RNA helicase DHX36 OS=Homo sapiens OX=9606 GN=DHX36 PE=1 SV=2
Q9NZB2|F120A_HUMAN	270.42	29	Constitutive coactivator of PPAR-gamma-like protein 1 OS=Homo sapiens OX=9606 GN=FAM120A PE=1 SV=2
O43390|HNRPR_HUMAN	269.41	23	Heterogeneous nuclear ribonucleoprotein R OS=Homo sapiens OX=9606 GN=HNRNPR PE=1 SV=1
P14866|HNRPL_HUMAN	268.44	29	Heterogeneous nuclear ribonucleoprotein L OS=Homo sapiens OX=9606 GN=HNRNPL PE=1 SV=2
Q8N163|CCAR2_HUMAN	268.26	33	Cell cycle and apoptosis regulator protein 2 OS=Homo sapiens OX=9606 GN=CCAR2 PE=1 SV=2
O00425|IF2B3_HUMAN	267.82	21	Insulin-like growth factor 2 mRNA-binding protein 3 OS=Homo sapiens OX=9606 GN=IGF2BP3 PE=1 SV=2
P13645|K1C10_HUMAN	267.51	21	Keratin type I cytoskeletal 10 OS=Homo sapiens OX=9606 GN=KRT10 PE=1 SV=6
P19338|NUCL_HUMAN	264.97	25	Nucleolin OS=Homo sapiens OX=9606 GN=NCL PE=1 SV=3
P11021|BIP_HUMAN	263.25	23	Endoplasmic reticulum chaperone BiP OS=Homo sapiens OX=9606 GN=HSPA5 PE=1 SV=2

Q04637|IF4G1_HUMAN	262.53	34	Eukaryotic translation initiation factor 4 gamma 1 OS=Homo sapiens OX=9606 GN=EIF4G1 PE=1 SV=4
Q7L2E3|DHX30_HUMAN	260.21	41	ATP-dependent RNA helicase DHX30 OS=Homo sapiens OX=9606 GN=DHX30 PE=1 SV=1
Q32P51|RA1L2_HUMAN	260.06	1	Heterogeneous nuclear ribonucleoprotein A1-like 2 OS=Homo sapiens OX=9606 GN=HNRNPA1L2 PE=2 SV=2
P17844|DDX5_HUMAN	258.32	28	Probable ATP-dependent RNA helicase DDX5 OS=Homo sapiens OX=9606 GN=DDX5 PE=1 SV=1
Q09161|NCBP1_HUMAN	257.89	28	Nuclear cap-binding protein subunit 1 OS=Homo sapiens OX=9606 GN=NCBP1 PE=1 SV=1
Q71RC2|LARP4_HUMAN	257.34	28	La-related protein 4 OS=Homo sapiens OX=9606 GN=LARP4 PE=1 SV=3
P16989|YBOX3_HUMAN	257.16	20	Y-box-binding protein 3 OS=Homo sapiens OX=9606 GN=YBX3 PE=1 SV=4
P31943|HNRH1_HUMAN	257.06	10	Heterogeneous nuclear ribonucleoprotein H OS=Homo sapiens OX=9606 GN=HNRNPH1 PE=1 SV=4
Q02809|PLOD1_HUMAN	256.47	26	Procollagen-lysine 2-oxoglutarate 5-dioxygenase 1 OS=Homo sapiens OX=9606 GN=PLOD1 PE=1 SV=2
P52272|HNRPM_HUMAN	254.42	31	Heterogeneous nuclear ribonucleoprotein M OS=Homo sapiens OX=9606 GN=HNRNPM PE=1 SV=3
O43143|DHX15_HUMAN	252.95	29	Pre-mRNA-splicing factor ATP-dependent RNA helicase DHX15 OS=Homo sapiens OX=9606 GN=DHX15 PE=1 SV=2
P26599|PTBP1_HUMAN	252.07	19	Polypyrimidine tract-binding protein 1 OS=Homo sapiens OX=9606 GN=PTBP1 PE=1 SV=1
P13010|XRCC5_HUMAN	247.9	27	X-ray repair cross-complementing protein 5 OS=Homo sapiens OX=9606 GN=XRCC5 PE=1 SV=3
Q12905|ILF2_HUMAN	247.85	21	Interleukin enhancer-binding factor 2 OS=Homo sapiens OX=9606 GN=ILF2 PE=1 SV=2
P04264|K2C1_HUMAN	246.86	17	Keratin type II cytoskeletal 1 OS=Homo sapiens OX=9606 GN=KRT1 PE=1 SV=6
P61978|HNRPK_HUMAN	245.45	27	Heterogeneous nuclear ribonucleoprotein K OS=Homo sapiens OX=9606 GN=HNRNPK PE=1 SV=1
P10155|RO60_HUMAN	242.49	20	60 kDa SS-A/Ro ribonucleoprotein OS=Homo sapiens OX=9606 GN=RO60 PE=1 SV=2
Q9NUD5|ZCHC3_HUMAN	241.98	24	Zinc finger CCHC domain-containing protein 3 OS=Homo sapiens OX=9606 GN=ZCCHC3 PE=1 SV=2
Q15208|STK38_HUMAN	241.57	21	Serine/threonine-protein kinase 38 OS=Homo sapiens OX=9606 GN=STK38 PE=1 SV=1
Q9BQA1|MEP50_HUMAN	240.8	14	Methylosome protein 50 OS=Homo sapiens OX=9606 GN=WDR77 PE=1 SV=1
Q1KMD3|HNRL2_HUMAN	240.66	23	Heterogeneous nuclear ribonucleoprotein U-like protein 2 OS=Homo sapiens OX=9606 GN=HNRNPUL2 PE=1 SV=1
O15226|NKRF_HUMAN	240.42	27	NF-kappa-B-repressing factor OS=Homo sapiens OX=9606 GN=NKRF PE=1 SV=2
Q9UHX1|PUF60_HUMAN	240.05	21	Poly(U)-binding-splicing factor PUF60 OS=Homo sapiens OX=9606 GN=PUF60 PE=1 SV=1
Q9NYF8|BCLF1_HUMAN	239.48	26	Bcl-2-associated transcription factor 1 OS=Homo sapiens OX=9606 GN=BCLAF1 PE=1 SV=2
P62701|RS4X_HUMAN	239.21	14	40S ribosomal protein S4 X isoform OS=Homo sapiens OX=9606 GN=RPS4X PE=1 SV=2
O75127|PTCD1_HUMAN	237.35	23	Pentatricopeptide repeat-containing protein 1 mitochondrial OS=Homo sapiens OX=9606 GN=PTCD1 PE=1 SV=2
P12956|XRCC6_HUMAN	237.29	23	X-ray repair cross-complementing protein 6 OS=Homo sapiens OX=9606 GN=XRCC6 PE=1 SV=2
Q96KR1|ZFR_HUMAN	236.85	17	Zinc finger RNA-binding protein OS=Homo sapiens OX=9606 GN=ZFR PE=1 SV=2
Q15717|ELAV1_HUMAN	236.06	17	ELAV-like protein 1 OS=Homo sapiens OX=9606 GN=ELAVL1 PE=1 SV=2
P67809|YBOX1_HUMAN	235.03	14	Nuclease-sensitive element-binding protein 1 OS=Homo sapiens OX=9606 GN=YBX1 PE=1 SV=3
Q6P2Q9|PRP8_HUMAN	233.62	24	Pre-mRNA-processing-splicing factor 8 OS=Homo sapiens OX=9606 GN=PRPF8 PE=1 SV=2
P07237|PDIA1_HUMAN	232.2	23	Protein disulfide-isomerase OS=Homo sapiens OX=9606 GN=P4HB PE=1 SV=3
P63261|ACTG_HUMAN	231.77	6	Actin cytoplasmic 2 OS=Homo sapiens OX=9606 GN=ACTG1 PE=1 SV=1
O15460|P4HA2_HUMAN	231.27	22	Prolyl 4-hydroxylase subunit alpha-2 OS=Homo sapiens OX=9606 GN=P4HA2 PE=1 SV=1
Q9NR30|DDX21_HUMAN	231.14	21	Nucleolar RNA helicase 2 OS=Homo sapiens OX=9606 GN=DDX21 PE=1 SV=5

O75533|SF3B1_HUMAN	231.04	24	Splicing factor 3B subunit 1 OS=Homo sapiens OX=9606 GN=SF3B1 PE=1 SV=3
P61247|RS3A_HUMAN	230.85	24	40S ribosomal protein S3a OS=Homo sapiens OX=9606 GN=RPS3A PE=1 SV=2
Q96PK6|RBM14_HUMAN	230.68	24	RNA-binding protein 14 OS=Homo sapiens OX=9606 GN=RBM14 PE=1 SV=2
Q14103|HNRPD_HUMAN	230.09	17	Heterogeneous nuclear ribonucleoprotein D0 OS=Homo sapiens OX=9606 GN=HNRNPD PE=1 SV=1
Q9NUL7|DDX28_HUMAN	229.14	20	Probable ATP-dependent RNA helicase DDX28 OS=Homo sapiens OX=9606 GN=DDX28 PE=1 SV=2
Q92615|LAR4B_HUMAN	228.67	26	La-related protein 4B OS=Homo sapiens OX=9606 GN=LARP4B PE=1 SV=3
Q71U36|TBA1A_HUMAN	228.37	0	Tubulin alpha-1A chain OS=Homo sapiens OX=9606 GN=TUBA1A PE=1 SV=1
P38646|GRP75_HUMAN	227.41	21	Stress-70 protein mitochondrial OS=Homo sapiens OX=9606 GN=HSPA9 PE=1 SV=2
P68363|TBA1B_HUMAN	227.17	1	Tubulin alpha-1B chain OS=Homo sapiens OX=9606 GN=TUBA1B PE=1 SV=1
Q9NYY8|FAKD2_HUMAN	226.4	21	FAST kinase domain-containing protein 2 mitochondrial OS=Homo sapiens OX=9606 GN=FASTKD2 PE=1 SV=1
P07437|TBB5_HUMAN	226.38	3	Tubulin beta chain OS=Homo sapiens OX=9606 GN=TUBB PE=1 SV=2
O00571|DDX3X_HUMAN	225.98	3	ATP-dependent RNA helicase DDX3X OS=Homo sapiens OX=9606 GN=DDX3X PE=1 SV=3
P23396|RS3_HUMAN	225.47	21	40S ribosomal protein S3 OS=Homo sapiens OX=9606 GN=RPS3 PE=1 SV=2
O75718|CRTAP_HUMAN	225.42	20	Cartilage-associated protein OS=Homo sapiens OX=9606 GN=CRTAP PE=1 SV=1
P38159|RBMX_HUMAN	225.18	25	RNA-binding motif protein X chromosome OS=Homo sapiens OX=9606 GN=RBMX PE=1 SV=3
O14979|HNRDL_HUMAN	224.04	13	Heterogeneous nuclear ribonucleoprotein D-like OS=Homo sapiens OX=9606 GN=HNRNPDL PE=1 SV=3
O75643|U520_HUMAN	223.95	21	U5 small nuclear ribonucleoprotein 200 kDa helicase OS=Homo sapiens OX=9606 GN=SNRNP200 PE=1 SV=2
Q15393|SF3B3_HUMAN	223.08	17	Splicing factor 3B subunit 3 OS=Homo sapiens OX=9606 GN=SF3B3 PE=1 SV=4
P68371|TBB4B_HUMAN	222.74	2	Tubulin beta-4B chain OS=Homo sapiens OX=9606 GN=TUBB4B PE=1 SV=1
P34931|HS71L_HUMAN	221.03	0	Heat shock 70 kDa protein 1-like OS=Homo sapiens OX=9606 GN=HSPA1L PE=1 SV=2
Q9Y6M1|IF2B2_HUMAN	220.72	14	Insulin-like growth factor 2 mRNA-binding protein 2 OS=Homo sapiens OX=9606 GN=IGF2BP2 PE=1 SV=2
Q92841|DDX17_HUMAN	219.11	18	Probable ATP-dependent RNA helicase DDX17 OS=Homo sapiens OX=9606 GN=DDX17 PE=1 SV=2
Q10570|CPSF1_HUMAN	218.04	21	Cleavage and polyadenylation specificity factor subunit 1 OS=Homo sapiens OX=9606 GN=CPSF1 PE=1 SV=2
P26368|U2AF2_HUMAN	217.83	15	Splicing factor U2AF 65 kDa subunit OS=Homo sapiens OX=9606 GN=U2AF2 PE=1 SV=4
P35527|K1C9_HUMAN	216.9	14	Keratin type I cytoskeletal 9 OS=Homo sapiens OX=9606 GN=KRT9 PE=1 SV=3
P15880|RS2_HUMAN	215.33	20	40S ribosomal protein S2 OS=Homo sapiens OX=9606 GN=RPS2 PE=1 SV=2
O15523|DDX3Y_HUMAN	214.2	1	ATP-dependent RNA helicase DDX3Y OS=Homo sapiens OX=9606 GN=DDX3Y PE=1 SV=2
P52597|HNRPF_HUMAN	213.93	14	Heterogeneous nuclear ribonucleoprotein F OS=Homo sapiens OX=9606 GN=HNRNPF PE=1 SV=3
P55795|HNRH2_HUMAN	213.84	7	Heterogeneous nuclear ribonucleoprotein H2 OS=Homo sapiens OX=9606 GN=HNRNPH2 PE=1 SV=1
Q96I51|RCC1L_HUMAN	213.66	16	RCC1-like G exchanging factor-like protein OS=Homo sapiens OX=9606 GN=RCC1L PE=1 SV=2
Q9Y2Z4|SYYM_HUMAN	213.13	17	Tyrosine--tRNA ligase mitochondrial OS=Homo sapiens OX=9606 GN=YARS2 PE=1 SV=2
P31942|HNRH3_HUMAN	212.59	13	Heterogeneous nuclear ribonucleoprotein H3 OS=Homo sapiens OX=9606 GN=HNRNPH3 PE=1 SV=2
P23284|PPIB_HUMAN	212.52	17	Peptidyl-prolyl cis-trans isomerase B OS=Homo sapiens OX=9606 GN=PPIB PE=1 SV=2
Q9BQE3|TBA1C_HUMAN	212.5	1	Tubulin alpha-1C chain OS=Homo sapiens OX=9606 GN=TUBA1C PE=1 SV=1
P35908|K22E_HUMAN	211.48	8	Keratin type II cytoskeletal 2 epidermal OS=Homo sapiens OX=9606 GN=KRT2 PE=1 SV=2

P38919|IF4A3_HUMAN	209.34	14	Eukaryotic initiation factor 4A-III OS=Homo sapiens OX=9606 GN=EIF4A3 PE=1 SV=4
Q9NXV2|KCTD5_HUMAN	208.43	10	BTB/POZ domain-containing protein KCTD5 OS=Homo sapiens OX=9606 GN=KCTD5 PE=1 SV=1
P07910|HNRPC_HUMAN	207.84	19	Heterogeneous nuclear ribonucleoproteins C1/C2 OS=Homo sapiens OX=9606 GN=HNRNPC PE=1 SV=4
Q96SI9|STRBP_HUMAN	207.48	15	Spermatid perinuclear RNA-binding protein OS=Homo sapiens OX=9606 GN=STRBP PE=1 SV=1
Q7Z417|NUFP2_HUMAN	207.39	13	Nuclear fragile X mental retardation-interacting protein 2 OS=Homo sapiens OX=9606 GN=NUFIP2 PE=1 SV=1
Q8WWM7|ATX2L_HUMAN	207.1	18	Ataxin-2-like protein OS=Homo sapiens OX=9606 GN=ATXN2L PE=1 SV=2
Q9BXP5|SRRT_HUMAN	206.46	25	Serrate RNA effector molecule homolog OS=Homo sapiens OX=9606 GN=SRRT PE=1 SV=1
Q13151|ROA0_HUMAN	205.82	10	Heterogeneous nuclear ribonucleoprotein A0 OS=Homo sapiens OX=9606 GN=HNRNPA0 PE=1 SV=1
Q15029|U5S1_HUMAN	202.92	14	116 kDa U5 small nuclear ribonucleoprotein component OS=Homo sapiens OX=9606 GN=EFTUD2 PE=1 SV=1
P39023|RL3_HUMAN	202.77	16	60S ribosomal protein L3 OS=Homo sapiens OX=9606 GN=RPL3 PE=1 SV=2
Q14498|RBM39_HUMAN	201.68	14	RNA-binding protein 39 OS=Homo sapiens OX=9606 GN=RBM39 PE=1 SV=2
Q96I24|FUBP3_HUMAN	201.23	6	Far upstream element-binding protein 3 OS=Homo sapiens OX=9606 GN=FUBP3 PE=1 SV=2
Q9UN86|G3BP2_HUMAN	199.4	12	Ras GTPase-activating protein-binding protein 2 OS=Homo sapiens OX=9606 GN=G3BP2 PE=1 SV=2
Q9Y2W1|TR150_HUMAN	199.17	12	Thyroid hormone receptor-associated protein 3 OS=Homo sapiens OX=9606 GN=THRAP3 PE=1 SV=2
P62241|RS8_HUMAN	198.74	11	40S ribosomal protein S8 OS=Homo sapiens OX=9606 GN=RPS8 PE=1 SV=2
Q96EP5|DAZP1_HUMAN	198.68	10	DAZ-associated protein 1 OS=Homo sapiens OX=9606 GN=DAZAP1 PE=1 SV=1
Q14681|KCTD2_HUMAN	198.16	6	BTB/POZ domain-containing protein KCTD2 OS=Homo sapiens OX=9606 GN=KCTD2 PE=1 SV=3
P63244|RACK1_HUMAN	196.01	10	Receptor of activated protein C kinase 1 OS=Homo sapiens OX=9606 GN=RACK1 PE=1 SV=3
Q9NYK5|RM39_HUMAN	195.91	15	39S ribosomal protein L39 mitochondrial OS=Homo sapiens OX=9606 GN=MRPL39 PE=1 SV=3
Q14152|EIF3A_HUMAN	195.53	17	Eukaryotic translation initiation factor 3 subunit A OS=Homo sapiens OX=9606 GN=EIF3A PE=1 SV=1
P62081|RS7_HUMAN	195.38	12	40S ribosomal protein S7 OS=Homo sapiens OX=9606 GN=RPS7 PE=1 SV=1
Q9H9J2|RM44_HUMAN	195.24	9	39S ribosomal protein L44 mitochondrial OS=Homo sapiens OX=9606 GN=MRPL44 PE=1 SV=1
Q7Z2W4|ZCCHV_HUMAN	195.18	9	Zinc finger CCCH-type antiviral protein 1 OS=Homo sapiens OX=9606 GN=ZC3HAV1 PE=1 SV=3
P08708|RS17_HUMAN	194.84	11	40S ribosomal protein S17 OS=Homo sapiens OX=9606 GN=RPS17 PE=1 SV=2
Q96E29|MTEF3_HUMAN	193.1	14	Transcription termination factor 3 mitochondrial OS=Homo sapiens OX=9606 GN=MTERF3 PE=1 SV=2
Q9P015|RM15_HUMAN	192.01	13	39S ribosomal protein L15 mitochondrial OS=Homo sapiens OX=9606 GN=MRPL15 PE=1 SV=1
P68133|ACTS_HUMAN	191.7	1	Actin alpha skeletal muscle OS=Homo sapiens OX=9606 GN=ACTA1 PE=1 SV=1
P68032|ACTC_HUMAN	191.7	1	Actin alpha cardiac muscle 1 OS=Homo sapiens OX=9606 GN=ACTC1 PE=1 SV=1
Q99700|ATX2_HUMAN	190.96	10	Ataxin-2 OS=Homo sapiens OX=9606 GN=ATXN2 PE=1 SV=2
P08621|RU17_HUMAN	190.32	13	U1 small nuclear ribonucleoprotein 70 kDa OS=Homo sapiens OX=9606 GN=SNRNP70 PE=1 SV=2
P17066|HSP76_HUMAN	190.31	0	Heat shock 70 kDa protein 6 OS=Homo sapiens OX=9606 GN=HSPA6 PE=1 SV=2
Q9NUL3|STAU2_HUMAN	190.29	12	Double-stranded RNA-binding protein Staufen homolog 2 OS=Homo sapiens OX=9606 GN=STAU2 PE=1 SV=2
P52292|IMA1_HUMAN	189.85	9	Importin subunit alpha-1 OS=Homo sapiens OX=9606 GN=KPNA2 PE=1 SV=1
O95793|STAU1_HUMAN	189.83	12	Double-stranded RNA-binding protein Staufen homolog 1 OS=Homo sapiens OX=9606 GN=STAU1 PE=1 SV=2
P05455|LA_HUMAN	189	14	Lupus La protein OS=Homo sapiens OX=9606 GN=SSB PE=1 SV=2

P62263|RS14_HUMAN	188.9	10	40S ribosomal protein S14 OS=Homo sapiens OX=9606 GN=RPS14 PE=1 SV=3
P39019|RS19_HUMAN	188.52	12	40S ribosomal protein S19 OS=Homo sapiens OX=9606 GN=RPS19 PE=1 SV=2
P62753|RS6_HUMAN	188.5	11	40S ribosomal protein S6 OS=Homo sapiens OX=9606 GN=RPS6 PE=1 SV=1
P08865|RSSA_HUMAN	188.42	8	40S ribosomal protein SA OS=Homo sapiens OX=9606 GN=RPSA PE=1 SV=4
P62995|TRA2B_HUMAN	187.21	9	Transformer-2 protein homolog beta OS=Homo sapiens OX=9606 GN=TRA2B PE=1 SV=1
P36578|RL4_HUMAN	187.03	15	60S ribosomal protein L4 OS=Homo sapiens OX=9606 GN=RPL4 PE=1 SV=5
P82650|RT22_HUMAN	186.85	13	28S ribosomal protein S22 mitochondrial OS=Homo sapiens OX=9606 GN=MRPS22 PE=1 SV=1
P05388|RLA0_HUMAN	186.73	8	60S acidic ribosomal protein P0 OS=Homo sapiens OX=9606 GN=RPLP0 PE=1 SV=1
O95232|LC7L3_HUMAN	186.67	10	Luc7-like protein 3 OS=Homo sapiens OX=9606 GN=LUC7L3 PE=1 SV=2
Q13435|SF3B2_HUMAN	186.16	15	Splicing factor 3B subunit 2 OS=Homo sapiens OX=9606 GN=SF3B2 PE=1 SV=2
Q6UN15|FIP1_HUMAN	185.73	9	Pre-mRNA 3'-end-processing factor FIP1 OS=Homo sapiens OX=9606 GN=FIP1L1 PE=1 SV=1
P49406|RM19_HUMAN	185.12	14	39S ribosomal protein L19 mitochondrial OS=Homo sapiens OX=9606 GN=MRPL19 PE=1 SV=2
Q13509|TBB3_HUMAN	184.4	0	Tubulin beta-3 chain OS=Homo sapiens OX=9606 GN=TUBB3 PE=1 SV=2
Q12926|ELAV2_HUMAN	184.18	6	ELAV-like protein 2 OS=Homo sapiens OX=9606 GN=ELAVL2 PE=1 SV=2
Q9UKV3|ACINU_HUMAN	184.17	9	Apoptotic chromatin condensation inducer in the nucleus OS=Homo sapiens OX=9606 GN=ACIN1 PE=1 SV=2
P25398|RS12_HUMAN	183.9	11	40S ribosomal protein S12 OS=Homo sapiens OX=9606 GN=RPS12 PE=1 SV=3
Q99613|EIF3C_HUMAN	180.86	13	Eukaryotic translation initiation factor 3 subunit C OS=Homo sapiens OX=9606 GN=EIF3C PE=1 SV=1
Q92945|FUBP2_HUMAN	179.45	4	Far upstream element-binding protein 2 OS=Homo sapiens OX=9606 GN=KHSRP PE=1 SV=4
Q13283|G3BP1_HUMAN	179.35	9	Ras GTPase-activating protein-binding protein 1 OS=Homo sapiens OX=9606 GN=G3BP1 PE=1 SV=1
Q99729|ROAA_HUMAN	179.14	9	Heterogeneous nuclear ribonucleoprotein A/B OS=Homo sapiens OX=9606 GN=HNRNPAB PE=1 SV=2
Q9C0J8|WDR33_HUMAN	178.99	15	pre-mRNA 3' end processing protein WDR33 OS=Homo sapiens OX=9606 GN=WDR33 PE=1 SV=2
Q9Y262|EIF3L_HUMAN	178.75	10	Eukaryotic translation initiation factor 3 subunit L OS=Homo sapiens OX=9606 GN=EIF3L PE=1 SV=1
Q9P2I0|CPSF2_HUMAN	177.93	10	Cleavage and polyadenylation specificity factor subunit 2 OS=Homo sapiens OX=9606 GN=CPSF2 PE=1 SV=2
Q9BYD1|RM13_HUMAN	176.96	11	39S ribosomal protein L13 mitochondrial OS=Homo sapiens OX=9606 GN=MRPL13 PE=1 SV=1
P46782|RS5_HUMAN	176.78	12	40S ribosomal protein S5 OS=Homo sapiens OX=9606 GN=RPS5 PE=1 SV=4
P62280|RS11_HUMAN	176.2	12	40S ribosomal protein S11 OS=Homo sapiens OX=9606 GN=RPS11 PE=1 SV=3
P09001|RM03_HUMAN	175.82	13	39S ribosomal protein L3 mitochondrial OS=Homo sapiens OX=9606 GN=MRPL3 PE=1 SV=1
Q86U42|PABP2_HUMAN	175.69	11	Polyadenylate-binding protein 2 OS=Homo sapiens OX=9606 GN=PABPN1 PE=1 SV=3
Q13405|RM49_HUMAN	175.68	9	39S ribosomal protein L49 mitochondrial OS=Homo sapiens OX=9606 GN=MRPL49 PE=1 SV=1
Q15366|PCBP2_HUMAN	175.44	5	Poly(rC)-binding protein 2 OS=Homo sapiens OX=9606 GN=PCBP2 PE=1 SV=1
P22090|RS4Y1_HUMAN	174.86	0	40S ribosomal protein S4 Y isoform 1 OS=Homo sapiens OX=9606 GN=RPS4Y1 PE=1 SV=2
Q9BYD3|RM04_HUMAN	174.81	10	39S ribosomal protein L4 mitochondrial OS=Homo sapiens OX=9606 GN=MRPL4 PE=1 SV=1
Q00577|PURA_HUMAN	174.71	8	Transcriptional activator protein Pur-alpha OS=Homo sapiens OX=9606 GN=PURA PE=1 SV=2
Q9NPE2|NGRN_HUMAN	174.6	10	Neugrin OS=Homo sapiens OX=9606 GN=NGRN PE=1 SV=2
P84098|RL19_HUMAN	174.43	9	60S ribosomal protein L19 OS=Homo sapiens OX=9606 GN=RPL19 PE=1 SV=1

Q5BKZ1|ZN326_HUMAN	173.8	12	DBIRD complex subunit ZNF326 OS=Homo sapiens OX=9606 GN=ZNF326 PE=1 SV=2
P0DN76|U2AF5_HUMAN	173.32	9	Splicing factor U2AF 35 kDa subunit-like protein OS=Homo sapiens OX=9606 GN=U2AF1L5 PE=1 SV=1
Q01081|U2AF1_HUMAN	173.32	9	Splicing factor U2AF 35 kDa subunit OS=Homo sapiens OX=9606 GN=U2AF1 PE=1 SV=3
P29558|RBMS1_HUMAN	172.52	8	RNA-binding motif single-stranded-interacting protein 1 OS=Homo sapiens OX=9606 GN=RBMS1 PE=1 SV=3
P84103|SRSF3_HUMAN	172.38	9	Serine/arginine-rich splicing factor 3 OS=Homo sapiens OX=9606 GN=SRSF3 PE=1 SV=1
P27635|RL10_HUMAN	172.32	13	60S ribosomal protein L10 OS=Homo sapiens OX=9606 GN=RPL10 PE=1 SV=4
O15371|EIF3D_HUMAN	171.1	7	Eukaryotic translation initiation factor 3 subunit D OS=Homo sapiens OX=9606 GN=EIF3D PE=1 SV=1
Q9Y6Y0|NS1BP_HUMAN	170.99	11	Influenza virus NS1A-binding protein OS=Homo sapiens OX=9606 GN=IVNS1ABP PE=1 SV=3
Q16629|SRSF7_HUMAN	170.73	10	Serine/arginine-rich splicing factor 7 OS=Homo sapiens OX=9606 GN=SRSF7 PE=1 SV=1
O15234|CASC3_HUMAN	169.95	10	Protein CASC3 OS=Homo sapiens OX=9606 GN=CASC3 PE=1 SV=2
Q9NXV6|CARF_HUMAN	169.73	6	CDKN2A-interacting protein OS=Homo sapiens OX=9606 GN=CDKN2AIP PE=1 SV=3
P42704|LPPRC_HUMAN	169.66	10	Leucine-rich PPR motif-containing protein mitochondrial OS=Homo sapiens OX=9606 GN=LRPPRC PE=1 SV=3
P46783|RS10_HUMAN	169.58	9	40S ribosomal protein S10 OS=Homo sapiens OX=9606 GN=RPS10 PE=1 SV=1
Q14444|CAPR1_HUMAN	168.67	9	Caprin-1 OS=Homo sapiens OX=9606 GN=CAPRIN1 PE=1 SV=2
Q14151|SAFB2_HUMAN	168.6	3	Scaffold attachment factor B2 OS=Homo sapiens OX=9606 GN=SAFB2 PE=1 SV=1
P46781|RS9_HUMAN	168.12	16	40S ribosomal protein S9 OS=Homo sapiens OX=9606 GN=RPS9 PE=1 SV=3
Q07955|SRSF1_HUMAN	167.78	9	Serine/arginine-rich splicing factor 1 OS=Homo sapiens OX=9606 GN=SRSF1 PE=1 SV=2
P26196|DDX6_HUMAN	167.24	7	Probable ATP-dependent RNA helicase DDX6 OS=Homo sapiens OX=9606 GN=DDX6 PE=1 SV=2
Q9Y383|LC7L2_HUMAN	167.05	7	Putative RNA-binding protein Luc7-like 2 OS=Homo sapiens OX=9606 GN=LUC7L2 PE=1 SV=2
P30050|RL12_HUMAN	166.95	6	60S ribosomal protein L12 OS=Homo sapiens OX=9606 GN=RPL12 PE=1 SV=1
P02533|K1C14_HUMAN	166.74	7	Keratin type I cytoskeletal 14 OS=Homo sapiens OX=9606 GN=KRT14 PE=1 SV=4
O60832|DKC1_HUMAN	166.5	8	H/ACA ribonucleoprotein complex subunit DKC1 OS=Homo sapiens OX=9606 GN=DKC1 PE=1 SV=3
P05387|RLA2_HUMAN	166.43	6	60S acidic ribosomal protein P2 OS=Homo sapiens OX=9606 GN=RPLP2 PE=1 SV=1
Q7Z2W9|RM21_HUMAN	165.91	9	39S ribosomal protein L21 mitochondrial OS=Homo sapiens OX=9606 GN=MRPL21 PE=1 SV=2
Q15424|SAFB1_HUMAN	165.24	4	Scaffold attachment factor B1 OS=Homo sapiens OX=9606 GN=SAFB PE=1 SV=4
Q86V81|THOC4_HUMAN	164.15	9	THO complex subunit 4 OS=Homo sapiens OX=9606 GN=ALYREF PE=1 SV=3
Q8TD47|RS4Y2_HUMAN	164.06	0	40S ribosomal protein S4 Y isoform 2 OS=Homo sapiens OX=9606 GN=RPS4Y2 PE=2 SV=3
P62277|RS13_HUMAN	164.03	13	40S ribosomal protein S13 OS=Homo sapiens OX=9606 GN=RPS13 PE=1 SV=2
Q8WYQ5|DGCR8_HUMAN	164	13	Microprocessor complex subunit DGCR8 OS=Homo sapiens OX=9606 GN=DGCR8 PE=1 SV=1
Q99459|CDC5L_HUMAN	163.68	9	Cell division cycle 5-like protein OS=Homo sapiens OX=9606 GN=CDC5L PE=1 SV=2
P62249|RS16_HUMAN	163.52	9	40S ribosomal protein S16 OS=Homo sapiens OX=9606 GN=RPS16 PE=1 SV=2
P62829|RL23_HUMAN	163.4	7	60S ribosomal protein L23 OS=Homo sapiens OX=9606 GN=RPL23 PE=1 SV=1
Q8N5N7|RM50_HUMAN	162.46	7	39S ribosomal protein L50 mitochondrial OS=Homo sapiens OX=9606 GN=MRPL50 PE=1 SV=2
Q02878|RL6_HUMAN	162.4	8	60S ribosomal protein L6 OS=Homo sapiens OX=9606 GN=RPL6 PE=1 SV=3
Q9BRS2|RIOK1_HUMAN	162.21	8	Serine/threonine-protein kinase RIO1 OS=Homo sapiens OX=9606 GN=RIOK1 PE=1 SV=2

Q92552|RT27_HUMAN	161.55	8	28S ribosomal protein S27 mitochondrial OS=Homo sapiens OX=9606 GN=MRPS27 PE=1 SV=3
Q53F19|NCBP3_HUMAN	161.4	7	Nuclear cap-binding protein subunit 3 OS=Homo sapiens OX=9606 GN=NCBP3 PE=1 SV=2
P62750|RL23A_HUMAN	161.18	11	60S ribosomal protein L23a OS=Homo sapiens OX=9606 GN=RPL23A PE=1 SV=1
Q96CM3|RUSD4_HUMAN	160.63	12	Mitochondrial RNA pseudouridine synthase RPUSD4 OS=Homo sapiens OX=9606 GN=RPUSD4 PE=1 SV=1
P19474|RO52_HUMAN	160.34	13	E3 ubiquitin-protein ligase TRIM21 OS=Homo sapiens OX=9606 GN=TRIM21 PE=1 SV=1
Q13595|TRA2A_HUMAN	160.22	7	Transformer-2 protein homolog alpha OS=Homo sapiens OX=9606 GN=TRA2A PE=1 SV=1
P62266|RS23_HUMAN	160.11	9	40S ribosomal protein S23 OS=Homo sapiens OX=9606 GN=RPS23 PE=1 SV=3
P18621|RL17_HUMAN	159.84	11	60S ribosomal protein L17 OS=Homo sapiens OX=9606 GN=RPL17 PE=1 SV=3
Q13242|SRSF9_HUMAN	159.38	8	Serine/arginine-rich splicing factor 9 OS=Homo sapiens OX=9606 GN=SRSF9 PE=1 SV=1
P51398|RT29_HUMAN	158.8	10	28S ribosomal protein S29 mitochondrial OS=Homo sapiens OX=9606 GN=DAP3 PE=1 SV=1
Q5VTE0|EF1A3_HUMAN	158.57	10	Putative elongation factor 1-alpha-like 3 OS=Homo sapiens OX=9606 GN=EEF1A1P5 PE=5 SV=1
P68104|EF1A1_HUMAN	158.57	10	Elongation factor 1-alpha 1 OS=Homo sapiens OX=9606 GN=EEF1A1 PE=1 SV=1
Q9UMS4|PRP19_HUMAN	158.37	10	Pre-mRNA-processing factor 19 OS=Homo sapiens OX=9606 GN=PRPF19 PE=1 SV=1
P46777|RL5_HUMAN	158.03	8	60S ribosomal protein L5 OS=Homo sapiens OX=9606 GN=RPL5 PE=1 SV=3
P61254|RL26_HUMAN	157.94	3	60S ribosomal protein L26 OS=Homo sapiens OX=9606 GN=RPL26 PE=1 SV=1
Q9Y2H1|ST38L_HUMAN	157.85	7	Serine/threonine-protein kinase 38-like OS=Homo sapiens OX=9606 GN=STK38L PE=1 SV=3
P35268|RL22_HUMAN	157.46	6	60S ribosomal protein L22 OS=Homo sapiens OX=9606 GN=RPL22 PE=1 SV=2
Q9BYC9|RM20_HUMAN	156.82	7	39S ribosomal protein L20 mitochondrial OS=Homo sapiens OX=9606 GN=MRPL20 PE=1 SV=1
O95900|TRUB2_HUMAN	156.64	12	Mitochondrial mRNA pseudouridine synthase TRUB2 OS=Homo sapiens OX=9606 GN=TRUB2 PE=1 SV=1
Q9NRX2|RM17_HUMAN	156.58	10	39S ribosomal protein L17 mitochondrial OS=Homo sapiens OX=9606 GN=MRPL17 PE=1 SV=1
Q659C4|LAR1B_HUMAN	156.55	3	La-related protein 1B OS=Homo sapiens OX=9606 GN=LARP1B PE=1 SV=2
P62269|RS18_HUMAN	156.33	11	40S ribosomal protein S18 OS=Homo sapiens OX=9606 GN=RPS18 PE=1 SV=3
Q6P087|RUSD3_HUMAN	156.02	11	Mitochondrial mRNA pseudouridine synthase RPUSD3 OS=Homo sapiens OX=9606 GN=RPUSD3 PE=1 SV=3
Q05519|SRS11_HUMAN	155.38	7	Serine/arginine-rich splicing factor 11 OS=Homo sapiens OX=9606 GN=SRSF11 PE=1 SV=1
P55884|EIF3B_HUMAN	155.02	8	Eukaryotic translation initiation factor 3 subunit B OS=Homo sapiens OX=9606 GN=EIF3B PE=1 SV=3
Q9UKV8|AGO2_HUMAN	155	5	Protein argonaute-2 OS=Homo sapiens OX=9606 GN=AGO2 PE=1 SV=3
Q9UN81|LORF1_HUMAN	154.09	9	LINE-1 retrotransposable element ORF1 protein OS=Homo sapiens OX=9606 GN=L1RE1 PE=1 SV=1
Q9BQG0|MBB1A_HUMAN	153.14	7	Myb-binding protein 1A OS=Homo sapiens OX=9606 GN=MYBBP1A PE=1 SV=2
P54105|ICLN_HUMAN	152.64	6	Methylosome subunit pICln OS=Homo sapiens OX=9606 GN=CLNS1A PE=1 SV=1
P48634|PRC2A_HUMAN	152.25	8	Protein PRRC2A OS=Homo sapiens OX=9606 GN=PRRC2A PE=1 SV=3
Q9UKM9|RALY_HUMAN	151.61	9	RNA-binding protein Raly OS=Homo sapiens OX=9606 GN=RALY PE=1 SV=1
Q96DH6|MSI2H_HUMAN	150.88	3	RNA-binding protein Musashi homolog 2 OS=Homo sapiens OX=9606 GN=MSI2 PE=1 SV=1
P82933|RT09_HUMAN	150.78	6	28S ribosomal protein S9 mitochondrial OS=Homo sapiens OX=9606 GN=MRPS9 PE=1 SV=2
O75152|ZC11A_HUMAN	150.51	9	Zinc finger CCCH domain-containing protein 11A OS=Homo sapiens OX=9606 GN=ZC3H11A PE=1 SV=3
P26373|RL13_HUMAN	150.25	9	60S ribosomal protein L13 OS=Homo sapiens OX=9606 GN=RPL13 PE=1 SV=4

Q9Y676|RT18B_HUMAN	149.8	7	28S ribosomal protein S18b mitochondrial OS=Homo sapiens OX=9606 GN=MRPS18B PE=1 SV=1
P05141|ADT2_HUMAN	149.5	3	ADP/ATP translocase 2 OS=Homo sapiens OX=9606 GN=SLC25A5 PE=1 SV=7
O95639|CPSF4_HUMAN	149.4	5	Cleavage and polyadenylation specificity factor subunit 4 OS=Homo sapiens OX=9606 GN=CPSF4 PE=1 SV=1
P16403|H12_HUMAN	148.67	7	Histone H1.2 OS=Homo sapiens OX=9606 GN=HIST1H1C PE=1 SV=2
P10412|H14_HUMAN	148.67	7	Histone H1.4 OS=Homo sapiens OX=9606 GN=HIST1H1E PE=1 SV=2
P16402|H13_HUMAN	148.67	7	Histone H1.3 OS=Homo sapiens OX=9606 GN=HIST1H1D PE=1 SV=2

Supplemental Table 2. Sequence information for primers, siRNAs, and shRNAs.

Primer name	Species	Direction	Sequence (5'-3')	Application
shFUBP1 #1	Human	Forward	CCGGCGACTTGATGAAGATCTTAATCTCGAGATTAAGATCTTCATCAAGTCGTTTTTG	shRNA cloning
		Reverse	AATTCAAAAACGACTTGATGAAGATCTTAATCTCGAGATTAAGATCTTCATCAAGTCG	shRNA cloning
shFUBP1 #2	Human	Forward	CCGGGATTACAGGAGACCCATATAACTCGAGTTATATGGGTCTCCTGTAATC TTTTTG	shRNA cloning
		Reverse	AATTCAAAAAGATTACAGGAGACCCATATAACTCGAGTTATATGGGTCTCCTGTAATC	shRNA cloning
shSLC7A11 #1	Human	Forward	CCGGATAATAAAGAGATAATACGCTCGAGCGTATTATCTCTTTATTATTTTTTG	shRNA cloning
		Reverse	AATTCAAAAAATAATAAAGAGATAATACGCTCGAGCGTATTATCTCTTTATTAT	shRNA cloning
shSLC7A11 #2	Human	Forward	CCGGATATATGTGTAATGACCTCCTCGAGGAGGTCATTACACATATATTTTTTG	shRNA cloning
		Reverse	AATTCAAAAAATATATGTGTAATGACCTCCTCGAGGAGGTCATTACACATATAT	shRNA cloning
shPDK1 #1	Human	Forward	CCGGCGGATCAGAAACCGACACAATCTCGAGATTGTGTCGGTTTCTGATCCGTTTTTG	shRNA cloning
		Reverse	AATTCAAAAACGACTTGATGAAGATCTTAATCTCGAGATTGTGTCGGTTTCTGATCCG	shRNA cloning
shPDK1 #2	Human	Forward	CCGGGCTCTGTCAACAGACTCAATACTCGAGTATTGAGTCTGTTGACAGAGCTTTTTG	shRNA cloning
		Reverse	AATTCAAAAAGATTACAGGAGACCCATATAACTCGAGTATTGAGTCTGTTGACAGAGC	shRNA cloning
PRMT5-SgRNA #1	Human	Forward	GGTACCCTTGGTGGCACCAG	SgRNA cloning
		Reverse	CTGGTGCCACCAAGGGTACC	SgRNA cloning
PRMT5-SgRNA #2	Human	Forward	GGTGATGGCCAGTGTGGATG	SgRNA cloning
		Reverse	CATCCACACTGGCCATCACC	SgRNA cloning
PRMT5-siRNA #1	Human	Forward	GGACCUGAGAGAUGAUAUA	Gene knockdown
		Reverse	UAUAUCAUCUCUCAGGUCC	Gene knockdown
PRMT5-siRNA #2	Human	Forward	CCAGAAGAGGAGAAGGAUA	Gene knockdown
		Reverse	UAUCCUUCUCCUCUUCUGG	Gene knockdown
BRD4-siRNA #1	Human	Forward	GGACUAGAAACUUCCCAAA	Gene knockdown
		Reverse	UUUGGGAAGUUUCUAGUCC	Gene knockdown
BRD4-siRNA #2	Human	Forward	CACGGUACCAAACACAACU	Gene knockdown
		Reverse	AGUUGUGUUUGGUACCGUG	Gene knockdown
PRMT9-siRNA #1	Human	Forward	GGUAUCCAUUUGCCAACAA	Gene knockdown
		Reverse	UUGUUGGCAAAUGGAUACC	Gene knockdown
PRMT9-siRNA #2	Human	Forward	GCUUAACAACAUCCCAUAU	Gene knockdown
		Reverse	AUAUGGGAUGUUGUUAAGC	Gene knockdown
MTAP-siRNA #1	Human	Forward	GGAAGAGGGCUGUACACAU	Gene knockdown

		Reverse	AUGUGUACAGCCCUCUUCC	Gene knockdown
MTAP-siRNA #2	Human	Forward	GGAGUGUGCCAUAUUCCAA	Gene knockdown
		Reverse	UUGGAAUAUGGCACACUCC	Gene knockdown
JMJD6-siRNA #1	Human	Forward	GGGAGACCAAAGUUAUCAA	Gene knockdown
		Reverse	UUGAUAACUUUGGUCUCCC	Gene knockdown
JMJD6-siRNA #2	Human	Forward	CUGGCCACCUGAAUUCAAA	Gene knockdown
		Reverse	UUUGAAUUCAGGUGGCCAG	Gene knockdown
KDM1A-siRNA #1	Human	Forward	CCACGAGUCAAACCUUUAU	Gene knockdown
		Reverse	AUAAAGGUUUGACUCGUGG	Gene knockdown
KDM1A-siRNA #2	Human	Forward	GCCACCCAGAGAUAUUACU	Gene knockdown
		Reverse	AGUAAUAUCUCUGGGUGGC	Gene knockdown
KDM3A-siRNA #1	Human	Forward	CCUUGUGACAUGUGGUAAU	Gene knockdown
		Reverse	AUUACCACAUGUCACAAGG	Gene knockdown
KDM3A-siRNA #2	Human	Forward	GCGGGUAGAAGGCUUCUUA	Gene knockdown
		Reverse	UAAGAAGCCUUCUACCCGC	Gene knockdown
KDM4A-siRNA #1	Human	Forward	GCCUCUUUACUCAGUACAA	Gene knockdown
		Reverse	UUGUACUGAGUAAAGAGGC	Gene knockdown
KDM4A-siRNA #2	Human	Forward	GCCGUCAGCCUUUAAGCAA	Gene knockdown
		Reverse	UUGCUUAAAGGCUGACGGC	Gene knockdown
KDM4E-siRNA #1	Human	Forward	CCUGGAUCCGCAUUCCAAA	Gene knockdown
		Reverse	UUUGGAAUGCGGAUCCAGG	Gene knockdown
KDM4E-siRNA #2	Human	Forward	GGUCGUGGUCAUGGUUGUU	Gene knockdown
		Reverse	AACAACCAUGACCACGACC	Gene knockdown
KDM5C-siRNA #1	Human	Forward	GCAGAGAAAUCGGGCAUUU	Gene knockdown
		Reverse	AAAUGCCCGAUUUCUCUGC	Gene knockdown
KDM5C-siRNA #2	Human	Forward	CCUUUAAAGCUGACUACUU	Gene knockdown
		Reverse	AAGUAGUCAGCUUUAAAGG	Gene knockdown
KDM6B-siRNA #1	Human	Forward	GUGACAAGGAGACCUUUAU	Gene knockdown
		Reverse	AUAAAGGUCUCCUUGUCAC	Gene knockdown
KDM6B-siRNA #2	Human	Forward	GAGACCUCGUGUGGAUUAA	Gene knockdown
		Reverse	UUAAUCCACACGAGGUCUC	Gene knockdown
ACTIN	Human	Forward	GGTGATGGCCAGTGTGGATG	qPCR

		Reverse	GGTGATGGCCAGTGTGGATG	qPCR
FUBP1	Human	Forward	CCTGGAACTCCAATGGGACC	qPCR
		Reverse	GCGTAATAAGCAGCCCAAGC	qPCR
PRMT5	Human	Forward	TATGTGGTACGGCTGCACA	qPCR
		Reverse	TGGCTGAAGGTGAAACAGG	qPCR
MTAP	Human	Forward	ACCACCGCCGTGAAGATTG	qPCR
		Reverse	GCATCAGATGGCTTGCCAA	qPCR
BRD4	Human	Forward	GAGCTACCCACAGAAGAAACC	qPCR
		Reverse	GAGTCGATGCTTGAGTTGTGTT	qPCR
PDK1	Human	Forward	CTGTGATACGGATCAGAAACCG	qPCR
		Reverse	TCCACCAAACAATAAAGAGTGCT	qPCR
SLC7A11	Human	Forward	TCTCCAAAGGAGGTTACCTGC□	qPCR
		Reverse	AGACTCCCCTCAGTAAAGTGAC□	qPCR
FUBP1	Human	Forward	GCCTGAGGCCATTTTGAGAAA	ChIP-qPCR
		Reverse	CCATTCACCGTCACACCTCT	ChIP-qPCR
PRMT5	Human	Forward	AGCGCGAGGAGAAAGATG	ChIP-qPCR
		Reverse	CTATTTCGGGGACGCAATTC	ChIP-qPCR
MTAP	Human	Forward	GGGAGTTGTGCAAGGTCTCA	ChIP-qPCR
		Reverse	CCCAAGACTCAGGGATGACG	ChIP-qPCR
