# Supplementary material for: PRMT5-mediated FUBP1 methylation accelerates prostate cancer progression
Source: J Clin Invest. 2024 Aug 15;134(18):e175023. doi: 10.1172/JCI175023 (PMC11405040; doi:10.1172/JCI175023)
Supplement: Unedited blot and gel images [file jci-134-175023-s101.pdf]

Figure 1C

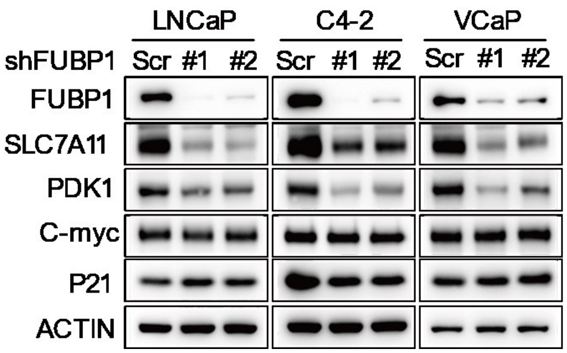
LNCaP
C4-2
VCaP
shFUBP1
Scr #1 #2
Scr #1 #2
Scr #1 #2
FUBP1
SLC7A11
PDK1
C-myc
P21
ACTIN

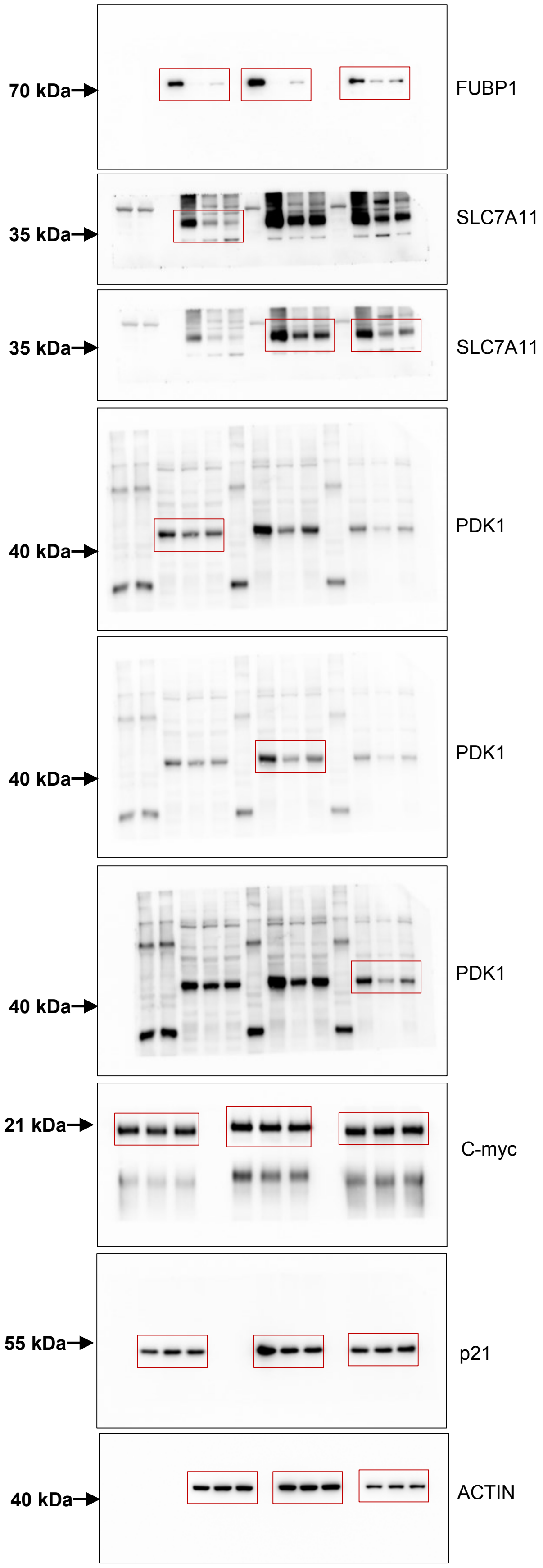
70 kDa
FUBP1
35 kDa
SLC7A11
35 kDa
SLC7A11
40 kDa
PDK1
40 kDa
PDK1
40 kDa
PDK1
21 kDa
C-myc
55 kDa
p21
40 kDa
ACTIN

Figure 1H

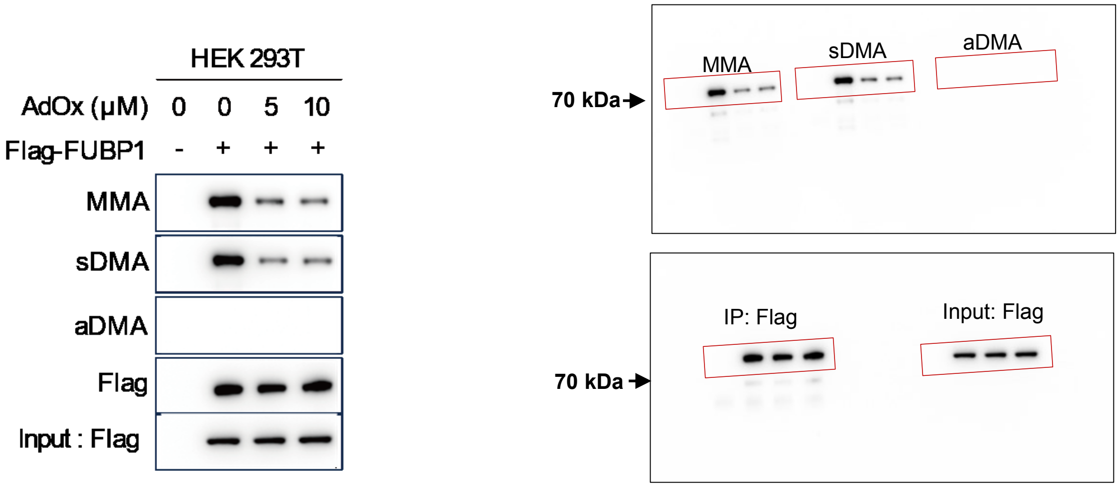

Figure 1J

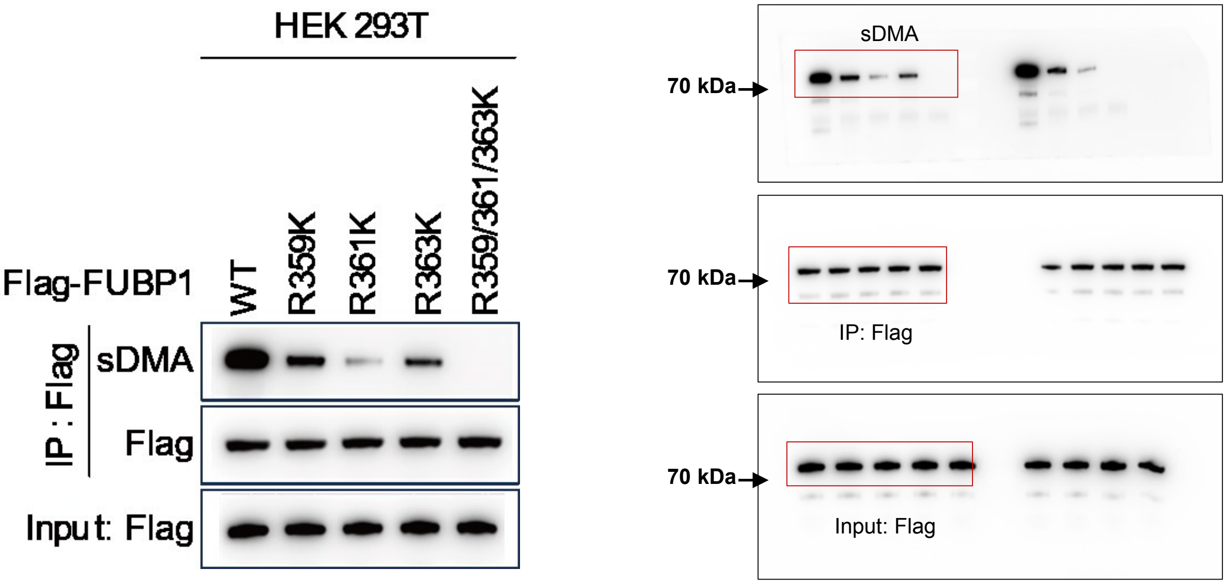

Figure 1K

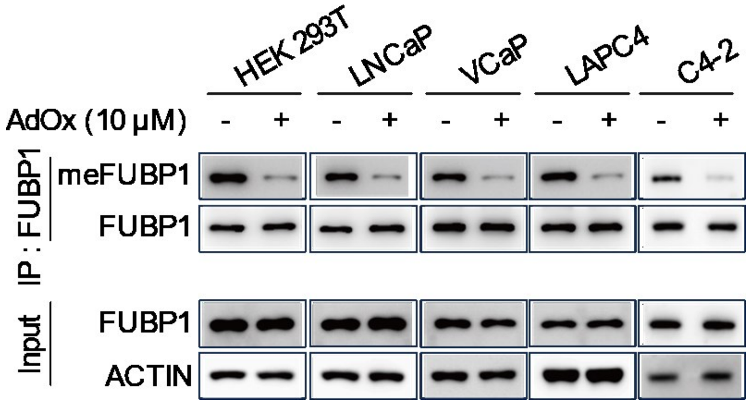
HEK 293T
LNCaP
VCaP
LAPC4
C4-2
AdOx (10 µM)
- + - + - + - + - +
IP : FUBP1
meFUBP1
FUBP1
Input
FUBP1
ACTIN

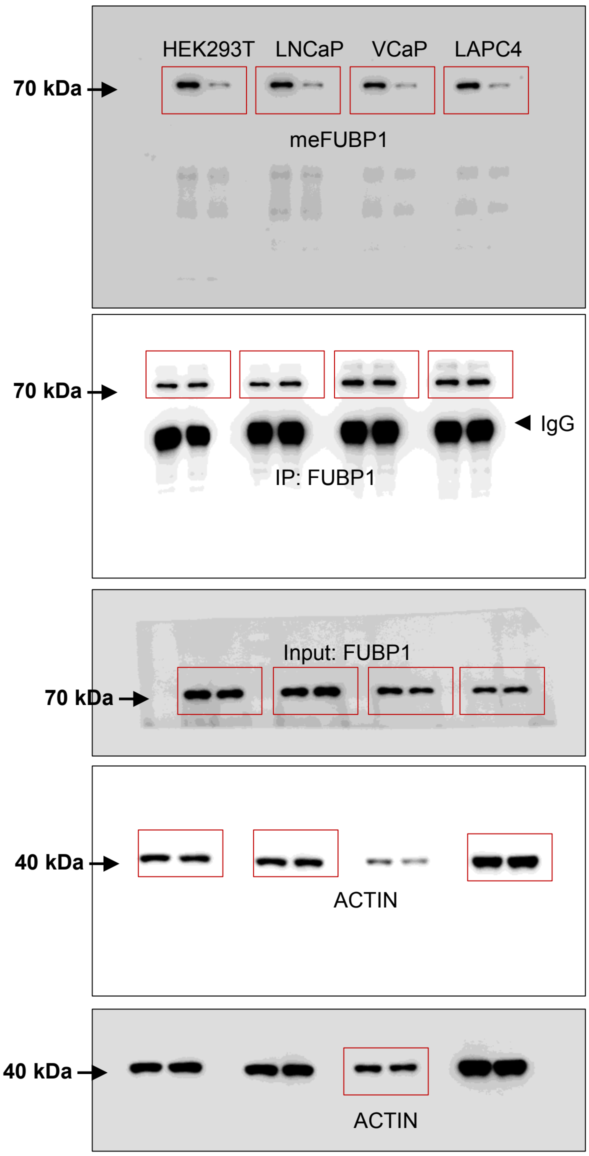
70 kDa
HEK293T LNCaP VCaP LAPC4
meFUBP1
70 kDa
IgG
IP: FUBP1
Input: FUBP1
70 kDa
40 kDa
ACTIN
40 kDa
ACTIN

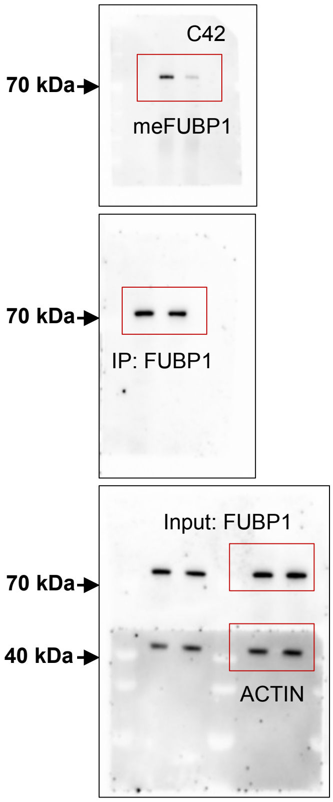
C42
70 kDa
meFUBP1
70 kDa
IP: FUBP1
Input: FUBP1
70 kDa
40 kDa
ACTIN

Figure 2A

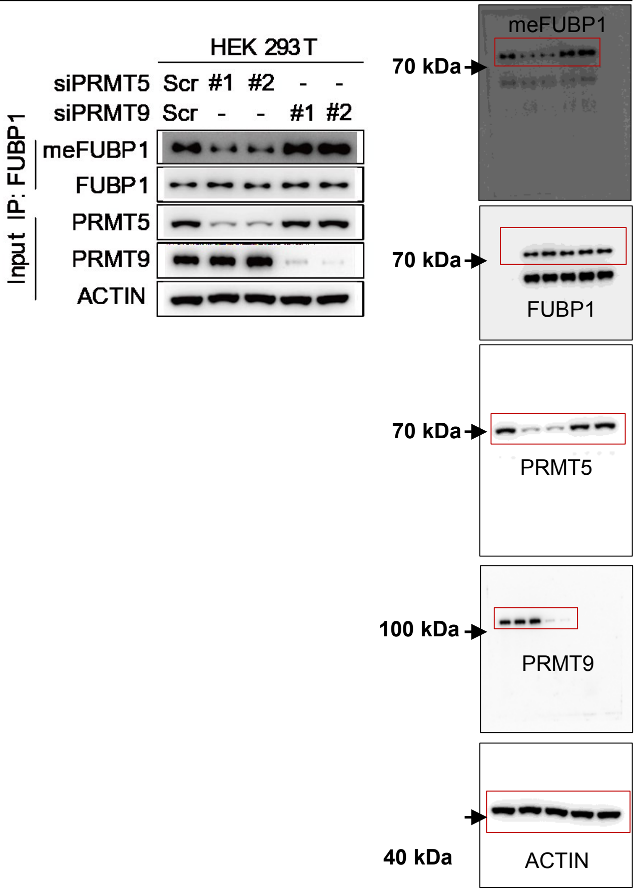

Figure 2B

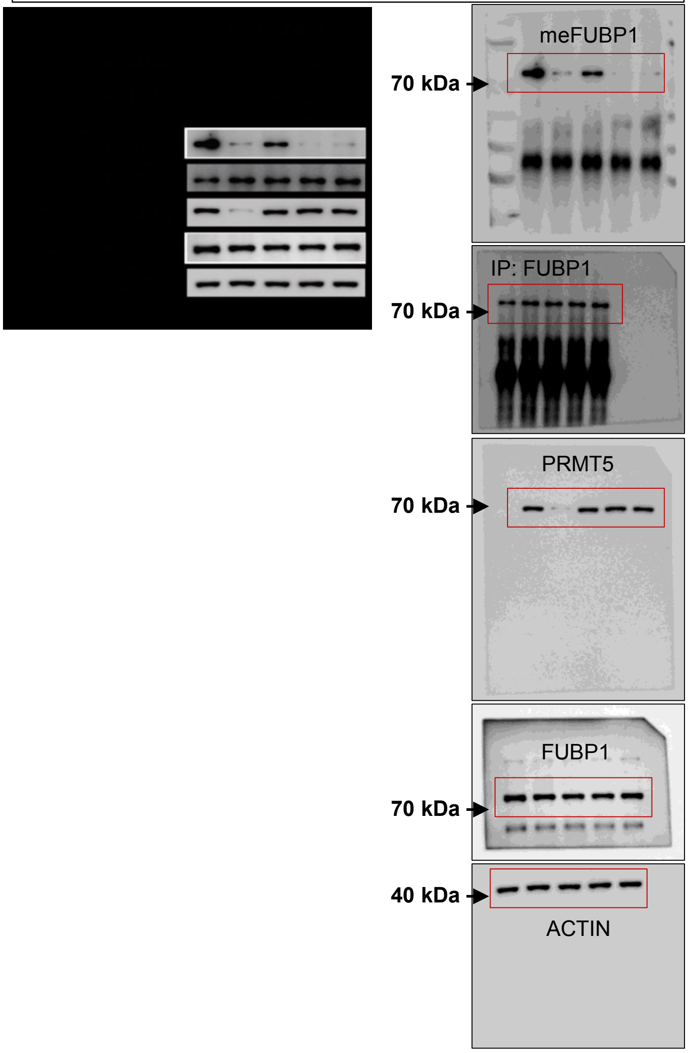

Figure 2C

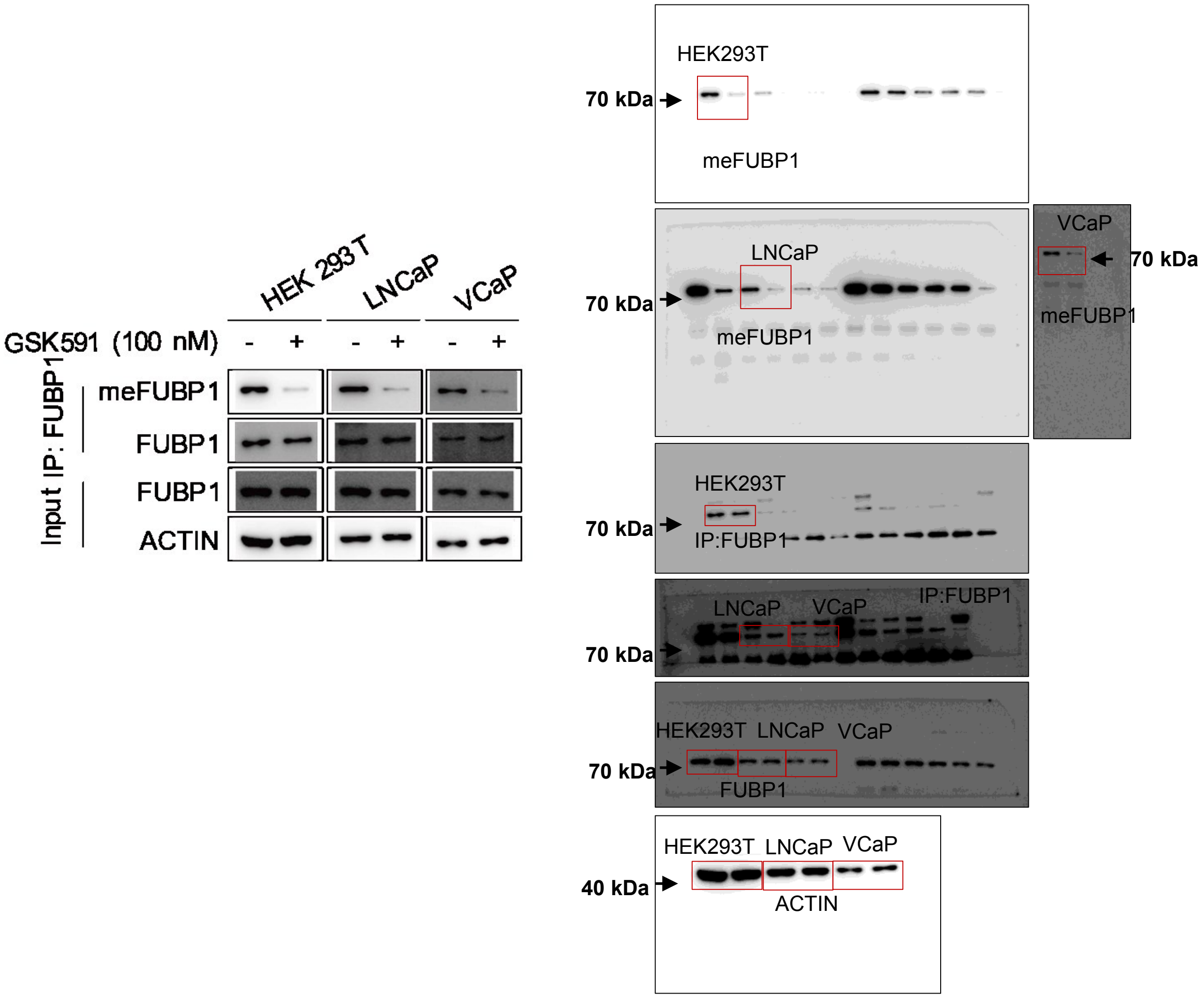

Figure 2D

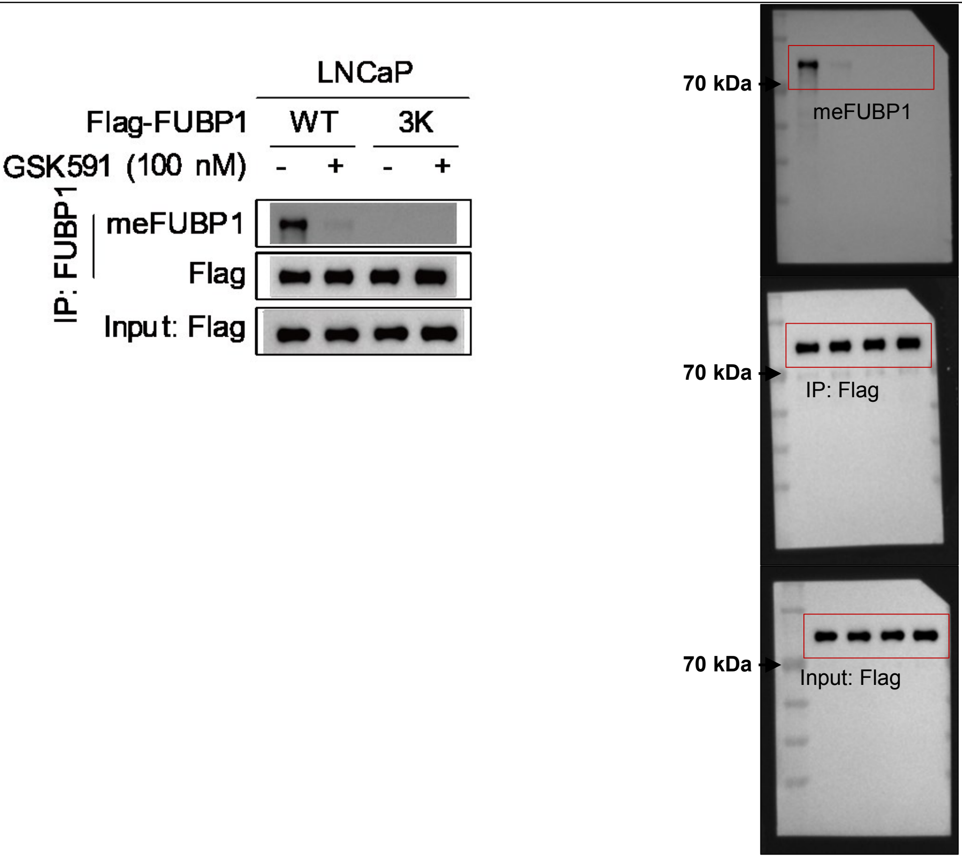

Figure 2E

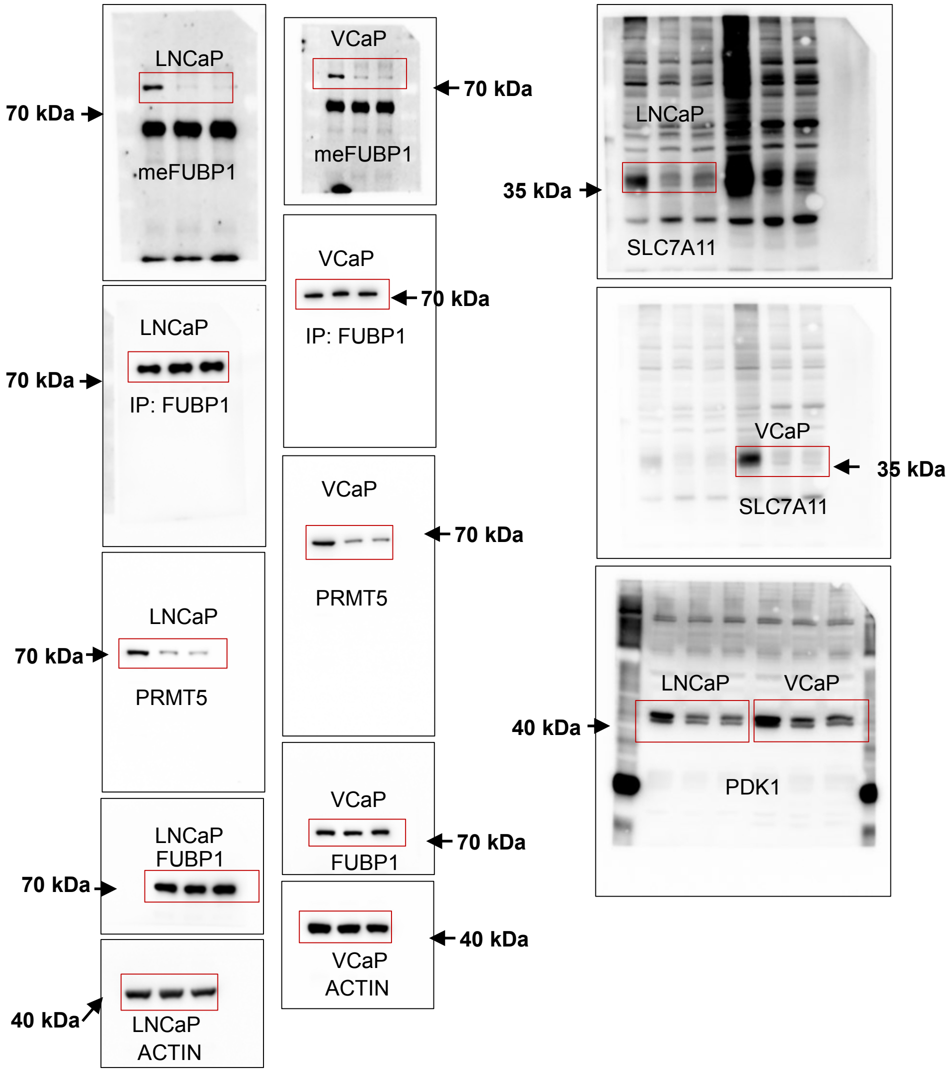
LNCaP
70 kDa
meFUBP1
VCaP
70 kDa
meFUBP1
LNCaP
35 kDa
SLC7A11
LNCaP
70 kDa
IP: FUBP1
VCaP
70 kDa
IP: FUBP1
VCaP
35 kDa
SLC7A11
VCaP
70 kDa
PRMT5
LNCaP
70 kDa
PRMT5
LNCaP
VCaP
40 kDa
PDK1
LNCaP
FUBP1
70 kDa
VCaP
70 kDa
FUBP1
40 kDa
VCaP
ACTIN
40 kDa
LNCaP
ACTIN

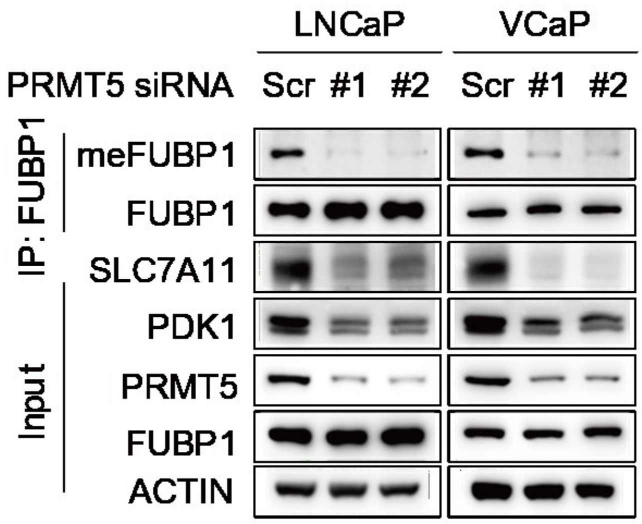
LNCaP
VCaP
PRMT5 siRNA
Scr #1 #2
Scr #1 #2
IP: FUBP1
meFUBP1
FUBP1
SLC7A11
Input
PDK1
PRMT5
FUBP1
ACTIN

Figure 2G

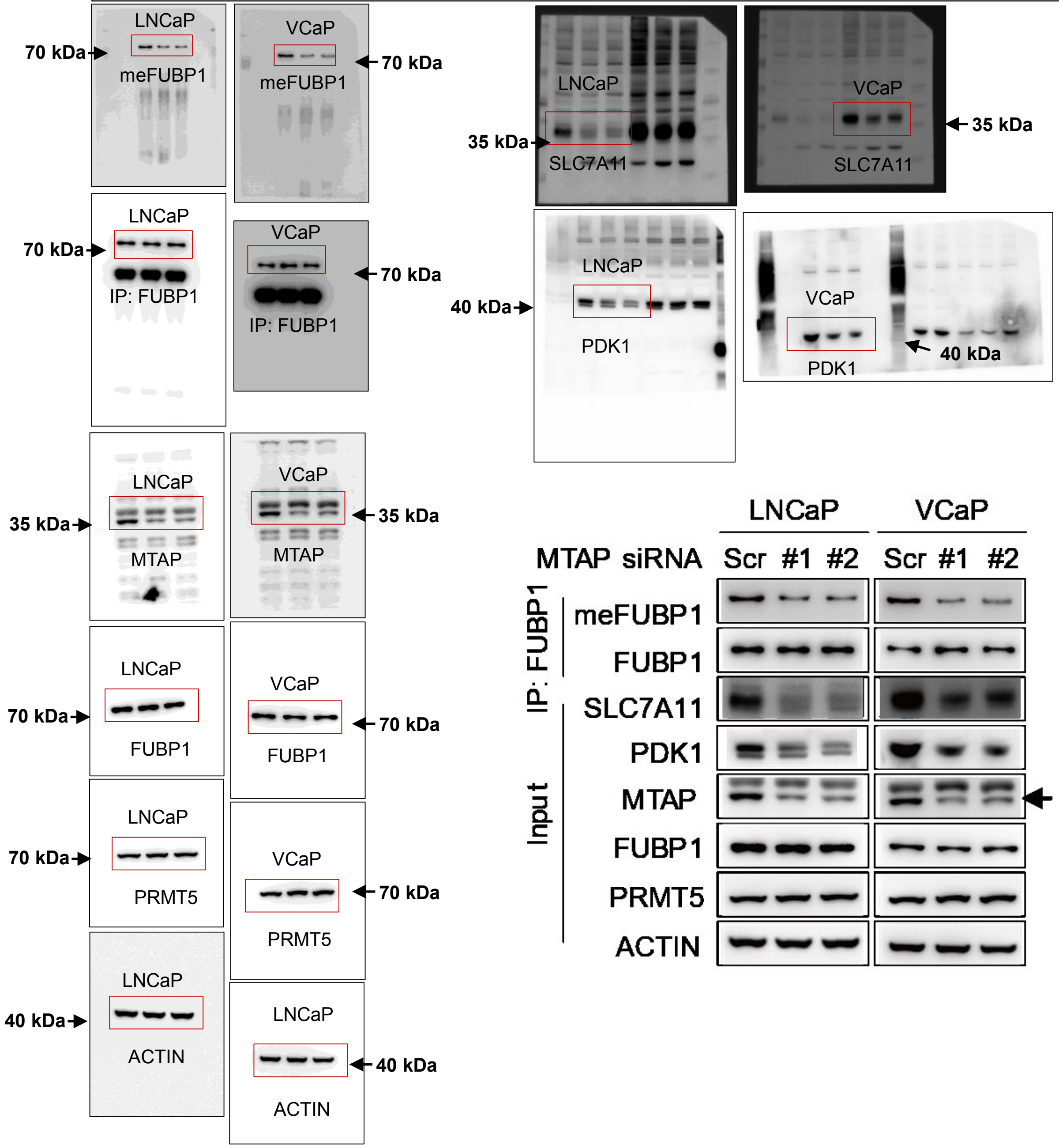
LNCaP
70 kDa
meFUBP1
VCaP
70 kDa
meFUBP1
LNCaP
70 kDa
IP: FUBP1
VCaP
70 kDa
IP: FUBP1
LNCaP
35 kDa
MTAP
VCaP
35 kDa
MTAP
LNCaP
70 kDa
FUBP1
VCaP
70 kDa
FUBP1
LNCaP
70 kDa
PRMT5
VCaP
70 kDa
PRMT5
LNCaP
40 kDa
ACTIN
LNCaP
40 kDa
ACTIN
LNCaP
35 kDa
SLC7A11
VCaP
35 kDa
SLC7A11
LNCaP
40 kDa
PDK1
VCaP
40 kDa
PDK1
LNCaP
VCaP
MTAP siRNA
Scr #1 #2
Scr #1 #2
IP: FUBP1
meFUBP1
FUBP1
Input
SLC7A11
PDK1
MTAP
FUBP1
PRMT5
ACTIN

Figure 2I

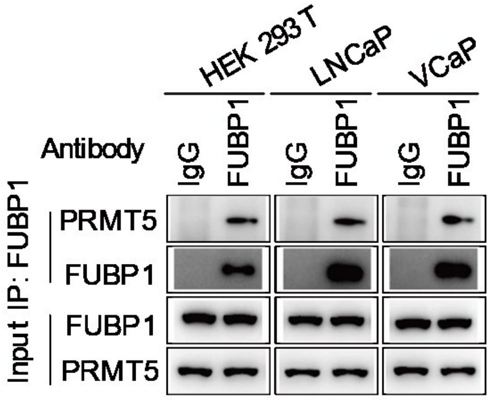

HEK 293 T
LNCaP
VCaP
Antibody
IgG
FUBP1
IgG
FUBP1
IgG
FUBP1
IP: FUBP1
Input
PRMT5
FUBP1
FUBP1
PRMT5

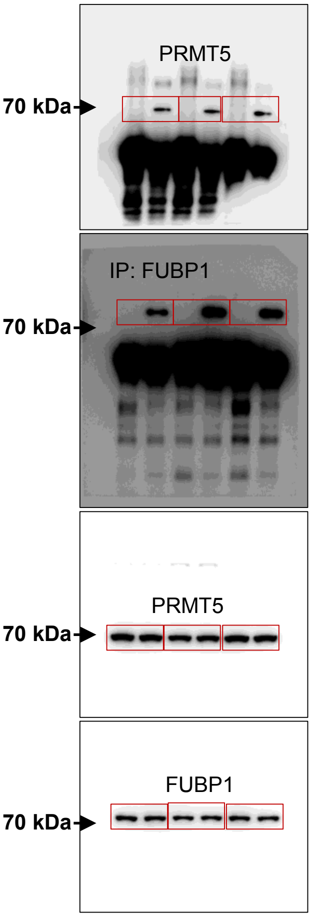

PRMT5
70 kDa
IP: FUBP1
70 kDa
PRMT5
70 kDa
FUBP1
70 kDa

Figure 2J

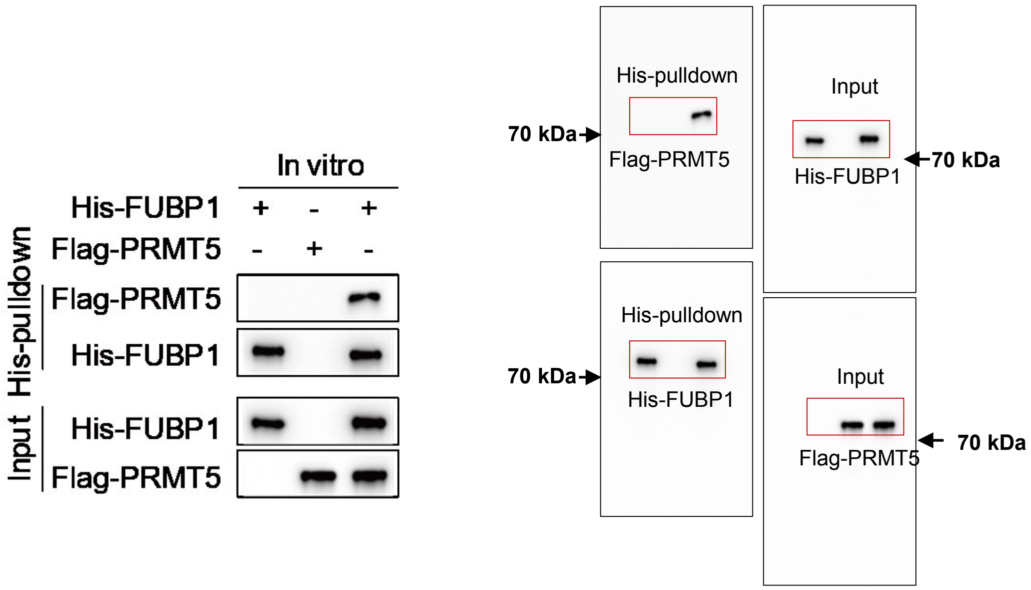

Figure 2K

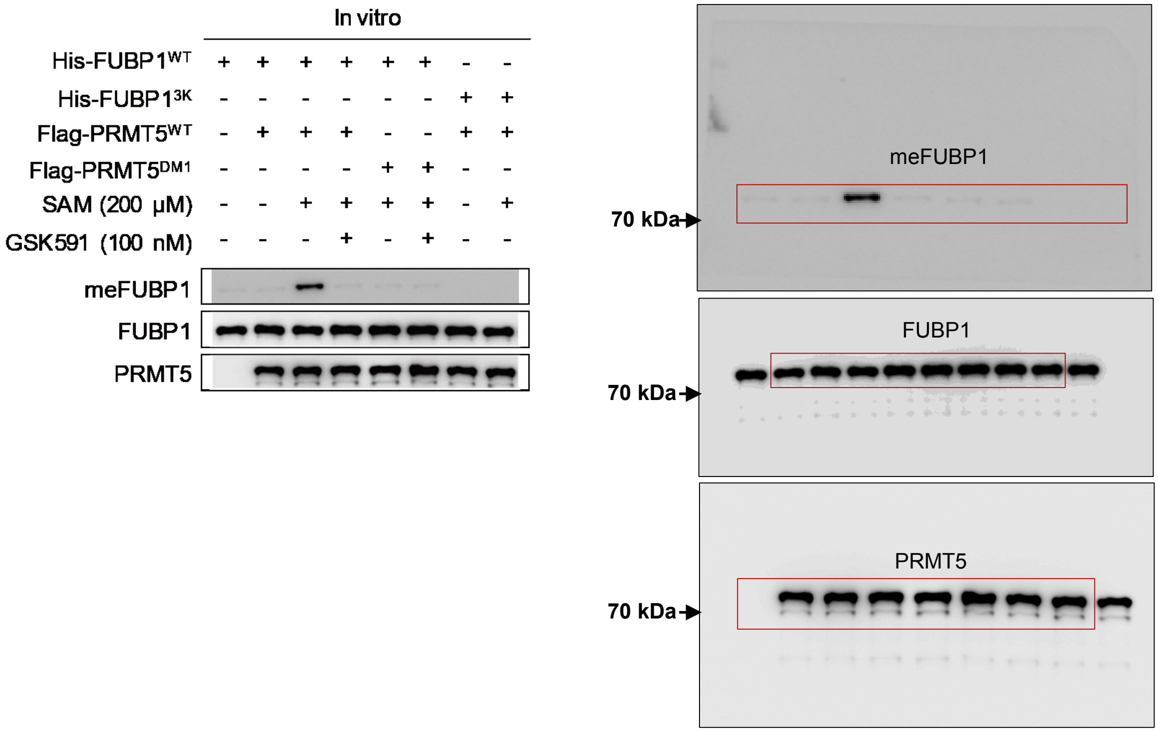

Figure 2L

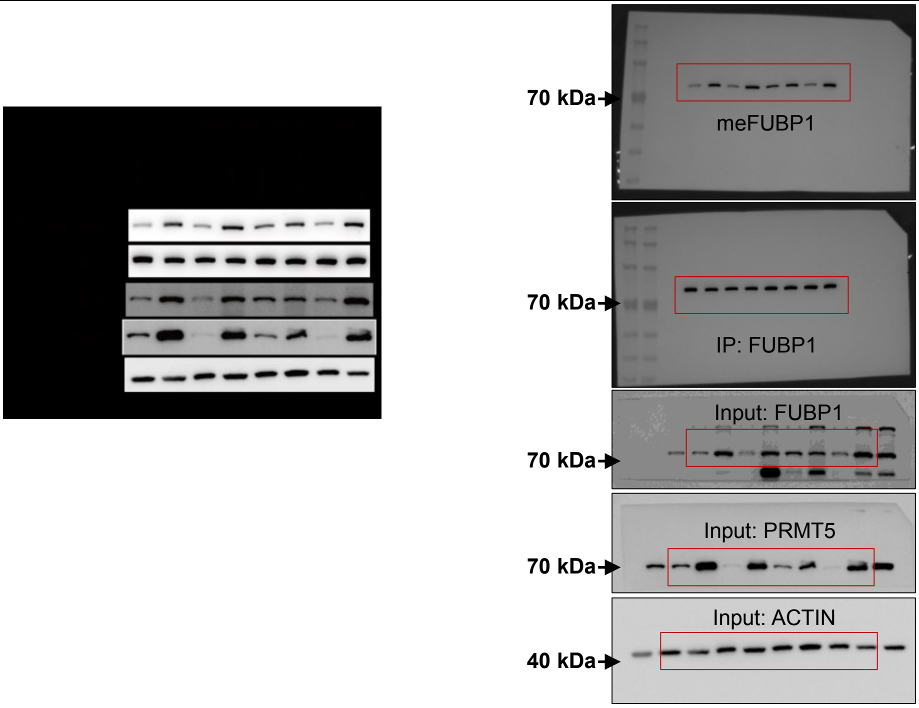

Figure 3A

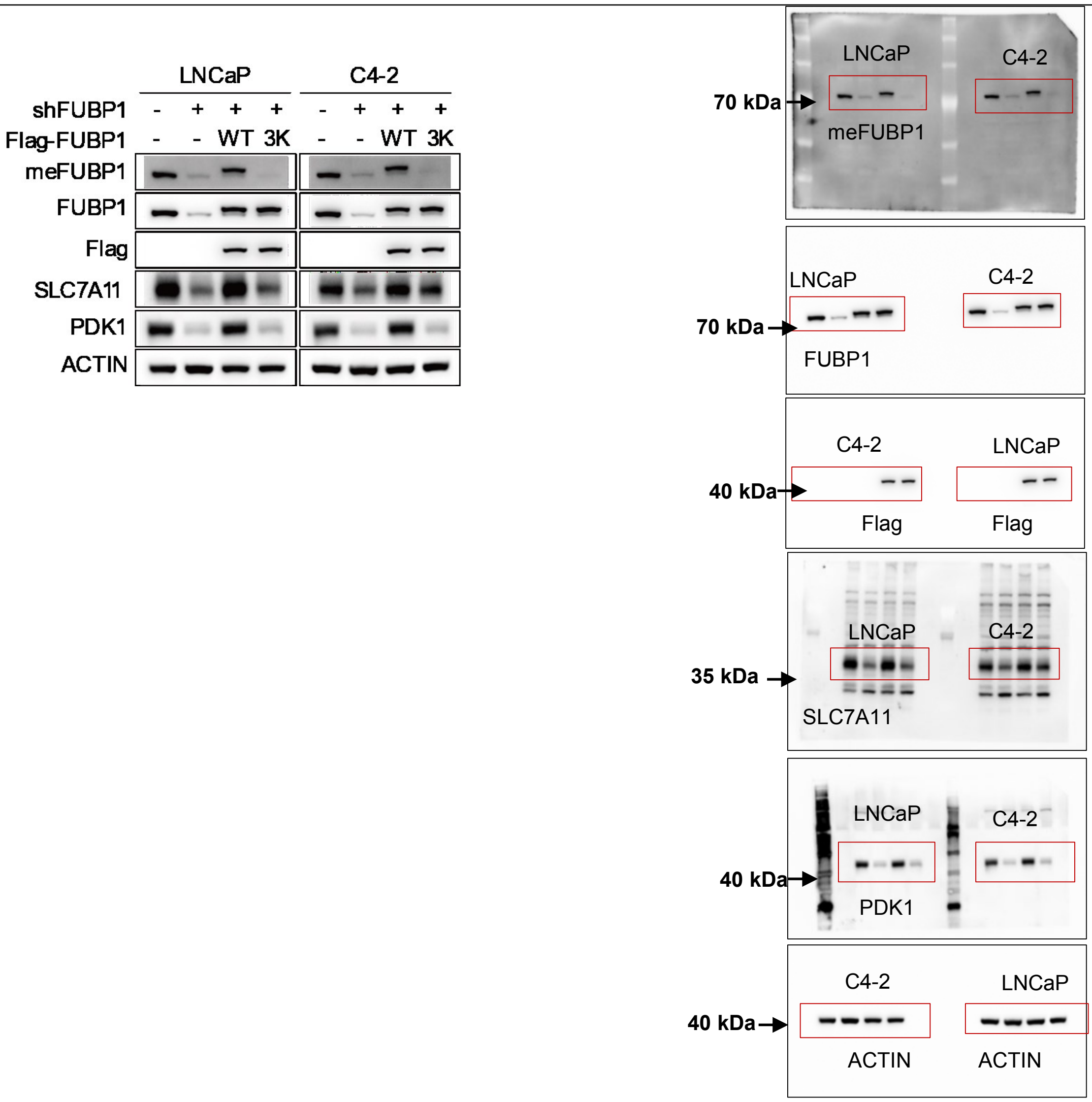

Figure 3G

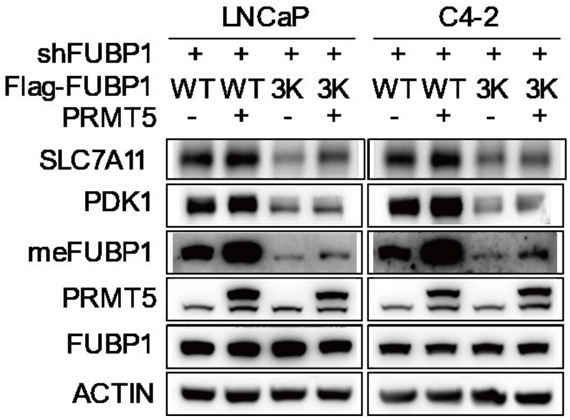
LNCaP
C4-2
shFUBP1 + + + + + + + +
Flag-FUBP1 WT WT 3K 3K WT WT 3K 3K
PRMT5 - + - + - + - +
SLC7A11
PDK1
meFUBP1
PRMT5
FUBP1
ACTIN

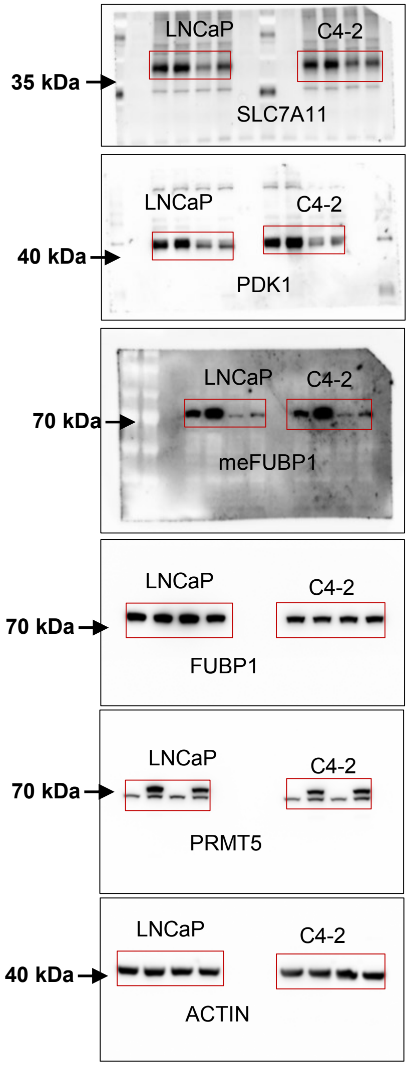
35 kDa
LNCaP
C4-2
SLC7A11
40 kDa
LNCaP
C4-2
PDK1
70 kDa
LNCaP
C4-2
meFUBP1
70 kDa
LNCaP
C4-2
FUBP1
70 kDa
LNCaP
C4-2
PRMT5
40 kDa
LNCaP
C4-2
ACTIN

Figure 3I

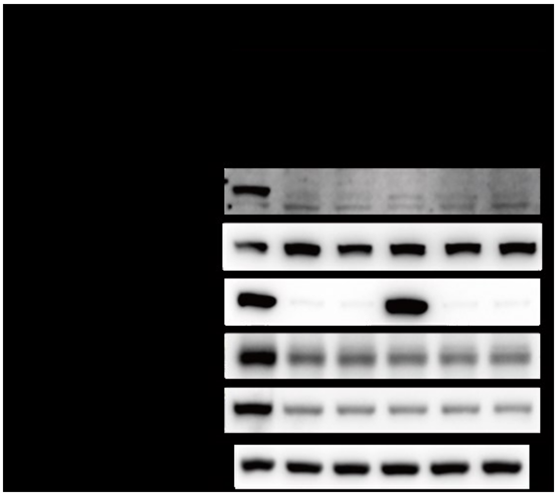

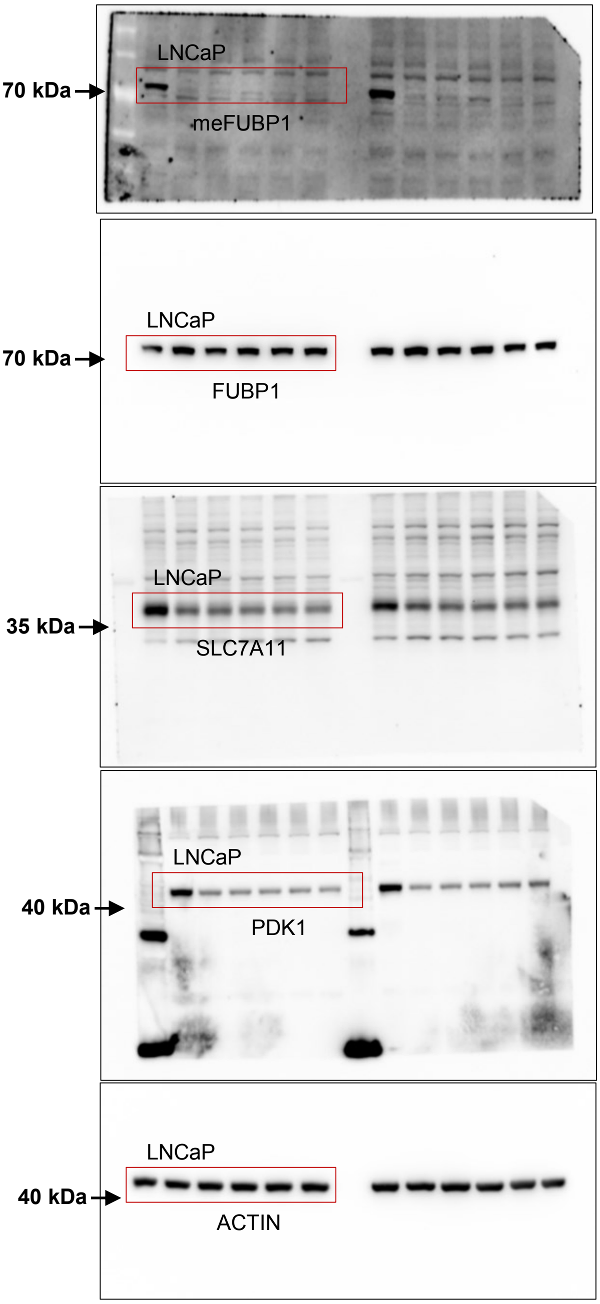

70 kDa
LNCaP
meFUBP1
70 kDa
LNCaP
FUBP1
35 kDa
LNCaP
SLC7A11
40 kDa
LNCaP
PDK1
40 kDa
LNCaP
ACTIN

Figure 6B

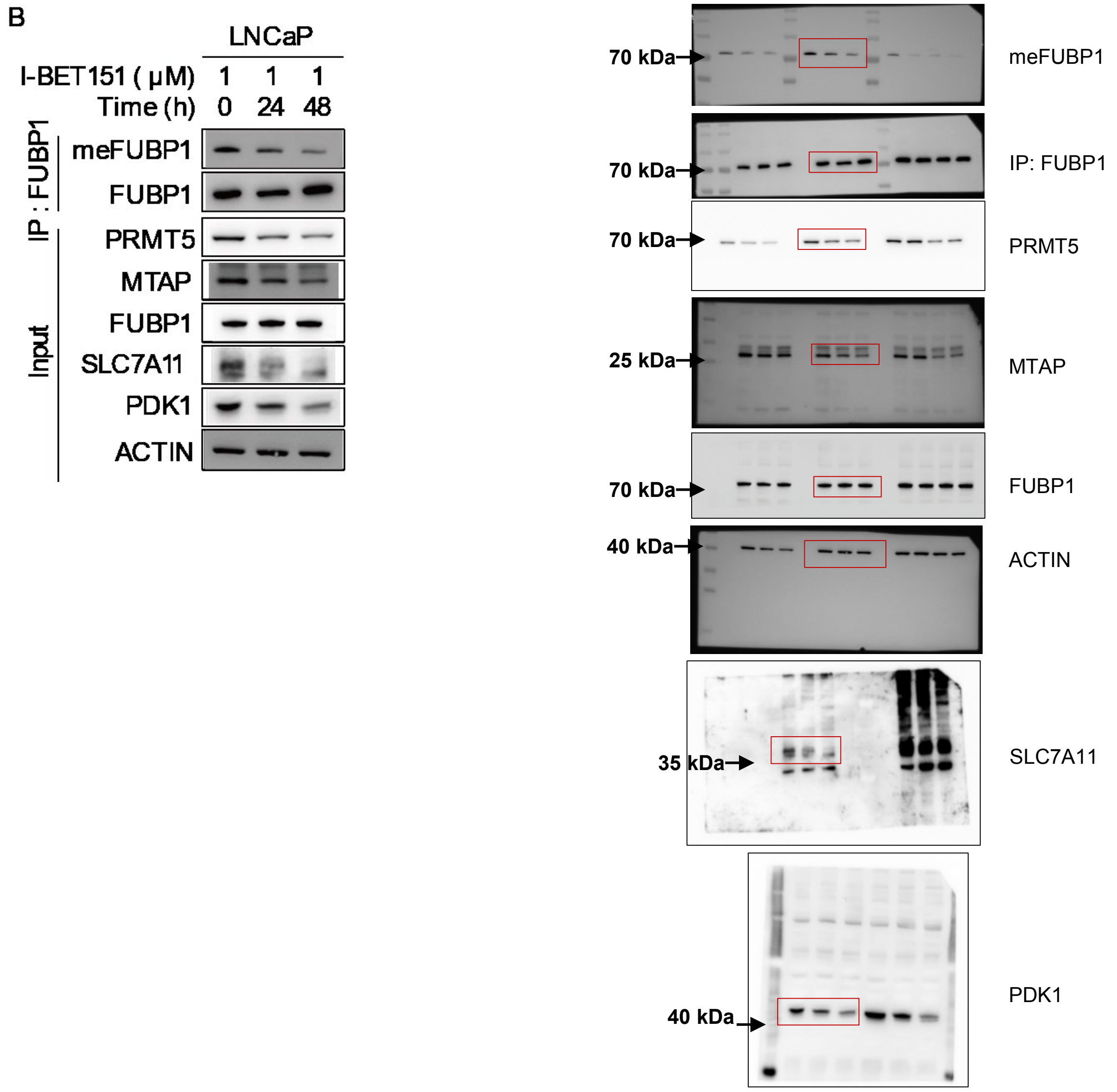
B
LNCaP
I-BET151 (μM) 1 1 1
Time (h) 0 24 48
IP : FUBP1
meFUBP1
FUBP1
Input
PRMT5
MTAP
FUBP1
SLC7A11
PDK1
ACTIN
70 kDa
meFUBP1
70 kDa
IP: FUBP1
70 kDa
PRMT5
25 kDa
MTAP
70 kDa
FUBP1
40 kDa
ACTIN
35 kDa
SLC7A11
40 kDa
PDK1

Figure 6E

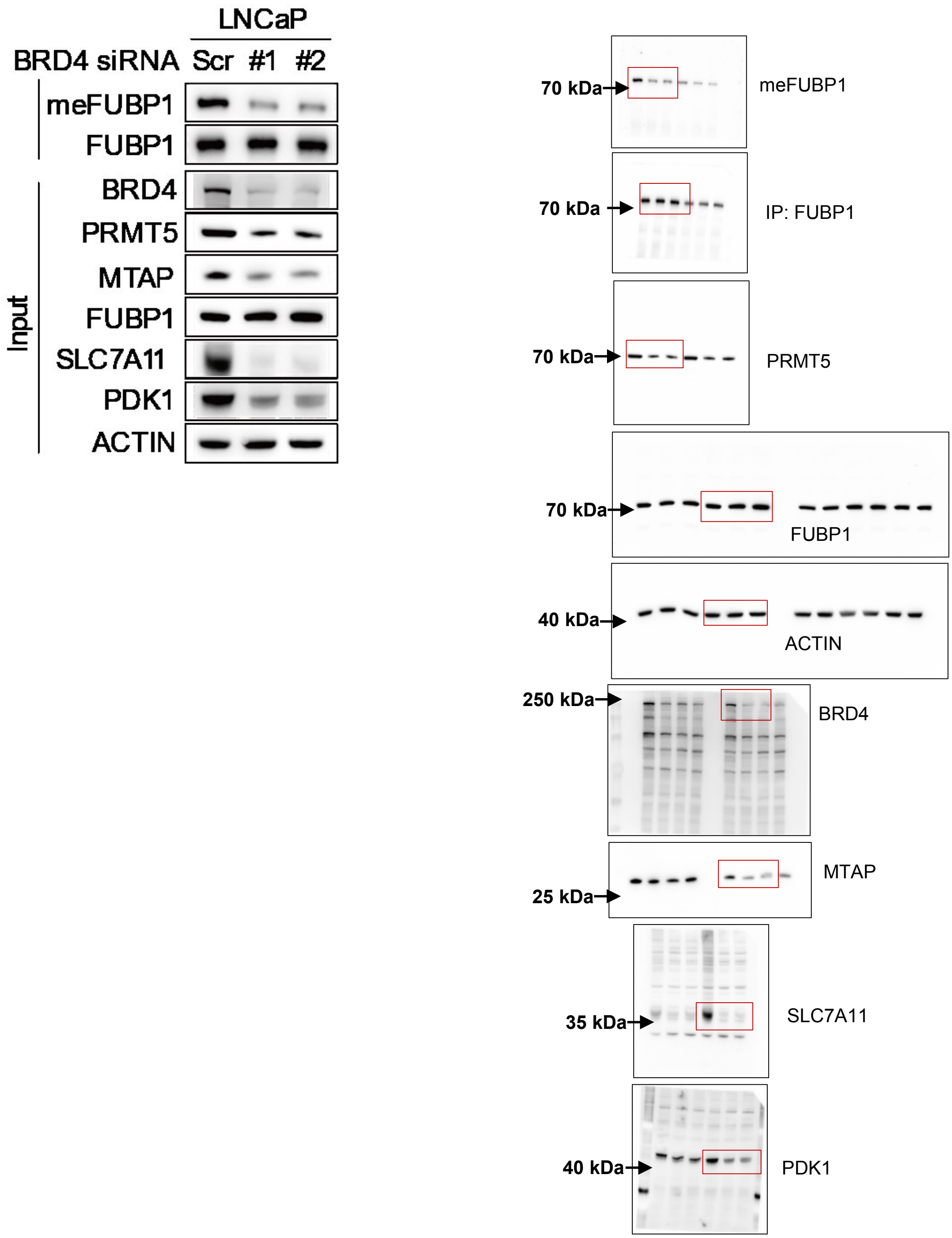
LNCaP
BRD4 siRNA Scr #1 #2
meFUBP1
FUBP1
BRD4
PRMT5
MTAP
FUBP1
SLC7A11
PDK1
ACTIN
Input
70 kDa
meFUBP1
70 kDa
IP: FUBP1
70 kDa
PRMT5
70 kDa
FUBP1
40 kDa
ACTIN
250 kDa
BRD4
MTAP
25 kDa
35 kDa
SLC7A11
40 kDa
PDK1

Figure 6H

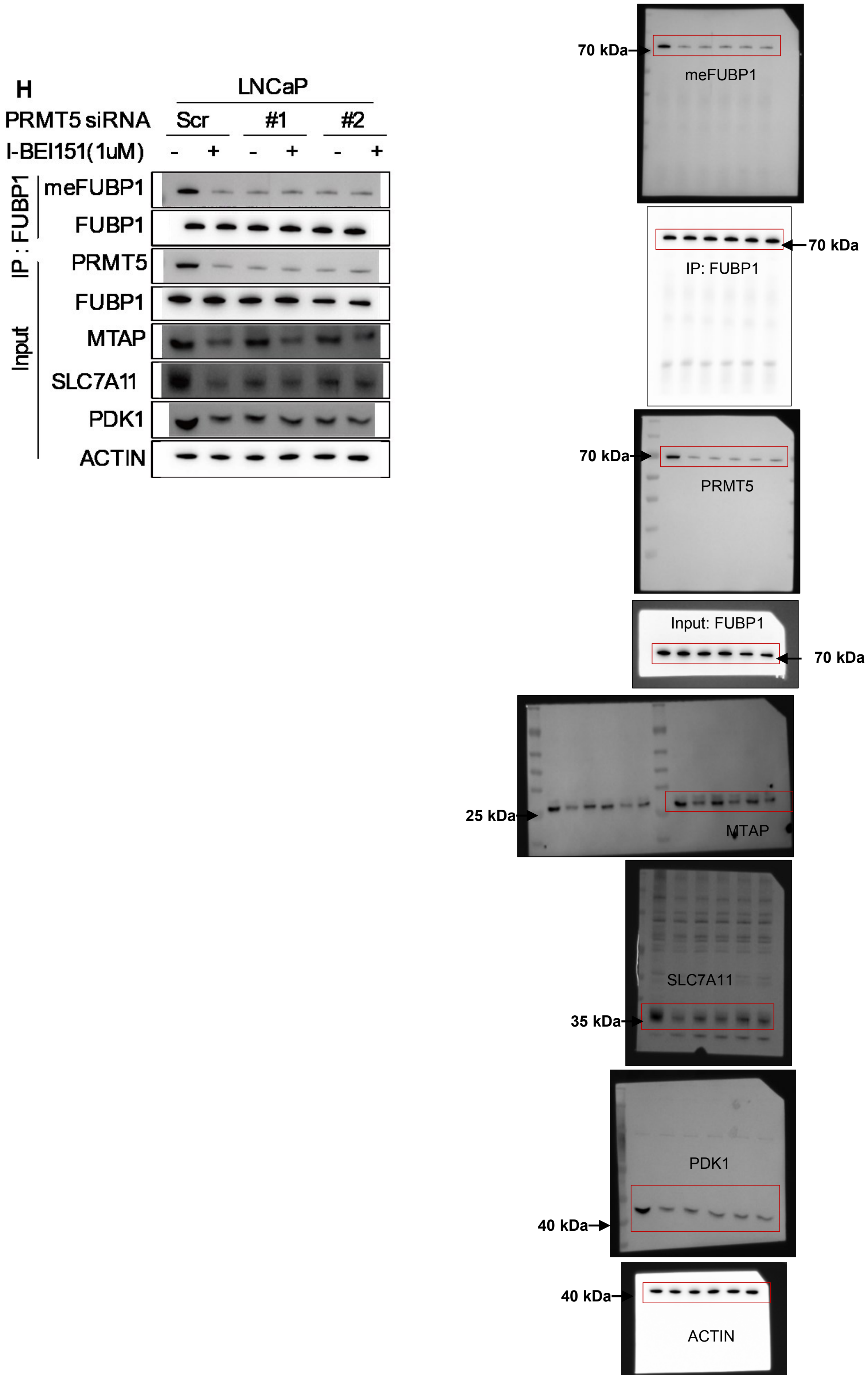

H
LNCaP
PRMT5 siRNA
Scr
#1
#2
I-BEI151(1uM)
- + - + - +
IP : FUBP1
Input
meFUBP1
FUBP1
PRMT5
FUBP1
MTAP
SLC7A11
PDK1
ACTIN
70 kDa
meFUBP1
70 kDa
IP: FUBP1
70 kDa
PRMT5
Input: FUBP1
70 kDa
25 kDa
MTAP
SLC7A11
35 kDa
PDK1
40 kDa
40 kDa
ACTIN

Figure 6I

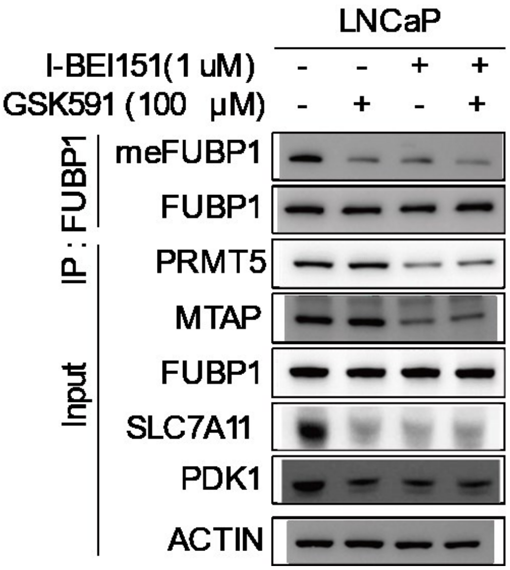

LNCaP
I-BEI151(1 uM) - - + +
GSK591 (100 μM) - + - +
IP : FUBP1
meFUBP1
FUBP1
Input
PRMT5
MTAP
FUBP1
SLC7A11
PDK1
ACTIN

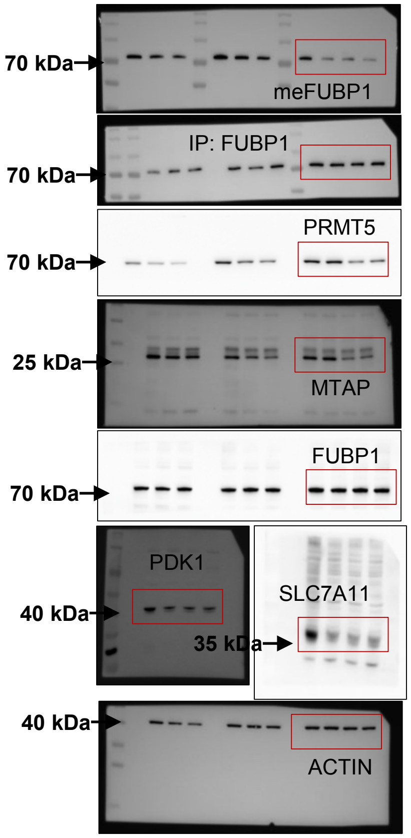

70 kDa
meFUBP1
IP: FUBP1
70 kDa
PRMT5
70 kDa
25 kDa
MTAP
FUBP1
70 kDa
PDK1
40 kDa
SLC7A11
35 kDa
40 kDa
ACTIN

Figure 9A

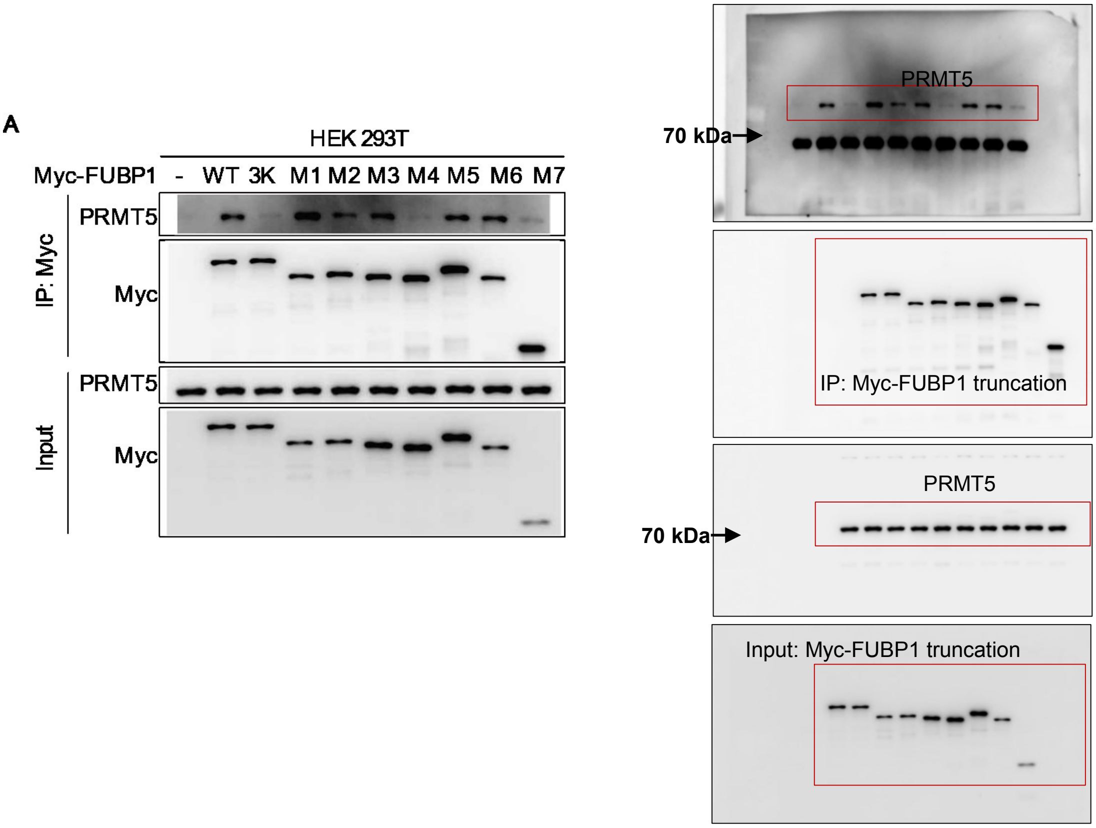

Figure 9B

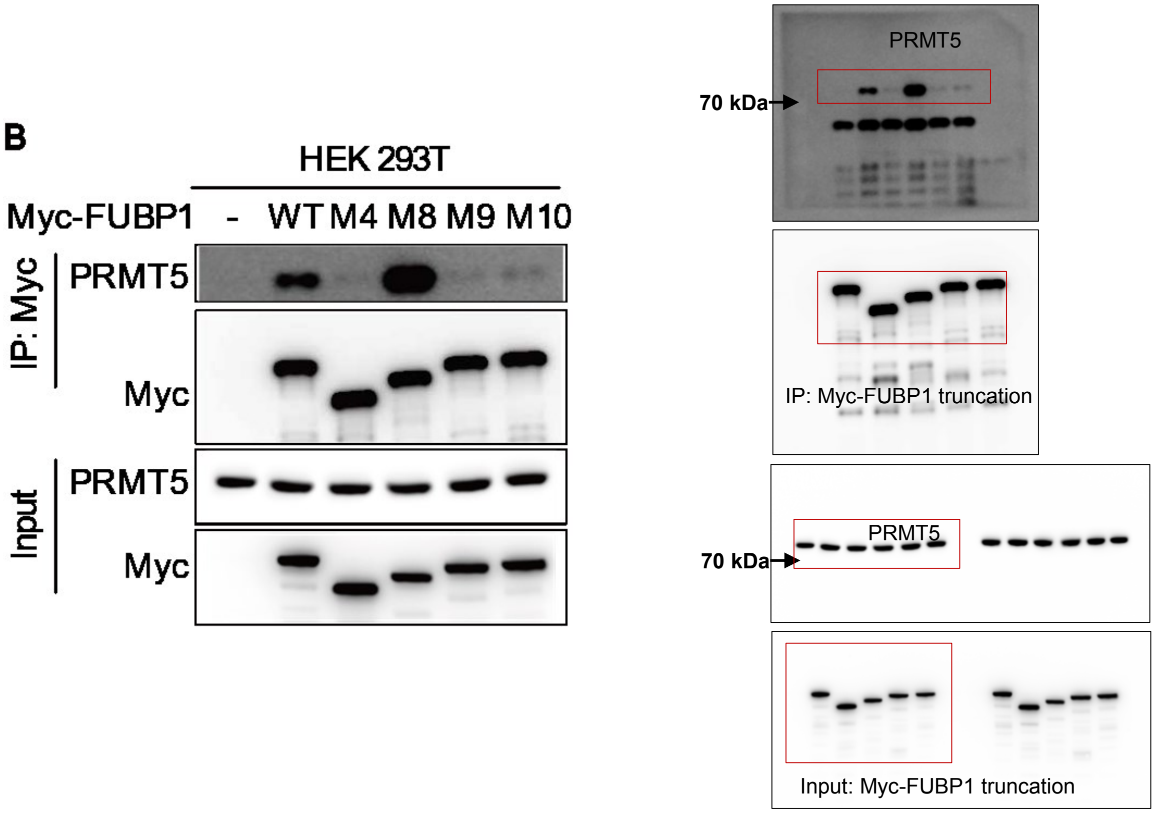

Figure 9D

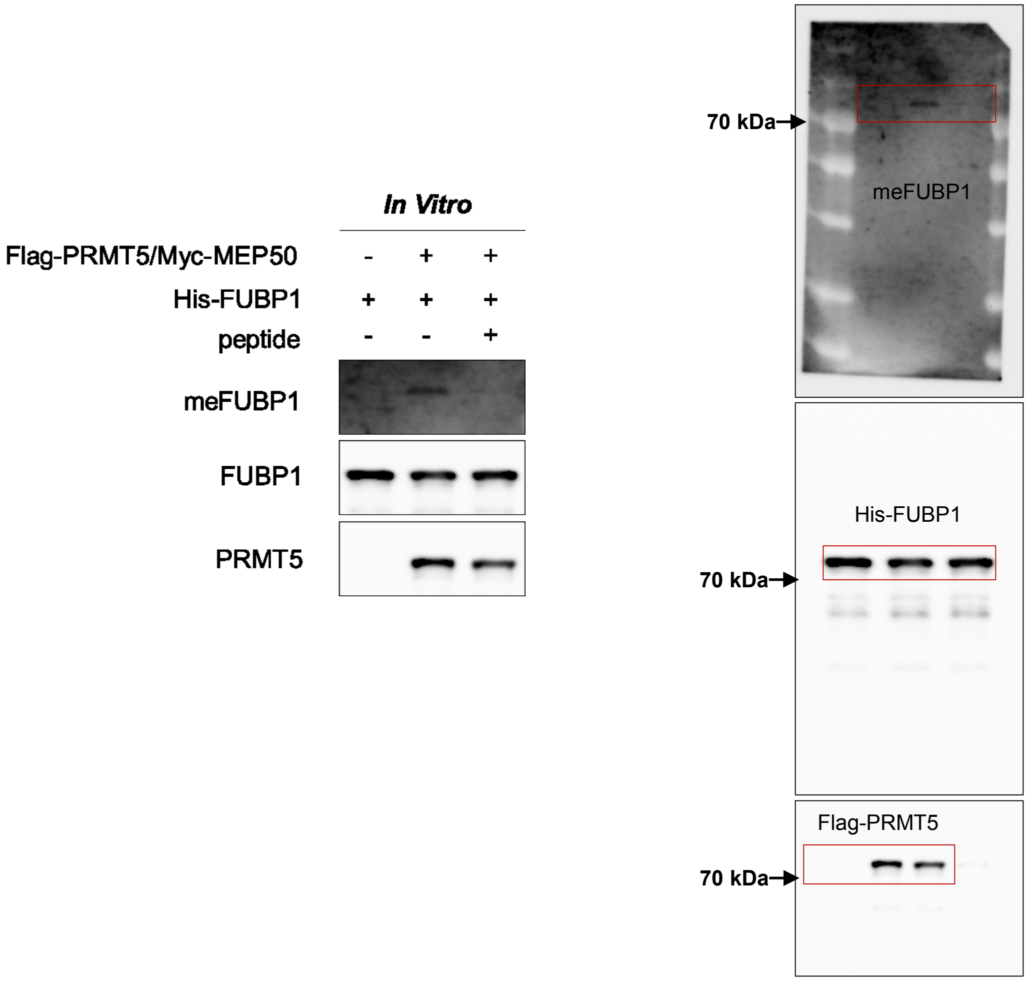

Figure 9E

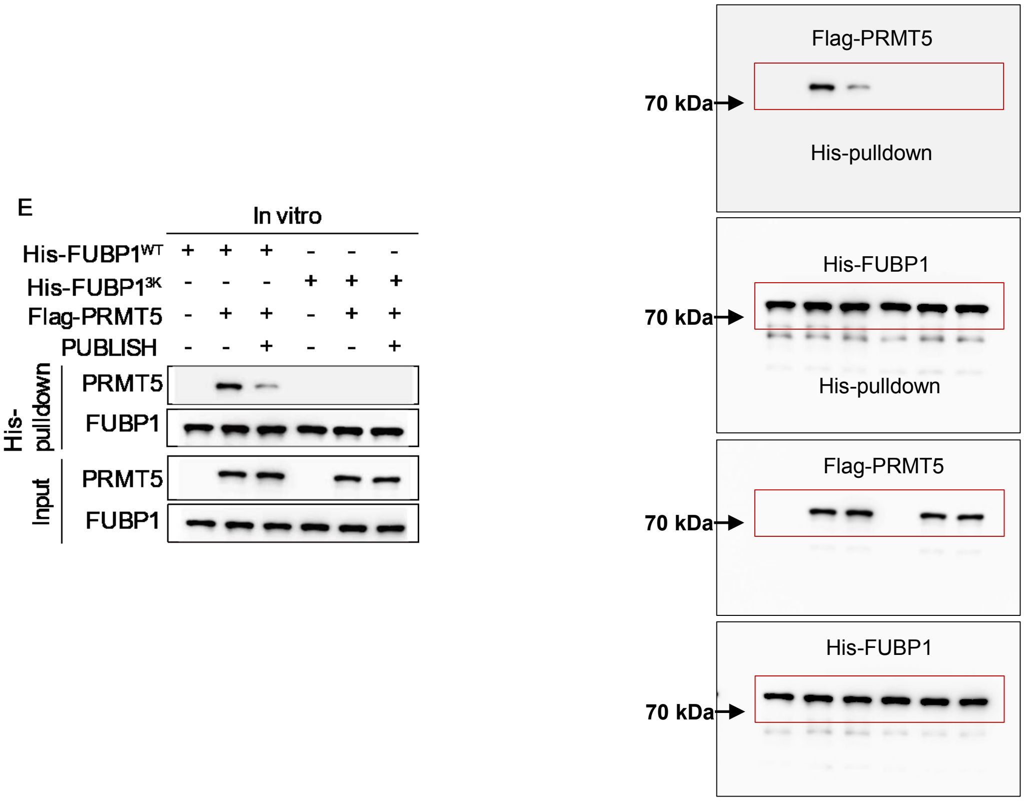

Figure 9G

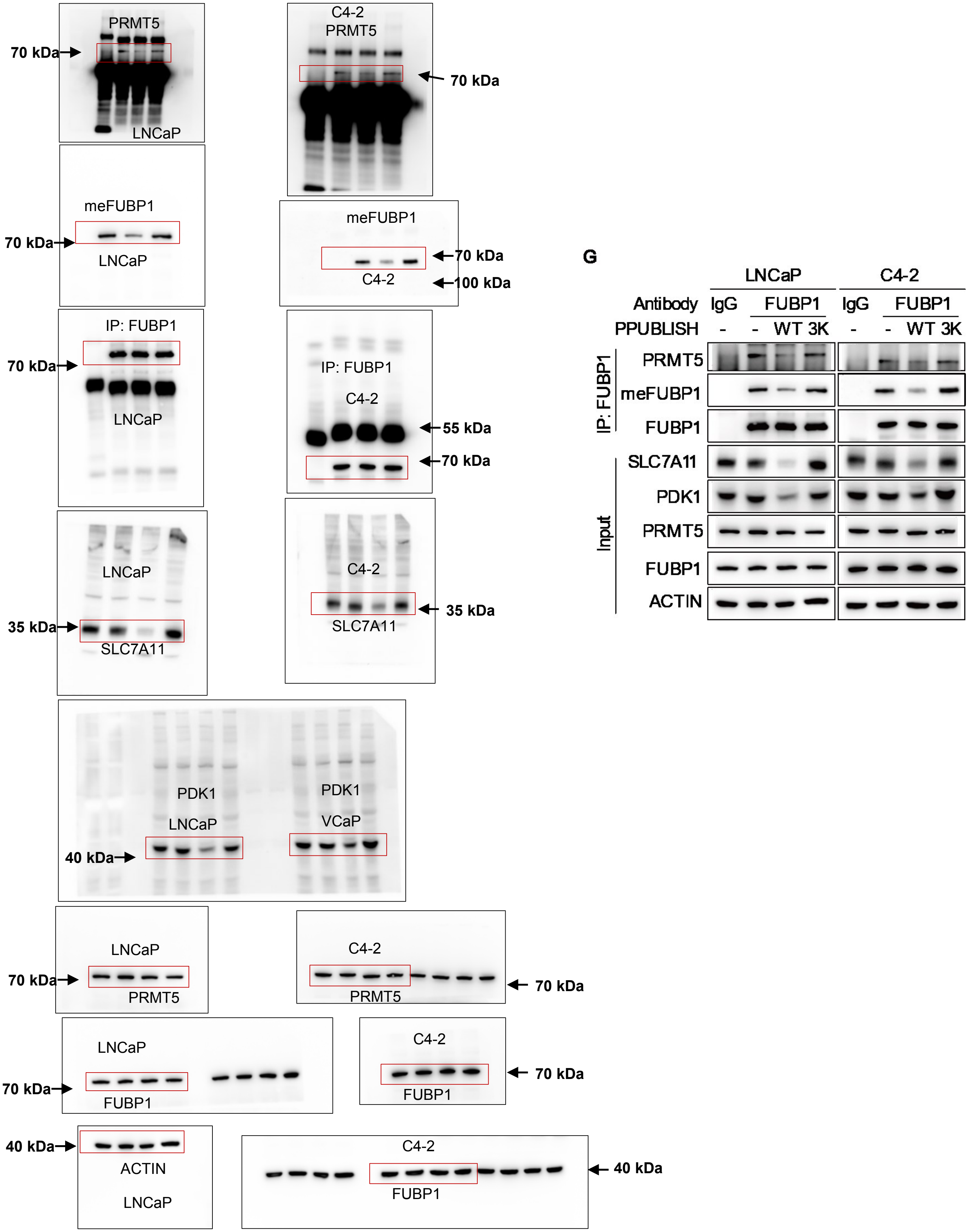
PRMT5
70 kDa
LNCaP
C4-2
PRMT5
70 kDa
meFUBP1
70 kDa
LNCaP
meFUBP1
70 kDa
C4-2
100 kDa
IP: FUBP1
70 kDa
LNCaP
IP: FUBP1
C4-2
55 kDa
70 kDa
LNCaP
35 kDa
SLC7A11
C4-2
35 kDa
SLC7A11
PDK1
LNCaP
40 kDa
PDK1
VCaP
LNCaP
70 kDa
PRMT5
C4-2
70 kDa
PRMT5
LNCaP
70 kDa
FUBP1
C4-2
70 kDa
FUBP1
40 kDa
ACTIN
LNCaP
C4-2
40 kDa
FUBP1
G
LNCaP
C4-2
Antibody
IgG
FUBP1
IgG
FUBP1
PPUBLISH
-
-
WT
3K
-
-
WT
3K
IP: FUBP1
PRMT5
meFUBP1
FUBP1
Input
SLC7A11
PDK1
PRMT5
FUBP1
ACTIN

Figure S1I

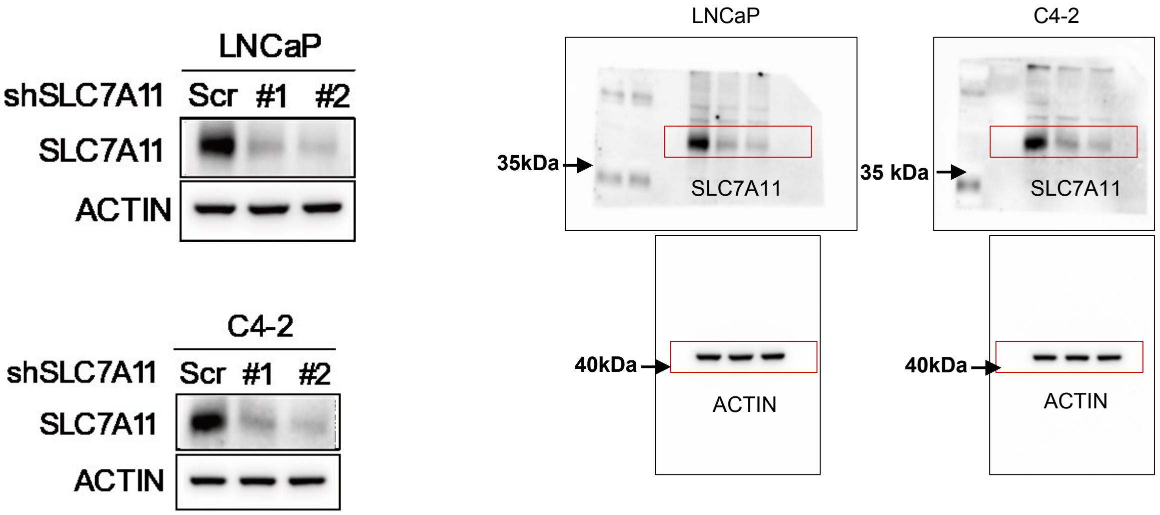

Figure S1J

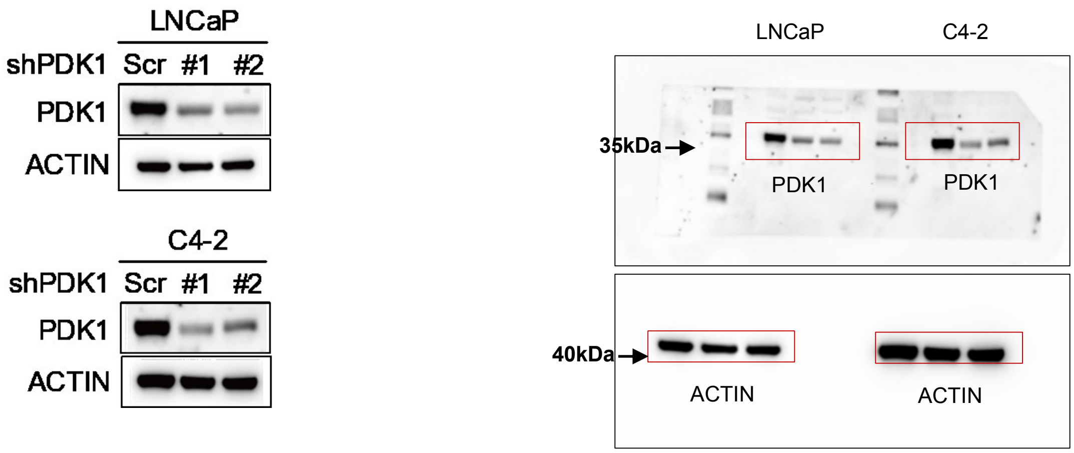

Figure S1K

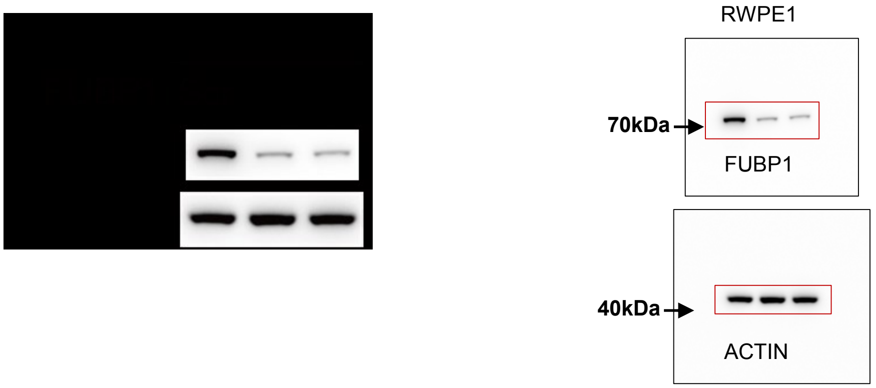

Figure S2B

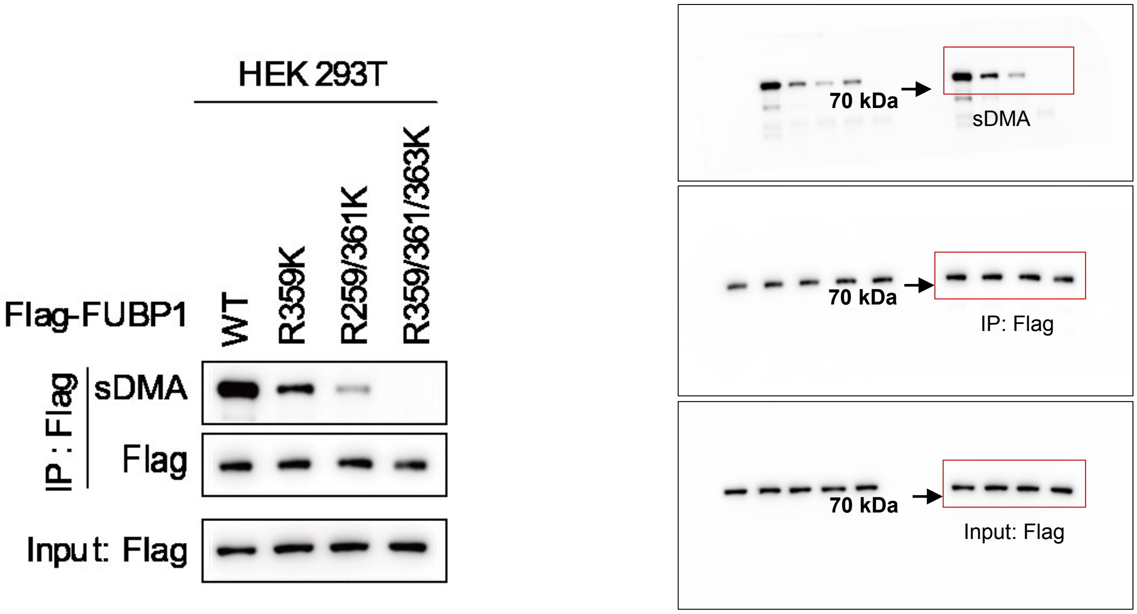

Figure S2C

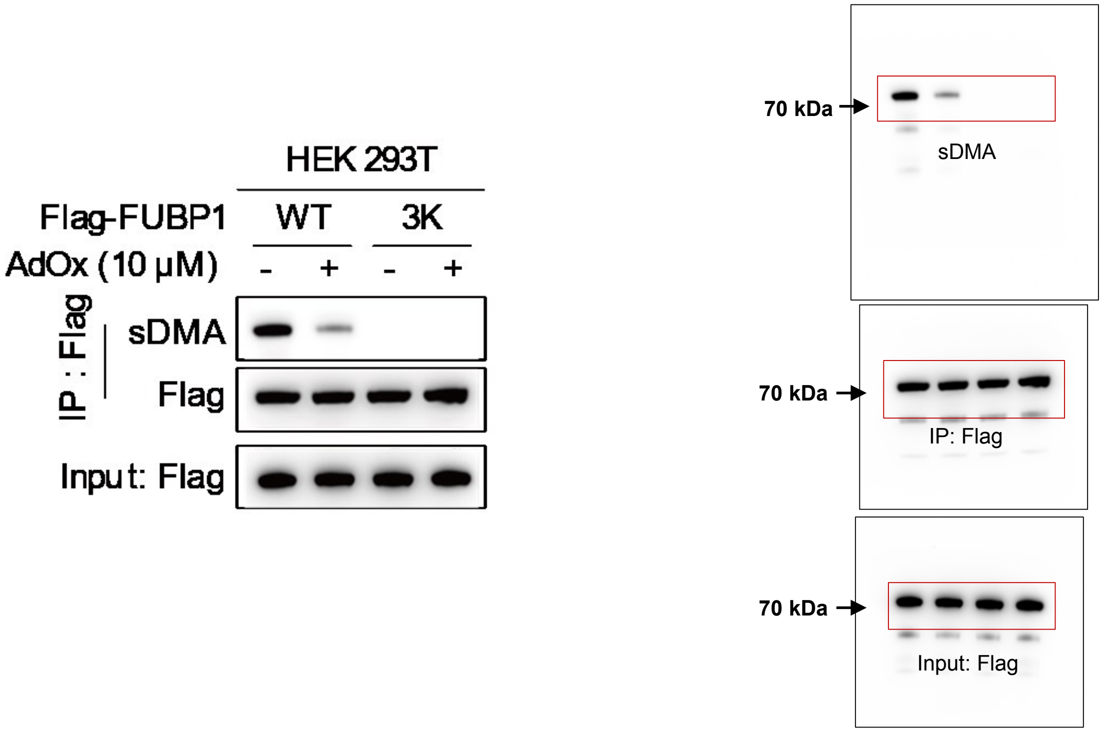

Figure S2D

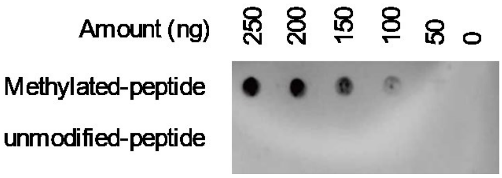

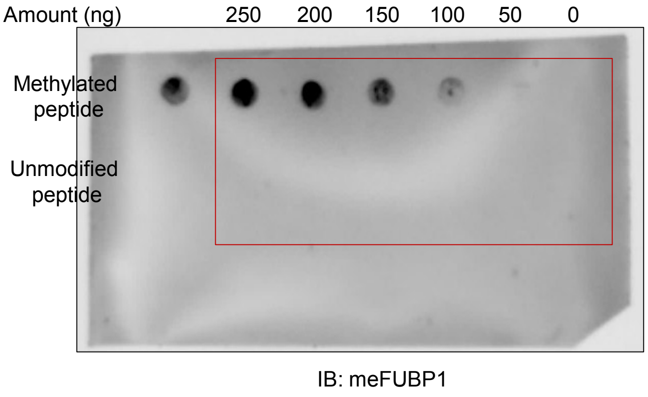

Figure S2E

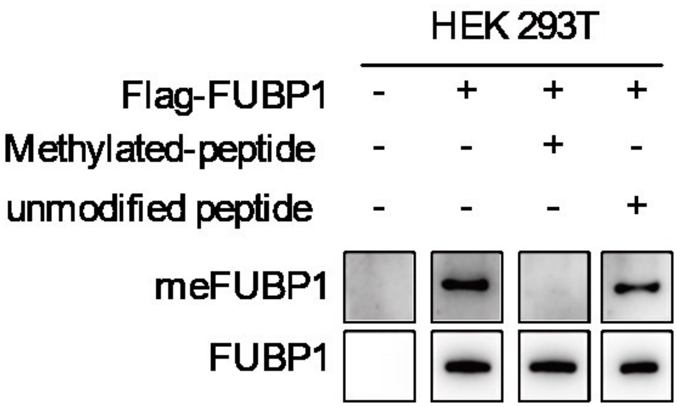

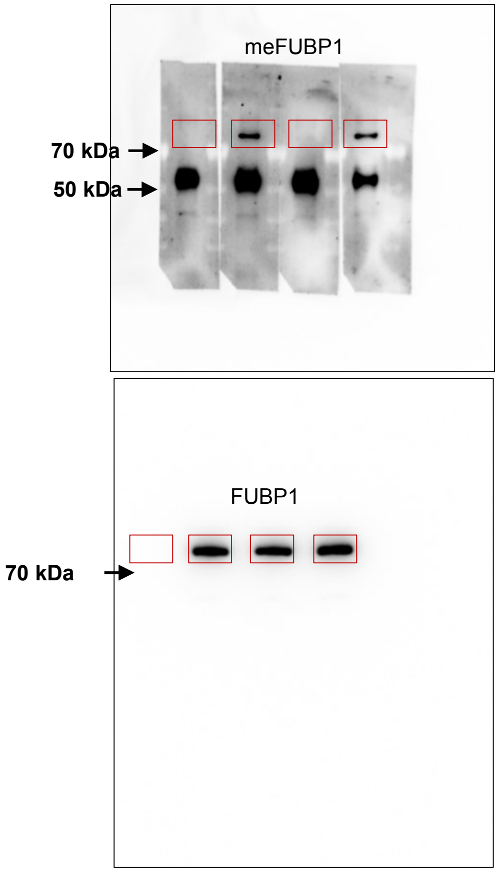

Figure S2F

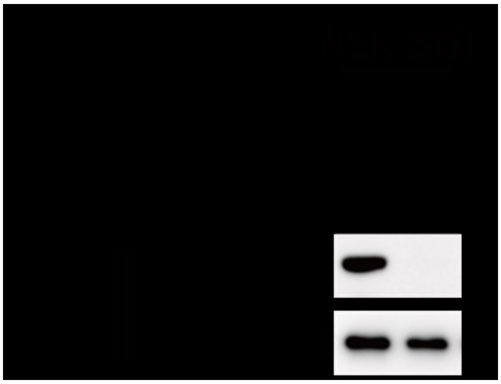

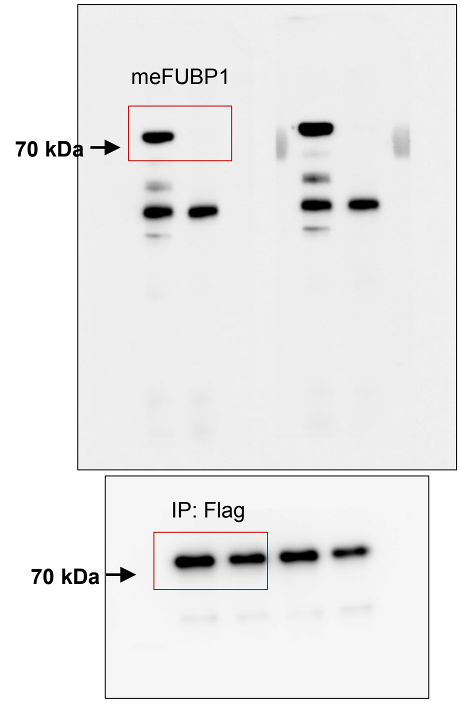

Figure S2G

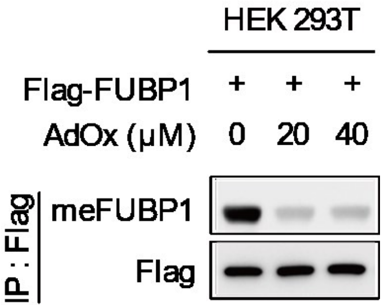

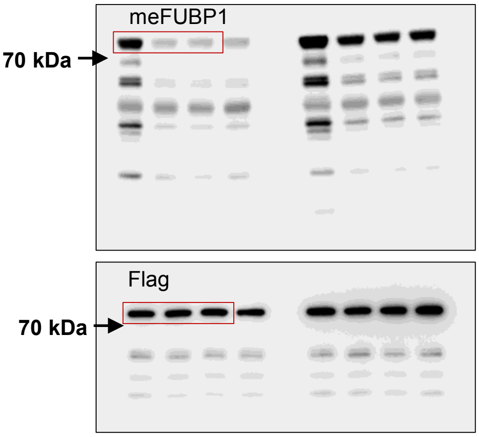

Figure S3A

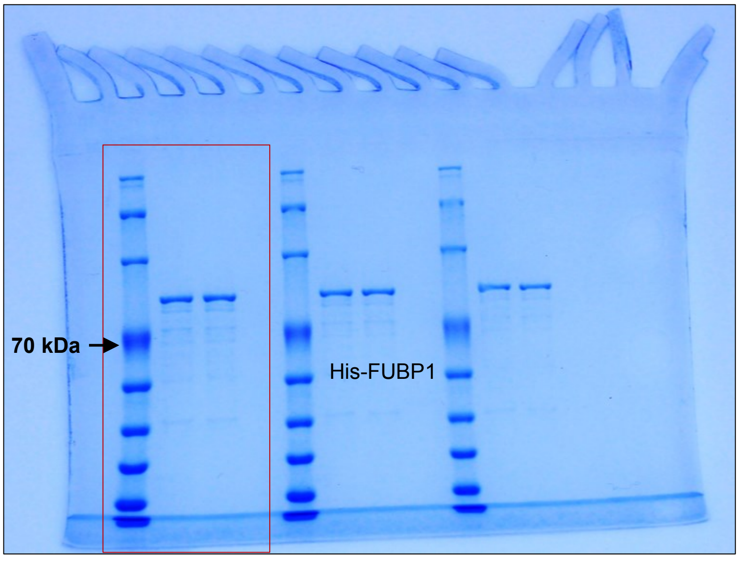

Figure S3B

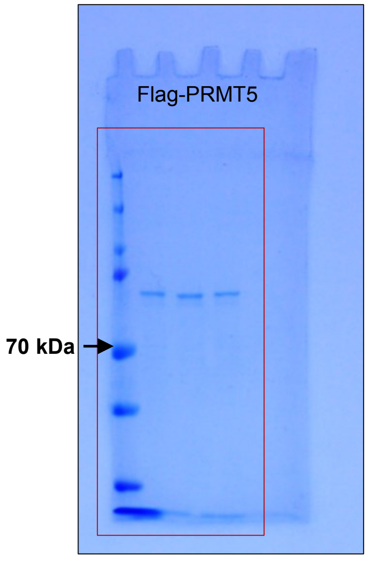

Figure S4

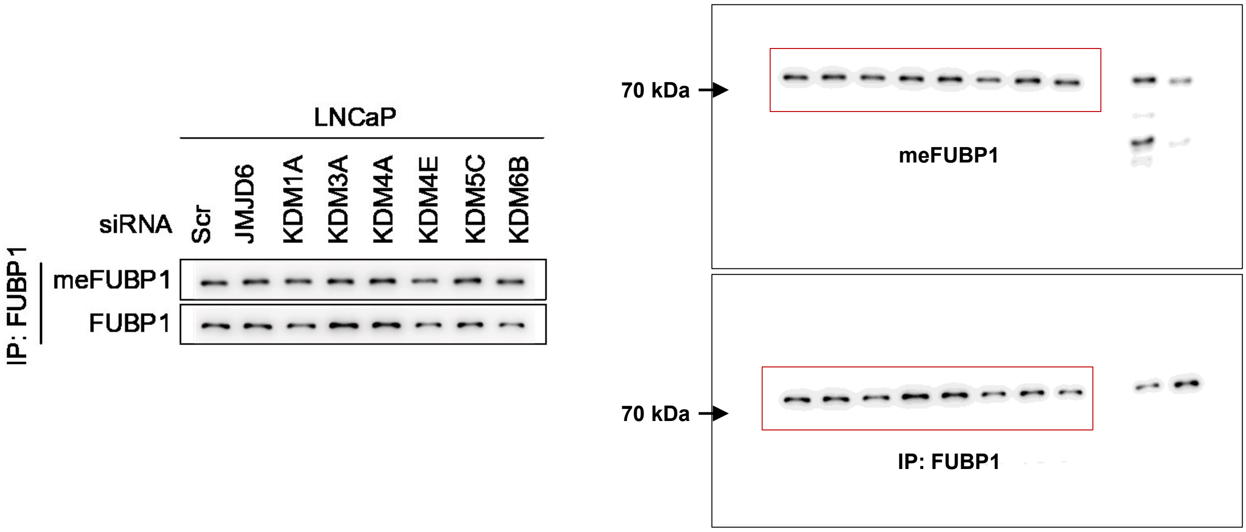

LNCaP
siRNA
Scr
JMJD6
KDM1A
KDM3A
KDM4A
KDM4E
KDM5C
KDM6B
IP: FUBP1
meFUBP1
FUBP1
70 kDa
meFUBP1
70 kDa
IP: FUBP1

Figure S6A

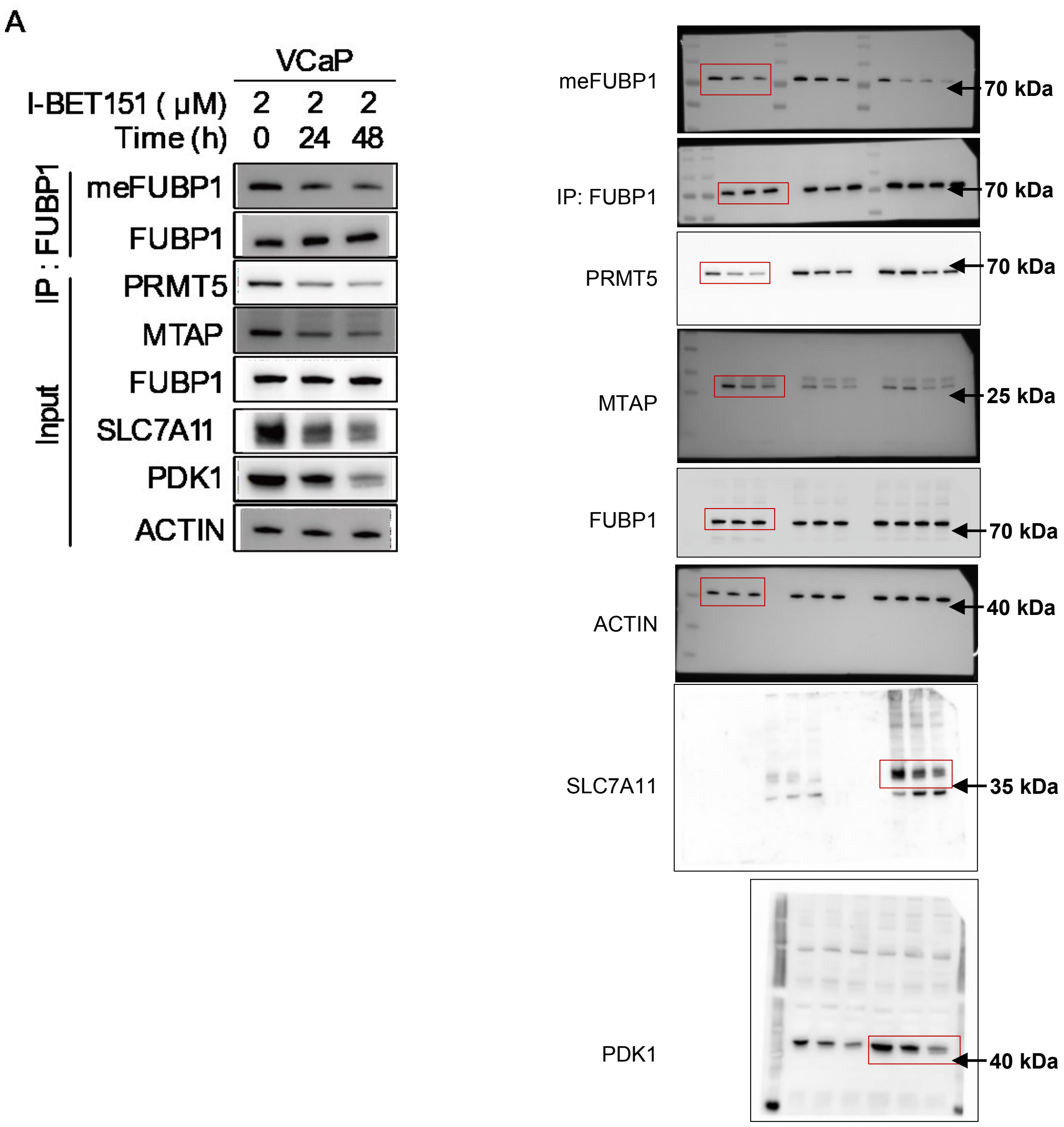
A
VCaP
I-BET151 (μM) 2 2 2
Time (h) 0 24 48
IP : FUBP1
meFUBP1
FUBP1
PRMT5
Input
MTAP
FUBP1
SLC7A11
PDK1
ACTIN
meFUBP1
70 kDa
IP: FUBP1
70 kDa
PRMT5
70 kDa
MTAP
25 kDa
FUBP1
70 kDa
ACTIN
40 kDa
SLC7A11
35 kDa
PDK1
40 kDa

Figure S6D

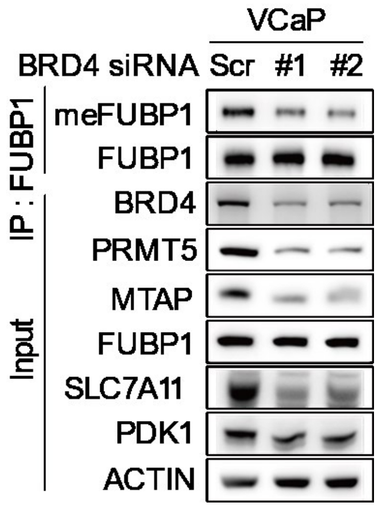

VCaP
BRD4 siRNA Scr #1 #2
IP : FUBP1
meFUBP1
FUBP1
BRD4
PRMT5
MTAP
FUBP1
SLC7A11
PDK1
ACTIN
Input

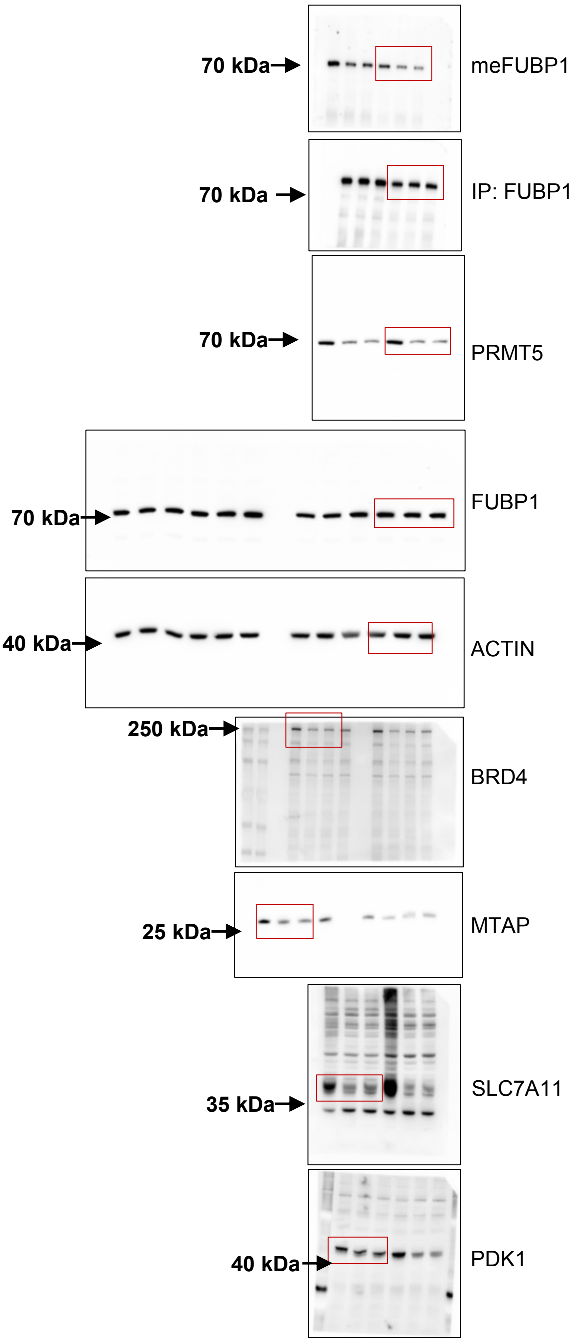

70 kDa
meFUBP1
70 kDa
IP: FUBP1
70 kDa
PRMT5
70 kDa
FUBP1
40 kDa
ACTIN
250 kDa
BRD4
25 kDa
MTAP
35 kDa
SLC7A11
40 kDa
PDK1

Figure S7A

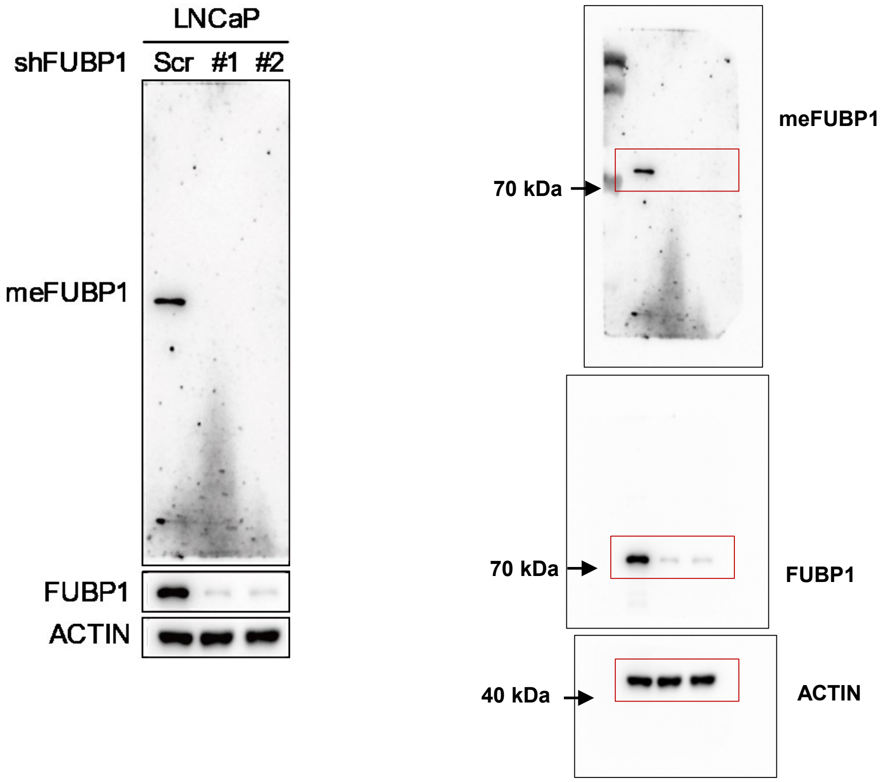
LNCaP
shFUBP1
Scr
#1
#2
meFUBP1
FUBP1
ACTIN
meFUBP1
70 kDa
70 kDa
FUBP1
40 kDa
ACTIN
